# Thioesters as Acyl Donors in Biocatalytic Friedel‐Crafts‐type Acylation Catalyzed by Acyltransferase from *Pseudomonas Protegens*

**DOI:** 10.1002/cctc.201801856

**Published:** 2019-01-09

**Authors:** Anna Żądło‐Dobrowolska, Nina G. Schmidt, Wolfgang Kroutil

**Affiliations:** ^1^ Institute of Chemistry University of Graz NAWI Graz, BioTechMed Graz Graz 8010 Austria; ^2^ ACIB GmbH Graz 8010 Austria

**Keywords:** Friedel-Crafts reaction, thioesters, acyltransferase, acylation, C−C bond formation

## Abstract

Functionalization of aromatic compounds by acylation has considerable significance in synthetic organic chemistry. As an alternative to chemical Friedel‐Crafts acylation, the C‐acyltransferase from *Pseudomonas protegens* has been found to catalyze C−C bond formation with non‐natural resorcinol substrates. Extending the scope of acyl donors, it is now shown that the enzyme is also able to catalyze C−S bond cleavage prior to C−C bond formation, thus aliphatic and aromatic thioesters can be used as acyl donors. It is worth to mention that this reaction can be performed in aqueous buffer. Identifying ethyl thioacetate as the most suitable acetyl donor, the products were obtained with up to >99 % conversion and up to 88 % isolated yield without using additional base additives; this represents a significant advancement to prior protocols.

## Introduction

The design and development of catalytic and regioselective strategies for the C‐acylation of aromatic compounds is of significant importance for organic synthesis.^1^ The resulting aromatic ketones are important building blocks that are widely used for manufacturing products such as pharmaceuticals, agrochemicals, flavors and fragrances.^2^ Functionalization of aromatic compounds *via* Friedel‐Crafts reaction is traditionally achieved by treating the starting material at high temperatures with stoichiometric amounts of Lewis acids (e. g., AlCl_3_, BF_3_, GaCl_3_, BeCl_2_) or Brønsted acids (e. g., HF, H_2_SO_4_, CF_3_COOH, HCl, phosphoric acids).^3^ Either the corresponding acid chloride, anhydride or a carboxylic acid serves as an acyl donor.^4^ In recent years, several improved protocols have been reported for the direct acylation, and most of them employ heterogeneous supports and catalysts (e. g., zeolites, clays, metal oxides, acids, nafion, and graphene),^5^ alternative solvents (e. g., supercritical CO_2_, ionic liquids, hexafluoro‐2‐propanol, deep eutectic solvents)^6^ or activated carboxylic acid equivalents (e. g. twisted amides).^7^

In particular, regioselective C‐acylation of (poly)phenolic substrate remains a challenging task, which is mostly attributed to the electron‐rich nature of the compound causing multiple C‐acylations as well as O‐acylations.^8^ The acylations are mostly performed in organic solvents (e. g., nitrobenzene or nitromethane) or at elevated temperatures of up to 300 °C.^9^ Besides the insufficient reaction control which often leads to product mixtures, another drawback of most chemical methods is the need for hazardous reagents. As an alternative, a biocatalytic approach running the reaction in aqueous media was recently published: originally an acyltransferase from *Pseudomonas fluorescens* was found to be involved in C−C bond formation in the biosynthesis of 2,4‐diacetylphloroglucinol (DAPG).^10^ Subsequently, it was shown that a homologue acyltransferase from *Pseudomonas protegens* (*Pp*ATaseCH) exhibits promiscuous activity, accepting also non‐natural, activated esters, like isopropenyl acetate, phenyl acetate and *N*‐acetyl imidazole for the biocatalytic Friedel‐Crafts acylation of resorcinol derivatives leading to C4‐acylated products.^11^ Thus, the enzyme cleaves a C−O or C−N bond in the acetyl donor and enables subsequently a C−C bond formation to give the final C‐acetylated product. Moreover, *Pp*ATaseCH was identified to catalyze C−N bond formation using aniline derivatives as substrates in aqueous media.^12^

Since thioesters have been reported as outstanding surrogates for carboxylic acid derivatives in a vast number of biotransformations,^13^ we investigated their application in a Friedel‐Crafts‐type bioacylation. According to the best of our knowledge, this class of compounds has never been employed before as acyl donors in the Friedel‐Crafts‐type acylation of aromatic compounds. As an alternative to the commonly employed acyl chlorides and anhydrides in chemical Friedel‐Crafts reactions, a thioester is easy to handle since this class of compound is tolerant to column purification and air stable compared to the corresponding acyl chlorides.^14^

## Results and Discussion

The reactivity of thioesters in comparison to the reactivity of carboxylic acid esters has been of longstanding interest, mainly due to their importance in living organisms.^15^ Whereas in organic synthetic chemistry, thioester formation is a common strategy to activate carboxylic acid building blocks leading to activated acyl units, which are fused with other chemical building blocks, mostly by C−C, C−O or C−N bond formation.^16^ Now, aliphatic and aromatic thioesters **2** **a**–**d** were investigated as acyl donors in the Friedel‐Crafts‐type biocatalytic reaction. For this purpose, the substrate resorcinol (**1** **a**, 10 mM) was suspended in KPi‐buffer adjusted to pH 7.5, and cell‐free extract containing recombinant *Pp*ATaseCH was subsequently added, followed by addition of the acetyl donor (100 mM, **2** **a**–**d**). Transformations were studied in the presence and absence of imidazole as an additive, since imidazole was previously identified to promote the bioacylation using either IPEA or vinyl acetate.^11^

All tested thioesters afforded the product 2,4‐dihydroxyacetophenone (**3** **a**) in good to excellent yields (58–>99 %, Table 1). Thus, in the observed reaction the enzyme breaks a C−S bond in the acetyl donor and enables subsequently C−C bond formation. Acetylation was observed exclusively at C4 position of **3** **a**, thus neither multiple substitution nor O‐acylation was detectable after 18 h of reaction. C‐acylation in the absence of imidazole using aliphatic thioesters, such as ethyl thioacetate (**2** **a**) or 2‐acetylthioacetophenone (**2** **d**) led to very high conversions, 81 % and 93 % respectively. Moderate conversion was observed using *S*‐4‐nitrobenzyl thioacetate (**2** **c**, 58 %). *S*‐Phenyl thioacetate (**2** **b**) turned out to be a very suitable donor substrate leading to product **3** **a** with 95 % yield. In all cases, imidazole as an additive improved the reaction leading to complete consumption of substrate (up to >99 %, Table 1). It is worth to mention, that performing the reaction with the natural acetyl thioester acetyl‐CoA as possible donor did not lead to any detectable conversion.

**Table 1:** *Pp*AtaseCH catalyzed bioacylation of resorcinol (**1**) employing aromatic and aliphatic thioesters as acyl donors in the absence and presence of imidazole.

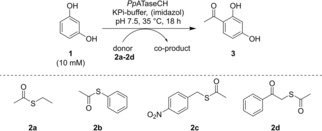			
Entry	Donor	HPLC conversion with Im [%]	HPLC conversion without Im [%]
1	**2 a**	>99	81
2	**2 b**	>99	95
2	**2 c**	>99	58
3	**2 d**	>99	93

Reaction conditions: cell‐free *E. coli* extract containing *Pp*ATaseCH (0.066 U) in KPi‐buffer (100 mM, pH 7.5, total volume 1 mL), resorcinol (**1 a**, 0.01 mmol), donor **2 a–2 d** (0.1 mmol) with or without imidazole (100 mM added from a 1 M stock solution prepared in the reaction buffer), 35 °C, 18 h. Experiments were performed in duplicate.

**Figure 1:**
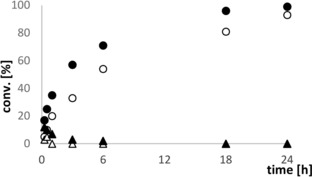
Time course for the *Pp*ATaseCH‐catalyzed acetylation of **1** **a** (10 mM) with ethyl thioacetate (**2** **a**, 100 mm) leading to **3** **a** in the presence (•) or absence (○) of imidazole and *O*‐acetylated co‐product **4** **a**, in the presence (▴) or absence (Δ) of imidazole.

Comparing the pH of the reaction mixture before and after imidazole addition revealed a shift from 7.5 to 8.3. For comparison, a reaction performed in buffer adjusted to pH 8.3 in the absence of imidazole led to lower conversion compared to reaction performed in the presence of imidazole (Table 2). To gain an insight into the imidazole effect, the time course of the reaction was monitored (Figure 1). Although imidazole promotes the reaction reaching completion within 24 h, the reaction in the absence of imidazole was slightly slower requiring approximately 30 h for completion. Importantly, *O*‐acetylated product formation (**4** **a**) was observed only at the initial stage of the reaction and its formation was much more pronounced in the presence of imidazole.

**Table 2:** Influence of amine additives on the C−C bioacylation of **1 a**.

Entry	Donor	Additive	Additive concentration [mM]	pH	Yield [%]
1	**2 a**	none	0	7.5	81
2	**2 a**	Imidazole	10	7.6	79
3	**2 a**	Imidazole	50	8	89
4	**2 a**	Imidazole	100	8.3	>99
5	**2 b**	none	0	7.5	95
6	**2 b**	Imidazole	10	7.6	93
7	**2 b**	Imidazole	50	8	93
8	**2 b**	Imidazole	100	8.3	96
9	**2 a**	DABCO	10	8.4	89
10	**2 a**	DABCO	50	9.6	95
11	**2 a**	DABCO	100	10	99
12	**2 b**	DABCO	10	8.4	91
13	**2 b**	DABCO	50	9.6	91
14	**2 b**	DABCO	100	10	91
15	**2 a**	none	0	8.3	74

Reaction conditions: cell‐free *E. coli* extract containing *Pp*ATaseCH (0.066 U) in KPi‐buffer (100 mM, pH 7.5, total volume 1 mL), resorcinol (**1**, 0.01 mmol), donor **2 a** or **2 b** (0.01 mmol–0.1 mmol) with or without imidazole or DABCO (100 mM added from a 1 M stock solution prepared in the reaction buffer), 35 °C, 18 h.

Since various organic amines have been reported to enhance the Friedel‐Craft‐like biotransformation,^17^ the influence of DABCO additionally to imidazole was examined. The acetylation was performed at varied DABCO or imidazole concentrations (Table 2). A 10‐fold decrease of imidazole or DABCO concentration from 100 mM to 10 mM using **2** **a** led only to a slightly lower conversion, 79 % and 89 % respectively (entries 2 and 9). In contrast, no effect was observed for **2** **b** (entries 5–8 and 12–14), which seemed to be insensitive for imidazole or DABCO addition. This is an important observation, since in comparison to carboxylic acid esters as donors,^11^ thioesters work without amine additives. Therefore, the environmental footprint is reduced since no amine additive is required. Consequently, ethyl thioacetate was chosen as acyl donor for further experiments. According to the best of our knowledge it is the first report employing thioesters as alternative acyl donors in Friedel‐Crafts‐type acylation.

Subsequently, process intensification was investigated. Table 3 summarizes key optimization results employing ethyl thioacetate as an acyl donor. First, the concentration of thioester was decreased from 100 mM to 50 mM at constant enzyme (0.066 U) and substrate concentration (10 mM) leading to a conversion of 60 % and a space time yield of 51 g L^−1^ h^−1^ (Entry 2). At lower acetyl donor concentration (15 mM) the conversion dropped to just 22 % (Entry 1). Increasing the concentration of the acetyl acceptor (resorcinol) from 10 mM to 20 mM and keeping the enzyme and donor concentrations constant, led to lower conversion (46 %) however accompanied by higher space‐time yield (78 g L^−1^ h^−1^). To increase further the space time yield, a 2‐fold concentrated bioconversion was run at 20 mM substrate **1** **a** with 200 mM donor **2** **a**. After 18 h space time yield reached 86 g L^−1^ h^−1^ compared to 68 in the control (Table 3, entry 5 vs. 3). Increasing the enzyme amount up to 0.132 U improved space time yield to 105 g L^−1^ h^−1^ despite the moderate conversion (62 %, entry 6). Because of the sensitivity of this enzyme towards high substrate loadings, 10 mM substrate with 100 mM donor was chosen as the conditions for the transformation of further substrate and scale up.

**Table 3:** Monitoring the progress of the bioacetylation of resorcinol (**1 a**) with ethyl thioacetate (**2 a**) as acyl donor.

Entry	Reaction Time [h]	Enzyme [U]	Donor conc. [mM]	Substrate conc. [mM]	HPLC yield **3** [%]	Space time yield **3** [g L^−1^ h^−1^]
1	18	0.066	15	10	22	19
2	18	0.066	50	10	60	51
3	18	0.066	100	10	81	68
4	18	0.066	100	20	46	78
5	18	0.066	200	20	51	86
6	18	0.132	200	20	62	105

Reaction conditions: cell‐free *E. coli* extract containing *Pp*ATaseCH (0.066–0.132 U) in KPi‐buffer (100 mM, pH 7.5) with the acceptor **1** (10–20 mM) and the donor **2 a** (15–200 mM) as indicated below, 35 °C, 18 h, 750 rpm.

Using ethyl thioacetate as acetyl donor, a broad set of acyl acceptors **1** **a**–**j** were tested. Since the reaction rates in the absence of imidazole were slower, reaction times were elongated up to 24 h, so that the bioacylation of **1** **a** reached 96 % conversion (Table 4). Resorcinols bearing chloro‐substituent (**3** **f**) or different lengths of alkyl chains at position C6 (**3** **b**–**3** **e**) were accepted providing products with conversions up to 40 %. Compound with a methyl group at C6 underwent lower conversion (**3** **b**, 7 %) than either ethyl (**3** **c**, 32 %), *n*‐butyl (**3** **d**, 30 %) or *n*‐hexyl group (**3** **e**, 25 %). In all cases, imidazole addition improved conversions significantly, for instance for **3** **d** the conversion was improved from 30 % to 90 %. Resorcinol derivatives possessing methoxy (**3** **g**) or chloro group (**3** **h**) at position C5 were not accepted as substrates. Furthermore, aniline (**1** **i**) and 3‐hydroxyaniline (**1** **j**) were examined as acyl acceptors. In this case a C−N instead of a C−C bond was formed, thus leading to the corresponding acetanilides with up to >99 % conversion. In the presence of imidazole as an additive, reaction reached also completion, however background reaction was observed.

**Table 4:** Acetylation of various substrates using ethyl thioacetate and semi‐preparative experiments.

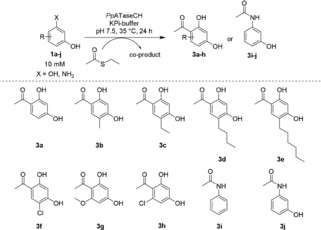			
Entry	Product	w/o Im [%]	Im [%]
1	**3 a**	96 (88)	>99
2	**3 b**	7	16
3	**3 c**	32	50 (49)
4	**3 d**	30	90 (70)
5	**3 e**	25	34 (37)
6	**3 f**	40	51 (45)
8	**3 g**	<1	<1
9	**3 h**	<1	<1
10	**3 i**	79 (37)	>99
11	**3 j**	>99 (58)	>99

Reaction conditions: cell‐free *E. coli* extract containing *Pp*ATaseCH (0.066 U) in KPi‐buffer (100 mM, pH 7.5) with the acceptor **1** (10 mM) and the donor **2 a** (100 mM) with or without imidazole (100 mM added from a 1 M stock solution prepared in the reaction buffer), 35 °C, 24 h, 750 rpm; conversions were determined by HPLC, values within parentheses refer to yields of isolated products.

Finally, reaction scale‐up was performed in 25 mL volume using 10 mM substrate (values in brackets, Table 4) to avoid potential enzyme inhibition. In the case of substrate **1** **a**, 2,4‐dihydroxyacetophenone (**3** **a**) was isolated with 88 % yield (33.6 mg), highlighting excellent acyl transfer efficiency. Acetanilide **3** **i** and **3** **j** were isolated with up to 58 % (12.4 mg, 22 mg, respectively). Since for C6‐substituted compounds moderate conversions were observed on analytical scale, scale‐up was run in 100 mM imidazole solution. The isolated product yields from preparative scale experiments were comparable to those determined on analytical scale. In case of products **3** **e** and **3** **f** an additional hydrolysis step was necessary since after biotransformation a mixture of C‐acylated and O‐acylated products was obtained.

## Conclusions

A range of commercially available thioesters were evaluated as acyl donors for the biocatalytic Friedel‐Crafts‐type acetylation of resorcinol derivatives. Ethyl thioester was identified as a very suitable acyl donor, which allowed the synthesis of valuable aromatic ketones with moderate to high isolated yields (up to >99 % conv. and 88 % isolated yield). The method presented is broadly applicable to resorcinol and its derivatives, tolerating a wide range of functional groups at position C6, while substitution at C5 is not tolerated. Bioconversions may be run at 20 mM or higher providing satisfactory space time yields. Importantly, in contrast to previous protocols no imidazole as additive is required, which makes this method a feasible new tool for the toolbox of biocatalytic C−C bond formation.^18^, ^19^

## Experimental Section

**General Methods**. ^1^H‐ and ^13^C‐NMR spectra were recorded in CDCl_3_, DMSO‐*d6* or acetone‐*d6* solution. Chemical shifts are expressed in parts per million using TMS as an internal standard. TLC was done on Kieselgel 60 F254 aluminum sheets. Reference compounds: **3 b**, **3 c**, **3 d**, **3 e**, **3 f** were synthesized according to the general procedure, reference compounds **3 a**, **3 i**, **3 j** were commercial products of analytical grade.

**General Procedure for Chemical Friedel‐Crafts Acylation**. The corresponding resorcinol derivative **1 a**–**1 f** (1 equiv.) was dissolved by dropwise adding BF_3_⋅2CH_3_COOH (2.5 mL, 18.0 mmol). The resulting solution was stirred and refluxed for 3 to 4 h. After cooling the reaction mixture to room temperature 0.5 M aqueous KOAc (50 mL) was added dropwise and stirring was continued for further 30 minutes. The crude precipitate was filtered and recrystallized from MeOH/H_2_O (1 : 1, 60 mL) affording the 2,4‐dihydroxyacetophenone analogs **3 b**–**3 f**. Selected compounds were additionally purified by column chromatography.

**Screening Procedure**. Resorcinol derivative **1 a**–**1 h** or aniline derivative **1 i**–**1 j** (0.01 mmol, 10 mM final concentration) was suspended in potassium phosphate buffer (100 mM, pH 7.5). Then, cell‐free extract of recombinant ATase (0.066 U) was added to the reaction mixture. The bioacylation was started by addition of the donor **2 a**–**2 d** (0.1 mmol, 100 mM final concentration) and amine additive (imidazole or DABCO, 100 mM final concentration, added from 1 M stock solution prepared in the reaction buffer). The reaction mixture was shaken for 18–24 h at 35 °C and 750 rpm in an orbital shaker. Reaction was quenched by addition of acetonitrile (1 mL). The precipitated protein was removed by centrifugation (30 min, 14,000 rpm) and the supernatant was subjected to HPLC for determination of conversions. As a negative control, reactions without enzyme were performed. All experiments were performed in duplicate (Table 1) or as a single study (Tables 3–4).

**Semi‐preparative Scale Friedel‐Crafts Bioacylation**. Resorcinol derivative **1 a**–**1 f** or aniline derivative **1 i**–**1 j** (0.25 mmol, 10 mM final concentration) was dissolved in potassium phosphate buffer (100 mM, pH 7.5) in a shaking flask. Cell‐free extract containing the *Pp*ATaseCH (2.5 mL, 1.65 U) was added to the reaction mixture followed by ethyl thioacetate (266 μL, 2.5 mmol, 100 mM final concentration) addition. For substrates **1 c**–**1 f**, imidazole (2.5 mmol, 100 mM final concentration, added from a 1 M stock solution prepared in the reaction buffer) was added. The bioacetylation (25 mL total volume) was run at 35 °C and 120 rpm for 24 h. The resulting suspension was extracted with ethyl acetate (2×20 mL), centrifuged (5 min, 4,000 rpm). Then organic layers were separated, combined and dried over anhydrous MgSO_4_. Solvent was removed under reduced pressure and crude product was purified by column chromatography using silica gel, DCM/MeOH or cyclohexane/EtOAc as an eluent. Compounds were characterized by ^1^H NMR and ^13^C NMR and GC‐MS and the chemical identity was confirmed by comparison to literature. For compounds **3 e** and **3 f** a mixture of C‐acylated and O‐acylated products was observed. To remove O‐acylated products, mixture was submitted to hydrolysis with 1 N NaOH for 1 h at rt, then acidified with 1 N HCl, extracted with EtOAc and purified by column chromatography.

### 1‐(2,4‐dihydroxyphenyl)ethan‐1‐one (3 a)

^1^H NMR (300 MHz, DMSO‐*d6*): δ [ppm]=12.61 (s, 1H), 10.63 (s, 1H), 7.75 (d, *J*=8.8 Hz, 1H), 6.38 (dd, *J*=8.8, 2.4 Hz, 1H), 6.24 (d, *J*=2.3 Hz, 1H), 2.52 (s, 3H); ^13^C NMR (75 MHz, DMSO‐*d6*): δ [ppm]=203.17, 165.34, 164.66, 134.18, 113.31, 108.57, 102.74, 26.82; GC‐MS (EI^+^, 70 eV): m/z (%)=152.1 [M^+^] (47), 137.0 [C_7_H_5_O_3_^+^] (100), 109 [C_6_H_5_O_2_^+^] (3).

### 1‐(5‐ethyl‐2,4‐dihydroxyphenyl)ethan‐1‐one (3 c)

^1^H NMR (300 MHz, CDCl_3_): δC [ppm]=12.62 (s, 1H), 7.47 (s, 1H), 6.35 (s, 1H), 2.65–2.54 (m, 5H), 1.24 (t, *J*=7.5 Hz, 3H); ^13^C NMR (75 MHz, CDCl_3_): δC [ppm]=202.91, 163.10, 161.25, 131.58, 122.64, 113.88, 103.10, 77.44, 77.02, 76.59, 26.18, 22.47, 14.07. GC‐MS (EI^+^, 70 eV): m/z (%)=180.1 [M^+^] (36), 165.0 [C_9_H_9_O_3_^+^] (100).

### 1‐(5‐butyl‐2,4‐dihydroxyphenyl)ethan‐1‐one (3 d)

^1^H NMR (300 MHz, Acetone‐*d6*): δC [ppm]=12.61 (s, 1H), 9.37 (s, 1H), 7.65 (s, 1H), 6.35 (s, 1H), 2.64–2.52 (m, 5H), 1.67–1.48 (m, 2H), 1.38 (dq, *J*=14.4, 7.2 Hz, 2H), 0.94 (t, *J*=7.3 Hz, 3H); ^13^C NMR (75 MHz, Acetone‐*d6*): δC [ppm]=202.71, 163.35, 162.56, 132.67, 121.33, 113.13, 102.26, 32.05, 28.96, 25.36, 22.26, 13.34; GC‐MS (EI^+^, 70 eV): m/z (%)=208.1 [M^+^] (25), 193.1 [C_11_H_13_O_3_^+^] (13), 165.0 [C_9_H_9_O_3_^+^] (100).

### 1‐(5‐hexyl‐2,4‐dihydroxyphenyl) ethan‐1‐one (3 e)

^1^H NMR (300 MHz, Acetone‐*d6*) δ 12.61 (s, 1H), 9.40 (s, 1H), 7.65 (s, 1H), 6.35 (s, 1H), 2.66–2.50 (m, 5H), 1.70–1.49 (m, 2H), 1.42–1.25 (m, 6H), 0.89 (dd, *J*=8.8, 5.2 Hz, 3H); ^13^C NMR (75 MHz, Acetone‐*d6*): δ [ppm]=202.69, 163.36, 162.59, 132.66, 121.38, 113.12, 102.26, 31.57, 29.80, 29.69, 29.26, 25.37, 22.40, 13.44; GC‐MS (EI^+^, 70 eV): m/z (%)=236.1 [M^+^] (15), 221.1 [C_13_H_17_O_3_^+^] (7), 165.0 [C_9_H_9_O_3_^+^] (100).

### 1‐(5‐chloro‐2,4‐dihydroxyphenyl)ethan‐1‐one (3 f)

^1^H NMR (300 MHz, Acetone‐*d6*): δ [ppm]=12.58 (s, 1H), 9.99 (s, 1H), 7.91 (s, 1H), 6.51 (s, 1H), 2.62 (s, 3H); ^13^C NMR (75 MHz, Acetone‐*d6*): δ [ppm]=202.72, 163.37, 159.70, 132.54, 113.94, 111.52, 103.85, 25.55; GC‐MS (EI^+^, 70 eV): m/z (%)=186.0 [M^+^] (41), 171.0 [C_7_H_4_ClO_3_^+^] (100).

### *N*‐phenylacetamide (3 i)

^1^H NMR (300 MHz, CDCl_3_): δ [ppm]=7.52 (d, *J*=7.9 Hz, 2H), 7.34 (t, *J*=7.9 Hz, 2H), 7.12 (t, *J*=7.4 Hz, 1H), 2.19 (s, 3H); ^13^C NMR (75 MHz, CDCl_3_): δ [ppm]=168.24, 137.85, 128.99, 124.30, 119.85, 24.60; GC‐MS (EI^+^, 70 eV): m/z (%)=135.1 [M^+^] (27), 93.1 [C_6_H_7_N^+^] (100).

### N‐(3‐hydroxyphenyl)acetamide (3 j)

^1^H NMR (300 MHz, DMSO‐*d6*): δ [ppm] 9.77 (s, 1H), 9.31 (s, 1H), 7.18 (t, *J*=1.9 Hz, 1H), 7.04 (t, *J*=8.0 Hz, 1H), 6.92 (d, *J*=8.1 Hz, 1H), 6.49–6.32 (m, 1H), 2.01 (s, 3H); ^13^C NMR (75 MHz, DMSO‐*d6*): δ [ppm] 168.58, 157.98, 140.80, 129.69, 110.54, 110.19, 106.61, 24.50; GC‐MS (EI^+^, 70 eV): m/z (%)=151.1 [M^+^] (44), 109.1 [C_6_H_7_NO^+^] (100).

## Conflict of interest

The authors declare no conflict of interest.

## Supporting information

As a service to our authors and readers, this journal provides supporting information supplied by the authors. Such materials are peer reviewed and may be re‐organized for online delivery, but are not copy‐edited or typeset. Technical support issues arising from supporting information (other than missing files) should be addressed to the authors.

Supplementary

## References

[1a] Y. H. Lee , B. Morandi , Nat. Chem. 2018, 10, 116–117; 29359750 10.1038/nchem.2934

[1b] Z. Sadiq , M. Iqbal , E. A. Hussain , S. Naz , J. Mol. Liq. 2018, 255, 26–42;

[1c] G. A. El-Hiti , K. Smith , A. S. Hegazy , Curr. Org. Chem. 2015, 19, 585–598.

[2a] W. Shi , W. J. Dan , J. J. Tang , Y. Zhang , T. Nandinsuren , A. L. Zhang , J. M. Gao , Bioorg. Med. Chem. Lett. 2016, 26, 2156–2158; 27025344 10.1016/j.bmcl.2016.03.073

[2b] G. Yu , D. Kuo , M. Shoham , R. Viswanathan , ACS Comb. Sci. 2013, 16, 85–91; 10.1021/co400142t 24372007

[2c] F. A. A. van Acker , J. A. Hageman , G. R. M. M. Haenen , W. J. F. van der Vijgh , A. Bast , W. M. P. B. Menge , J. Med. Chem. 2000, 43, 3752–3760; 11020290 10.1021/jm000951n

[2d] M. D. Hilton , W. J. Cain , Appl. Environ. Microbiol. 1990, 56, 623–627. 16348137 10.1128/aem.56.3.623-627.1990 PMC183396

[3] F. Effenberger , G. Epple , Angew. Chem. Int. Ed. 1972, 11, 300–301.

[4a] M. Rueping , B. J. Nachtsheim , Beilstein J. Org. Chem. 2010, 6, 1–24; 20485588 10.3762/bjoc.6.6 PMC2870981

[4b] G. Sartori , R. Maggi in Advances in Friedel-Crafts Acylation Reactions: Catalytic and Green Processes, 1^st^ Edition, CRC Press, Boca Raton, 2009, pp. 9–18.

[5a] T. Yamazaki , M. Makihara , K. Komura , J. Mol. Catal. A 2017, 426, 170–176;

[5b] J. C. Kim , K. Cho , S. Lee , R. Ryoo , Catal. Today 2015, 243, 103–108;

[5c] F. Hu , M. Patel , F. Luo , C. Flach , R. Mendelsohn , E. Garfunkel , H. He , M. Szostak , J. Am. Chem. Soc. 2015, 137, 14473–14480; 26496423 10.1021/jacs.5b09636

[5d] M. H. Sarvari , H. Sharghi , J. Org. Chem. 2004, 69, 6953–6956; 15387635 10.1021/jo0494477

[5e] T. Yamato , C. Hideshima , G. K. S. Prakash , G. A. Olah , J. Org. Chem. 1991, 56, 3955–3957.

[6a] R. H. Vekariya , J. Aube , Org. Lett. 2016, 18, 3534–3537; 27458880 10.1021/acs.orglett.6b01460

[6b] Z. P. Wang , J. Y. Wang , J. R. Li , M. L. Feng , G. D. Zou , X. Y. Huang , Chem. Commun. 2015, 51, 3094; 10.1039/c4cc08825e 25597829

[6c] N. Aribert , S. Camy , Y. P. Lucchese , J.-S. Condoret , P. Cognet , International Journal of Chemical Reactor Engineering 2010, 8.

[7] Y. Liu , G. Meng , R. Liu , M. Szostak , Chem. Commun. 2016, 52, 6841–6844. 10.1039/c6cc02324j 27139813

[8] R. Murashige , Y. Hayashi , S. Ohmori , A. Torii , Y. Aizu , Y. Muto , Y. Murai , Y. Oda , M. Hashimoto , Tetrahedron 2011, 67, 641–649.

[9] J. S. Brown , R. Gläser , C. L. Liotta , C. A. Eckert , Chem. Commun. 2000, 1295–1296.

[10a] J. Almario , M. Bruto , J. Vacheron , C. Prigent-Combaret , Y. Moënne-Loccoz , D. Muller , Front. Microbiol. 2017, 8, 1218; 28713346 10.3389/fmicb.2017.01218 PMC5491608

[10b] A. Hayashi , H. Saitou T. Mori , I. Matano , H. Sugisaki , K. Maruyama , Biosci. Biotechnol. Biochem. 2014, 76, 559–566; 10.1271/bbb.110860 22451400

[10c] F. Yang , Y. Cao , Appl. Microbiol. Biotechnol. 2012, 93, 487–495. 22101786 10.1007/s00253-011-3712-6

[11a] N. G. Schmidt , A. Żądło-Dobrowolska , V. Ruppert , C. Höflehner , B. Wiltschi , W. Kroutil , Appl. Microbiol. Biotechnol. 2018, 1–12; 10.1007/s00253-018-9052-z PMC6013524 29754162

[11b] N. G. Schmidt , T. Pavkov-Keller , N. Richter , B. Wiltschi , K. Gruber , W. Kroutil , Angew. Chem. Int. Ed. 2017, 56, 7615–7619; 10.1002/anie.201703270 PMC5488191 28544673

[11c] N. G. Schmidt , W. Kroutil , Eur. J. Org. Chem. 2017, 39, 5865–5871.

[12] A. Żądło-Dobrowolska , N. G. Schmidt , W. Kroutil , Chem. Commun. 2018, 54, 3387–3390. 10.1039/c8cc00290h PMC5885802 29553154

[13a] S. H. Younes , Y. Ni , S. Schmidt , W. Kroutil , F. Hollmann , ChemCatChem 2017, 9, 1389–1392;

[13b] P. Falus , L. Cerioli , G. Bajnóczi , Z. Boros , D. Weiser , J. Nagy , J. D. Tessaro , S. Servi , L. Poppe , Adv. Synth. Catal. 2016, 358, 1608–1617;

[13c] D. Tessaro , L. Cerioli , S. Servi , F. Viani , P. D′Arrigo , Adv. Synth. Catal. 2011, 353, 2333–2338;

[13d] N. Weber , E. Klein , K. Vosmann , K. D. Mukherjee , APP Australas. Plant Pathol. 2004, 64, 800–805; 10.1007/s00253-004-1604-8 15048592

[13e] P. J. Um , D. G. Drueckhammer , J. Am. Chem. Soc. 1998, 120, 5605–5610.

[14] M. N. Burhardt , A. Ahlburg , T. Skrydstrup , J. Org. Chem. 2014, 79, 11830–11840. 24919457 10.1021/jo5009965

[15a] K. L. Dunbar , D. H. Scharf , A. Litomska , C. Hertweck , Chem. Rev. 2017, 117, 5521–5577; 28418240 10.1021/acs.chemrev.6b00697

[15b] J. Franke , C. Hertweck , Cell Chem. Biol. 2016, 23, 1179–1192; 27693058 10.1016/j.chembiol.2016.08.014

[15c] W. Yang , D. G. Drueckhammer , J. Am. Chem. Soc. 2001, 123, 11004–11009; 11686705 10.1021/ja010726a

[15d] C. de Duve , The Molecular Origins of Life 1998, 219–236.

[16a] J. Guang , A. J. Larson , J. C. G. Zhao , Adv. Synth. Catal. 2015, 357, 523–529;

[16b] K. Yan , D. Yang , W. Wei , J. Zhao , Y. Shuai , L. Tian , H. Wang , Org. Biomol. Chem. 2015, 13, 7323–7330. 26058949 10.1039/c5ob00769k

[17a] Y. R. Liang , Q. Wu , X. F. Lin , The Chemical Record 2017, 17, 90–121; 27490244 10.1002/tcr.201600016

[17b] B. K. Liu , Q. Wu , D. S. Lv , X. F. Lin , J. Biotechnol. 2011, 153, 111–115; 21419813 10.1016/j.jbiotec.2011.03.009

[17c] B. K. Liu , Q. Wu , J. M. Xu , X. F. Lin , Chem. Commun. 2007, 295–297. 10.1039/b611454g 17299644

[18] For reviews see:

[18a] N. G. Schmidt , E. Eger , W. Kroutil , ACS Catal. 2016, 6, 4286–4311; 27398261 10.1021/acscatal.6b00758 PMC4936090

[18b] K. Faber , W. D. Fessner , N. J. Turner , Biocatalysis in Organic Synthesis. Science of Synthesis., Vol. 1–3, Thieme, Stuttgart, 2015;

[18c] K. Fesko , M. Gruber-Khadjawi , ChemCatChem 2013, 5, 1248–1272;

[19a] S. Junker , R. Roldan , H.-J. Joosten , P. Clapés , W.-D. Fessner , Angew. Chem. Int. Ed. 2018, 57, 10153–10157; 10.1002/anie.201804831 PMC6099348 29882622

[19b] G. A. Aleku , B. Nowicka , N. J. Turner , ChemCatChem 2018, 10, 124–135;

[19c] V. Laurent , E. Darii , A. Aujon , M. Debacker , J.-L. Petit , V. Hélaine , T. Liptaj , M. Breza , A. Mariage , L. Nauton , M. Traikia , M. Salanoubat , M. Lemaire , C. Guérard-Hélaine , V. de Berardinis , Angew. Chem. Int. Ed. 2018, 57, 5467–5471; 10.1002/anie.201712851 29542859

[19d] A. Szekrenyi , X. Garrabou , T. Parella , J. Joglar , J. Bujons , P. Clapés , Nat. Chem. 2015, 7, 724–729; 26291944 10.1038/nchem.2321

[19e] J.-Y. van der Meer , H. Poddar , B.-J. Baas , Y. Miao , M. Rahimi , A. Kunzendorf , R. van Merkerk , P. G. Tepper , E. M. Geertsema , A.-M. W. H. Thunnissen , W. J. Quax , G. J. Poelarends , Nat. Commun. 2016, 7, 10911; 26952338 10.1038/ncomms10911 PMC4786785

[19f] B. R. Lichman , E. D. Lamming , T. Pesnot , J. M. Smith , H. C. Hailes , J. M. Ward , Green Chem. 2015, 17, 852–855

