# Supplementary material for: Thioesters as Acyl Donors in Biocatalytic Friedel‐Crafts‐type Acylation Catalyzed by Acyltransferase from Pseudomonas Protegens
Source: ChemCatChem. 2019 Jan 9;11(3):1064–8. doi: 10.1002/cctc.201801856 (PMC6686624; doi:10.1002/cctc.201801856)
Supplement: Supplementary file 1 — Supplementary [file CCTC-11-1064-s001.pdf]

Supporting Information

© Copyright Wiley-VCH Verlag GmbH & Co. KGaA, 69451 Weinheim, 2019

Thioesters as Acyl Donors in Biocatalytic Friedel-Crafts-type Acylation Catalyzed by Acyltransferase from *Pseudomonas Protegens*

Anna Żądło-Dobrowolska, Nina G. Schmidt, and Wolfgang Kroutil*© 2018 The Authors. Published by Wiley-VCH Verlag GmbH & Co. KGaA.
This is an open access article under the terms of the Creative Commons Attribution Non-Commercial License, which permits use, distribution and reproduction in any medium, provided the original work is properly cited and is not used for commercial purposes.

Supporting Information

Thioesters as acyl donors in biocatalytic Friedel-Crafts-type acylation catalyzed by acyltransferase from *Pseudomonas protegens*

Anna Żądło-Dobrowolska,[1] Nina G. Schmidt,[1,2] Wolfgang Kroutil*[1,2]

[1]Institute of Chemistry, University of Graz, NAWI Graz, BioTechMed Graz, Graz, Austria

[2]ACIB GmbH, Graz, Austria

Corresponding Authors: wolfgang.kroutil@uni-graz.at

Table of Contents:

Materials

Methods

General Procedure for Chemical Friedel-Crafts Acylation

Reference compound: 1-(2,4-dihydroxy-5-methylphenyl)ethan-1-one (Ref-**3b**)

Reference compound: 1-(5-ethyl-2,4-dihydroxyphenyl)ethan-1-one (Ref-**3c**)

Reference compound: 1-(5-butyl-2,4-dihydroxyphenyl)ethan-1-one (Ref-**3d**)

Reference compound: 1-(5-hexyl-2,4-dihydroxyphenyl)ethan-1-one (Ref-**3e**)

Reference compound: 1-(5-chloro-2,4-dihydroxyphenyl)ethan-1-one (Ref-**3f**)

Activity Assay

Screening Procedure

Semi-preparative Scale Friedel-Crafts Bioacylation

1-(2,4-dihydroxyphenyl)ethan-1-one (**3a**)

1-(2,4-dihydroxy-5-methylphenyl)ethan-1-one (**3b**)

1-(5-ethyl-2,4-dihydroxyphenyl)ethan-1-one (**3c**)

1-(5-butyl-2,4-dihydroxyphenyl)ethan-1-one (**3d**)

1-(5-hexyl-2,4-dihydroxyphenyl)ethan-1-one (**3e**)

1-(5-chloro-2,4-dihydroxyphenyl)ethan-1-one (**3f**)

N-phenylacetamide (**3i**)

N-(3-hydroxyphenyl)acetamide (**3j**)

Materials

All starting materials were obtained from Sigma-Aldrich, Alfa Aesar or TCI-Chemicals and used as received unless stated otherwise. Reference compounds: 1-(2,4-dihydroxyphenyl)ethan-1-one (**3a**), *N*-(3-hydroxyphenyl)acetamide (**3j**), *N*-phenylacetamide (**3i**) are commercially available. Reference compounds: 1-(2,4-dihydroxy-5-methylphenyl)ethan-1-one (**3b**), 1-(5-ethyl-2,4-dihydroxyphenyl)ethan-1-one (**3c**), 1-(5-butyl-2,4-dihydroxyphenyl)ethan-1-one (**3d**), 1-(5-hexyl-2,4-dihydroxyphenyl)ethan-1-one (**3e**), 1-(5-chloro-2,4-dihydroxyphenyl)ethan-1-one (**3f**) were chemically synthesized.

Methods

TLC was carried out with pre-coated aluminum sheets (TLC Silica gel 60 F254, Merck) with detection by UV (254 nm) and/or by staining with cerium molybdate solution. GC-MS spectra were recorded with an Agilent 7890A GC-system, equipped with an Agilent 5975C mass selective detector and a HP-5 MS column (30 m × 0.25 mm × 0.25 µm). Analysis was performed according to the following parameters: injector 250 °C, constant flow 0.7 mL; temperature program: 100 °C (hold 0.5 min) 100 °C to 300 °C (10 °C min^{-1}), 300 °C (hold 2 min). Helium was used as carrier gas and EtOAc was used as solvent. ^{1}H- and ^{13}C-NMR spectra were recorded at 20 °C on a 300 MHz Bruker NMR or 500 MHz Bruker NMR.

The conversions of all biotransformations were measured at 25 °C by HPLC using an Agilent 1260 Infinity HPLC system, equipped with a diode array detector (G4212B) and a Phenomenex Luna® 5µ C18 (2) 100A (250 × 4.6 mm) column. Reactions were eluted with a H_2O/MeCN-gradient (+TFA, 0.1 vol%). Method A: 0-15% MeCN (0-5 min), 15-60% MeCN (5-22 min), 60-100% MeCN (22-25 min), 100-0% MeCN (25-30 min), flow rate = 1 mL min^{-1}, sample vol. = 2 µL; method B: 0-15 % MeCN (0-10 min), 15-60 % MeCN (10-45 min), 60-100 % MeCN (45-55 min), 100-0 % MeCN (55-60 min), flow rate = 1 mL min^{-1}, λ = 254 nm, injection vol. = 2 µL Reaction products were quantified at 254 nm from the peak areas on the basis of standard curves with reference compounds. The ATase from *Pseudomonas protegens* (*Pp*ATaseCH) was overexpressed in *E. coli* BL21 (DE3) as described previously and used as cell-free extract preparations.[1]

General Procedure for Chemical Friedel-Crafts Acylation

The corresponding resorcinol derivative **1a-1f** (1 eq) was dissolved by dropwise adding $BF_3 \cdot 2CH_3COOH$ (2.5 mL, 18.0 mmol). The resulting solution was stirred and refluxed for 3 to 4 h. After cooling the reaction mixture to room temperature 0.5 M aqueous KOAc (50 mL) was added dropwise and stirring was continued for further 30 minutes. The crude precipitate was filtered and recrystallized from $MeOH/H_2O$ (1:1, 60 mL) affording the 2,4-dihydroxyacetophenone analogs **3b-3f**. Selected compounds were additionally purified by column chromatography.

Reference compound: 1-(2,4-dihydroxy-5-methylphenyl)ethan-1-one (Ref-3b)

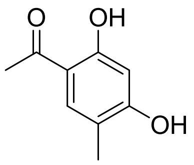

4-Methylresorcinol **1b** (400 mg, 3.23 mmol) was used to afford acetophenone **3b** as a pale yellow solid (374.6 mg, 2.26 mmol, 70%) after purification *via* flash chromatography (PE/EtOAc, 80:20). NMR data is in accordance with literature.[2] R_F = 0.4 ($CHCl_3$/MeOH, 90:10), m.p. 166-167 °C (lit. 167-168 °C).[2]

^{1}HNMR (300 MHz, Acetone-*d6*): δ [ppm] = 12.61 (s, 1H), 9.39 (s, 1H), 7.65 (d, J = 0.7 Hz, 1H), 6.35 (s, 1H), 2.54 (s, 3H), 2.15 (d, *J* = 0.4 Hz, 3H). ^{13}CNMR (75 MHz, Acetone-*d6*): δ [ppm] = 202.65, 163.47, 162.78, 133.19, 116.42, 113.07, 102.02, 25.35, 14.47; GC-MS (EI^+, 70 eV): *m/z* (%) = 166.0 [M^+] (42), 151.0 [$C_8H_7O_3^+$] (100).

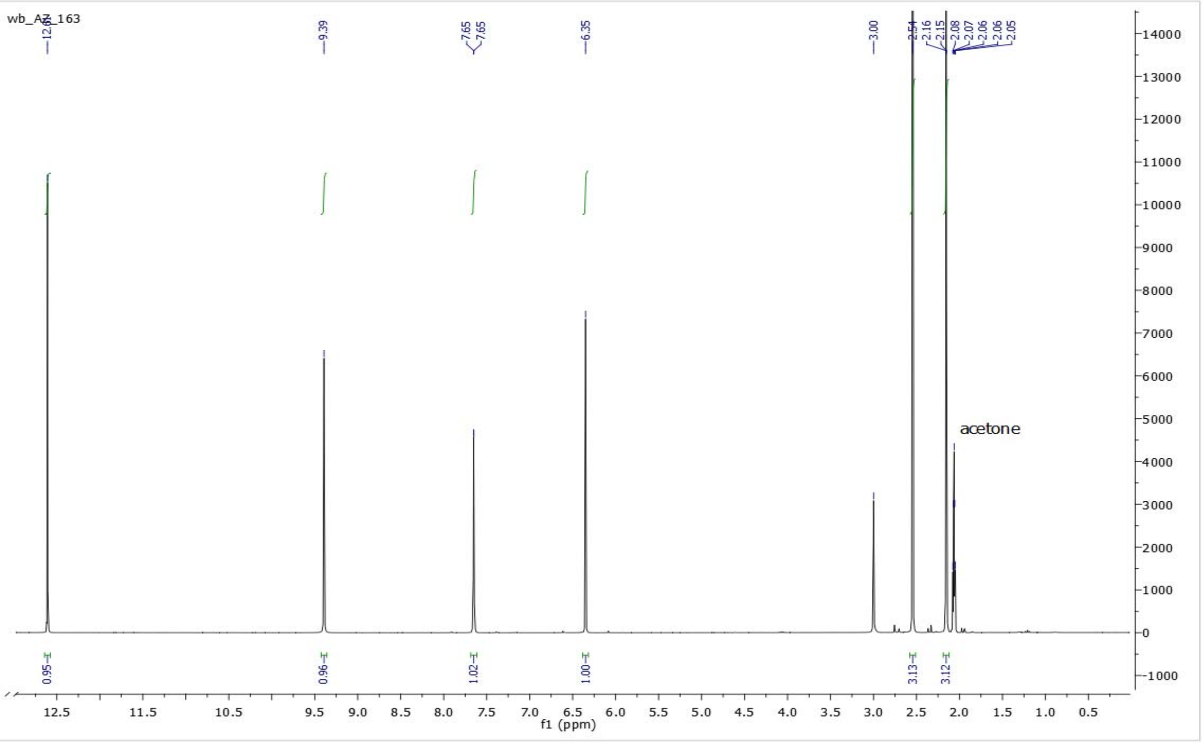

Figure S1. ^{1}HNMR of the reference compound **3b** obtained from chemical synthesis.

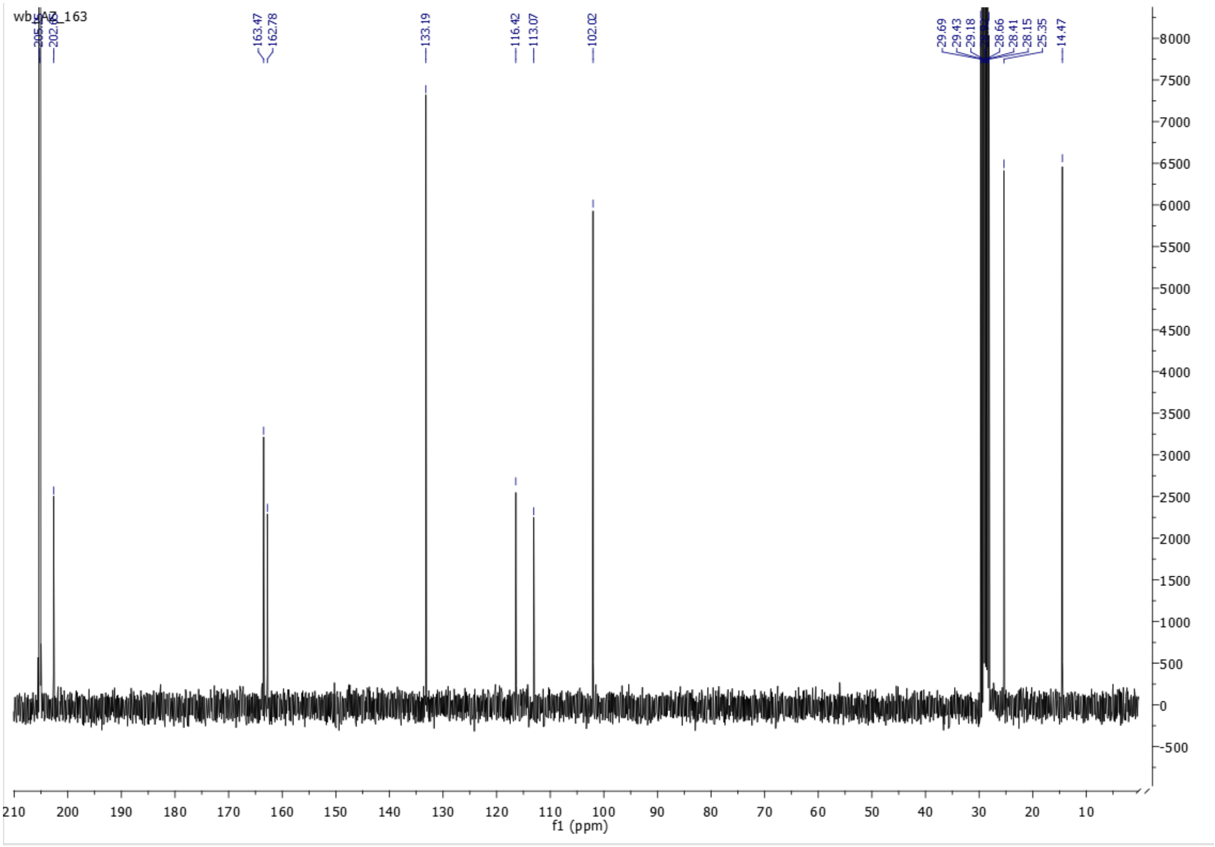

Figure S2. ^{13}CNMR of the reference compound **3b** obtained from chemical synthesis.

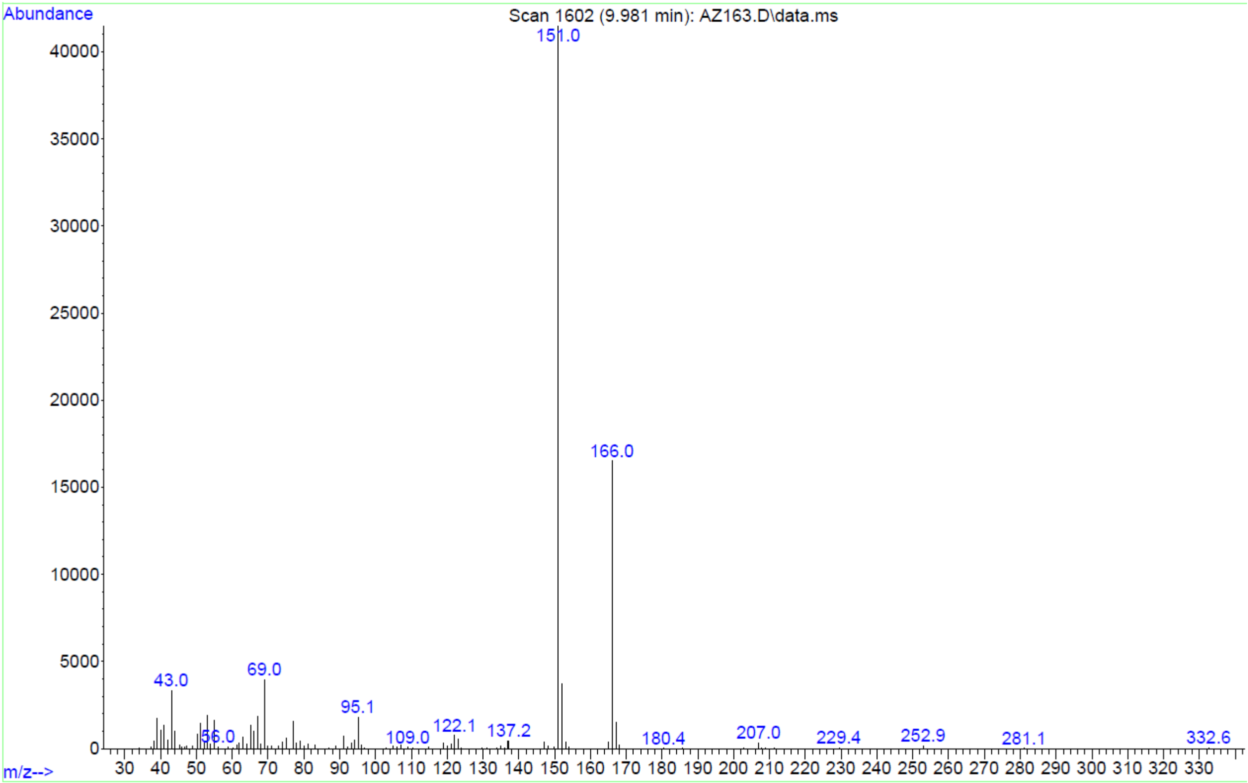

Figure S3. MS of the reference compound **3b** obtained from chemical synthesis.

Reference compound: 1-(5-ethyl-2,4-dihydroxyphenyl)ethan-1-one (Ref-3c)

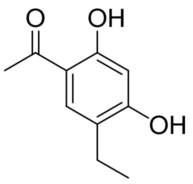

4-Ethylresorcinol **1c** (400 mg, 2.90 mmol) was used to afford acetophenone **3c** as a pale yellow solid (213.3 mg, 2.26 mmol, 41%) after purification *via* flash chromatography (PE/EtOAc, 90:10 to 60:40). R_F = 0.2 (PE/EtOAc, 90:10), m.p. 104-108°C (lit. 115-116 °C)[3]. NMR data is in accordance with literature.[4,6]

^{1}HNMR (300 MHz, Acetone-*d6*): δ [ppm] = 12.63 (s, 1H), 9.40 (s, 1H), 7.65 (s, 1H), 6.36 (s, 1H), 2.66 – 2.53 (m, 5H), 1.19 (t, *J* = 7.5 Hz, 3H). ^{13}CNMR (75 MHz, Acetone-*d6*): δ [ppm] = 202.71, 163.41, 162.48, 131.89, 122.73, 113.16, 102.24, 25.36, 22.35, 13.76; GC-MS (EI$^+$, 70 eV): *m/z* (%) = 180.1 [M$^+$] (33), 165.0 [$C_9H_9O_3^+$] (100).

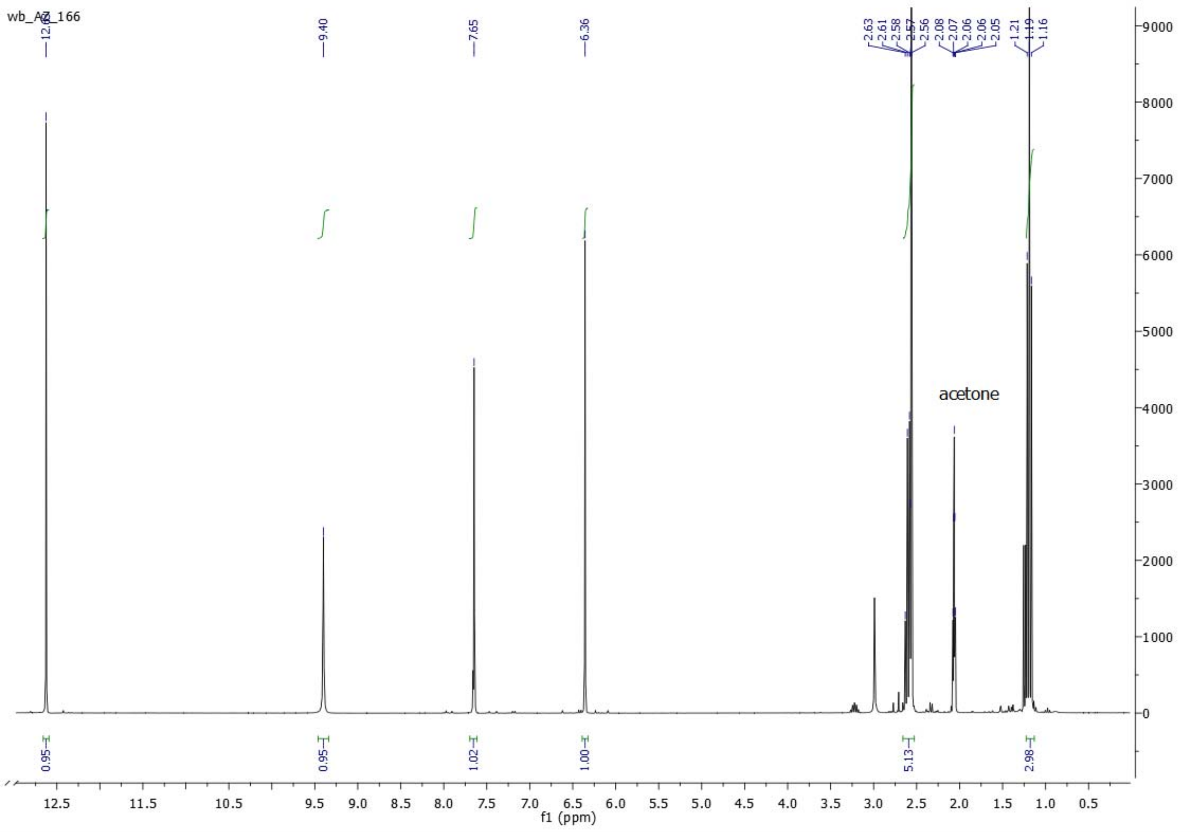

Figure S4. ^{1}HNMR of the reference compound **3c** obtained from chemical synthesis.

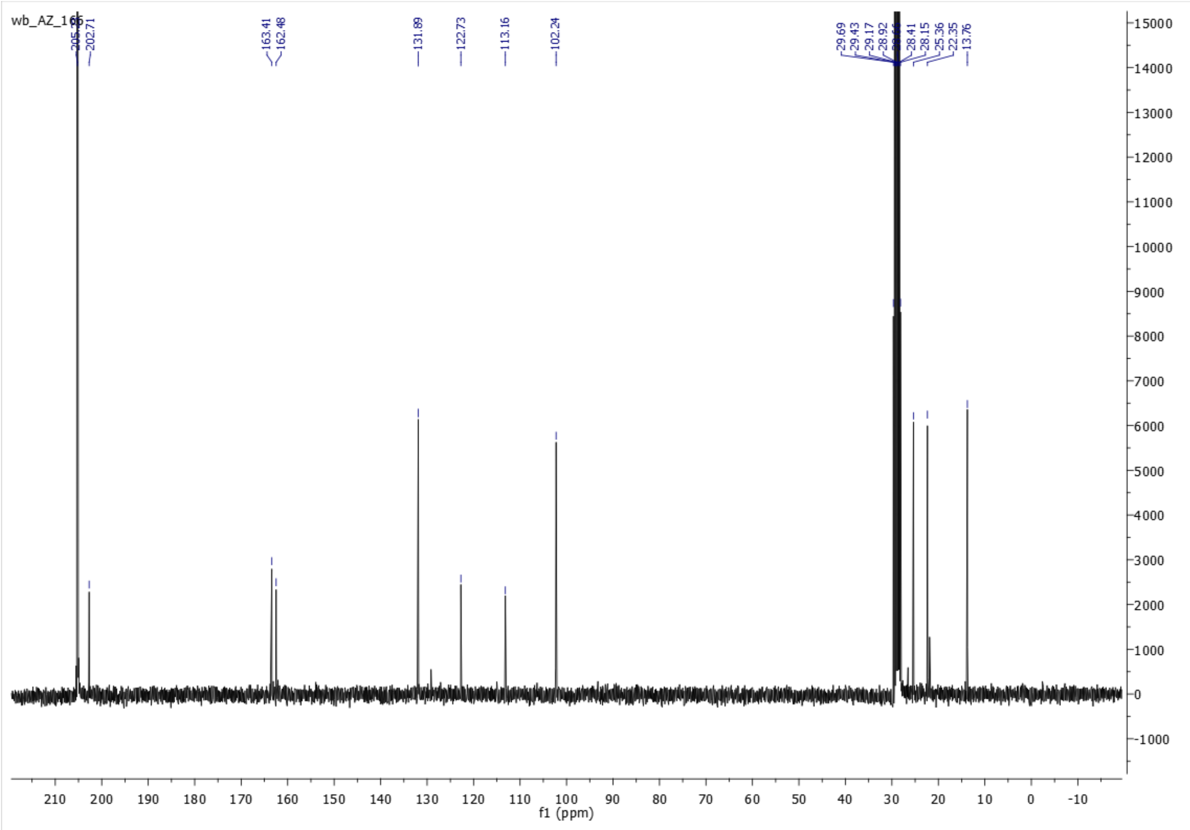

Figure S5. ^{13}CNMR of the reference compound **3c** obtained from chemical synthesis.

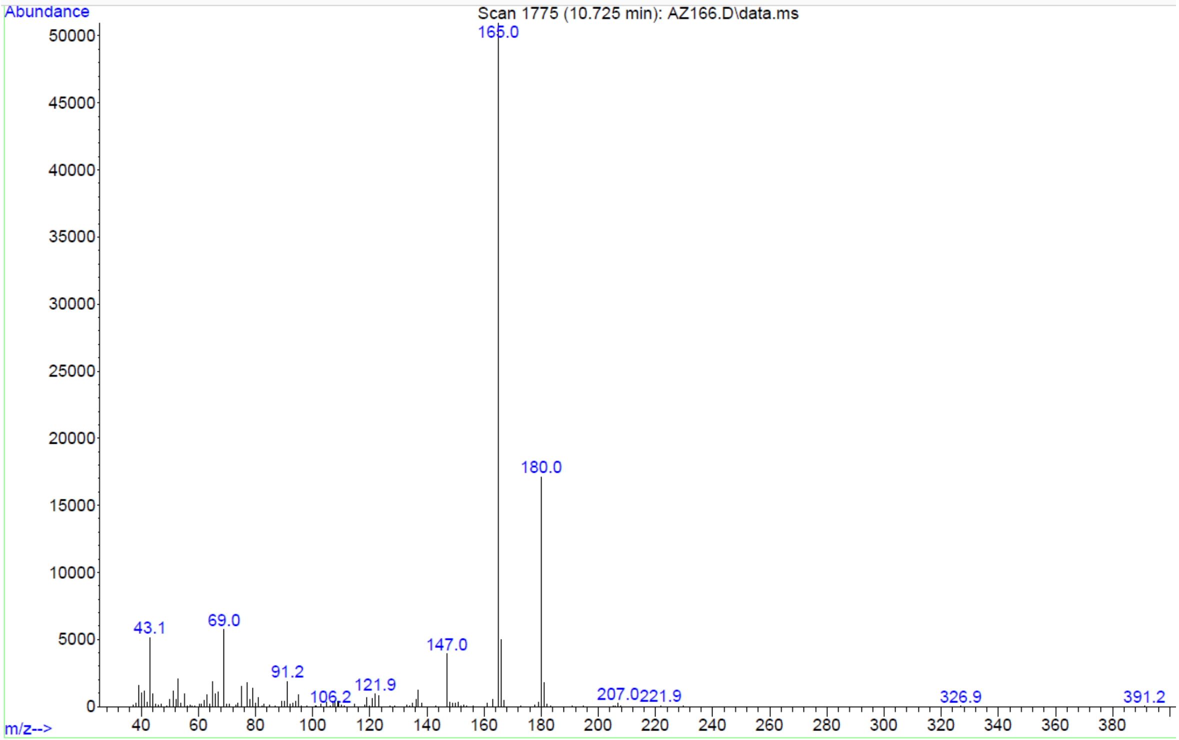

Figure S6. MS of the reference compound **3c** obtained from chemical synthesis.

Reference compound: 1-(5-butyl-2,4-dihydroxyphenyl)ethan-1-one (Ref-3d)

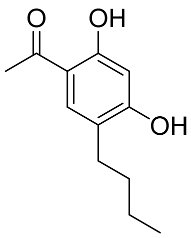

4-Butylresorcinol **1d** (400 mg, 2.41 mmol) was used to afford acetophenone **3d** as a pale yellow solid (359 mg, 1.73 mmol, 72%) after purification *via* flash chromatography (PE/EtOAc, 90:10 to 80:20). R_F = 0.5 (PE/EtOAc, 80:20), m.p. 82-84 °C (lit. 88 °C)[5]. NMR data is in accordance with literature.[6]

^{1}HNMR (300 MHz, Acetone-*d6*): δ [ppm] = 12.61 (s, 1H), 9.37 (s, 1H), 7.64 (s, 1H), 6.35 (s, 1H), 2.64 – 2.49 (m, 5H), 1.58 (tt, *J* = 7.5, 6.6 Hz, 2H), 1.38 (dq, *J* = 14.3, 7.2 Hz, 2H), 0.93 (dd, *J* = 9.9, 4.7 Hz, 3H). ^{13}CNMR (75 MHz, Acetone-*d6*): δ [ppm] = 202.71, 163.35, 162.56, 132.67, 121.33, 113.13, 102.26, 32.05, 28.96, 25.36, 22.26, 13.34; GC-MS (EI^+, 70 eV): *m/z* (%) = 208.1 [M^+] (25), 193.1 [$C_{11}H_{13}O_3^+$] (11), 165.0 [$C_9H_9O_3^+$] (100).

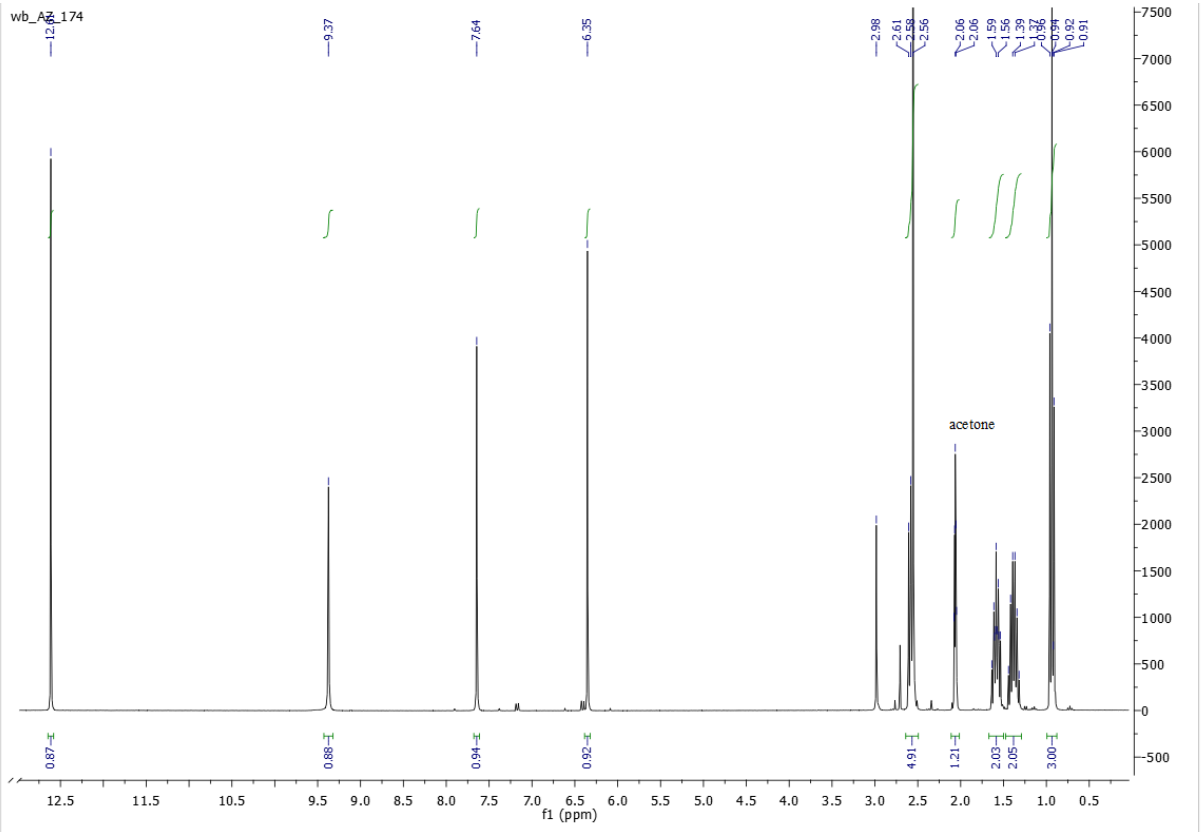

Figure S7. ^{1}HNMR of the reference compound **3d** obtained from chemical synthesis.

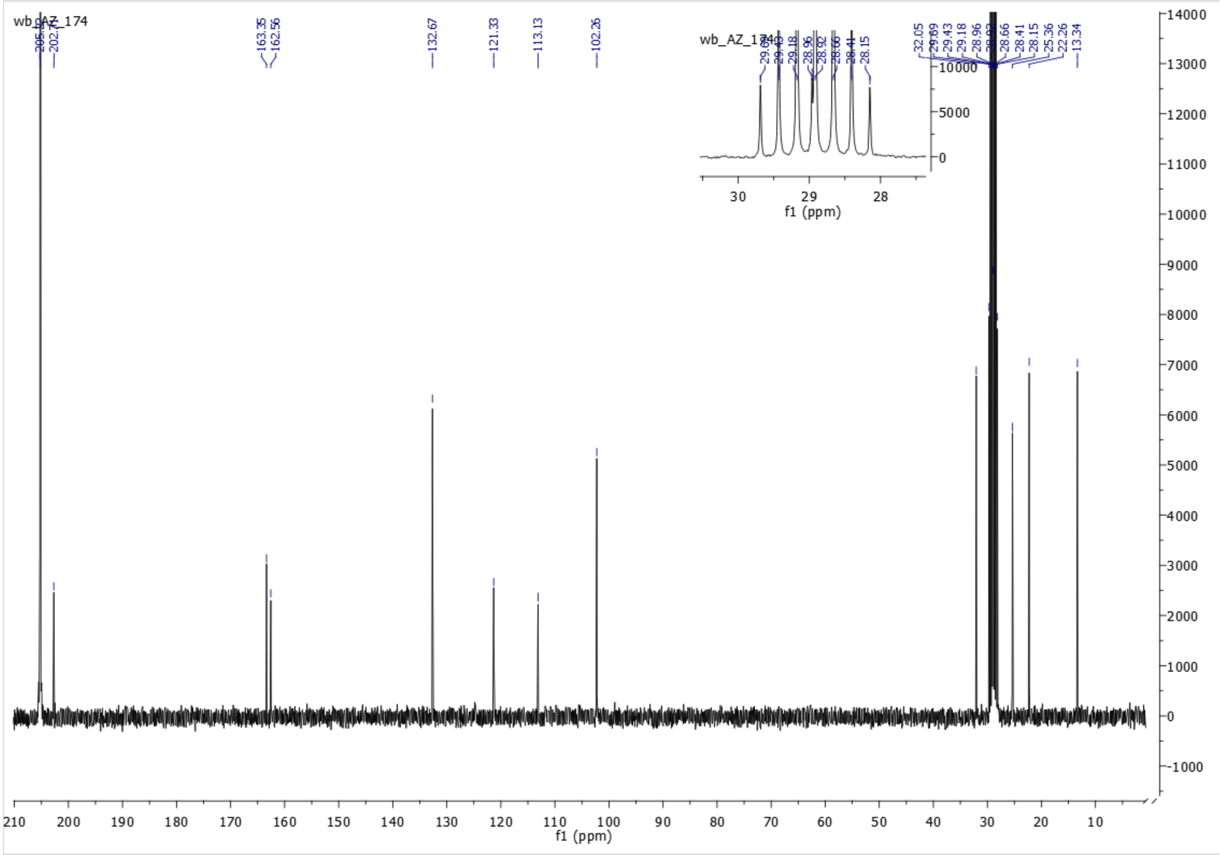

Figure S8. ^{13}CNMR of the reference compound **3d** obtained from chemical synthesis.

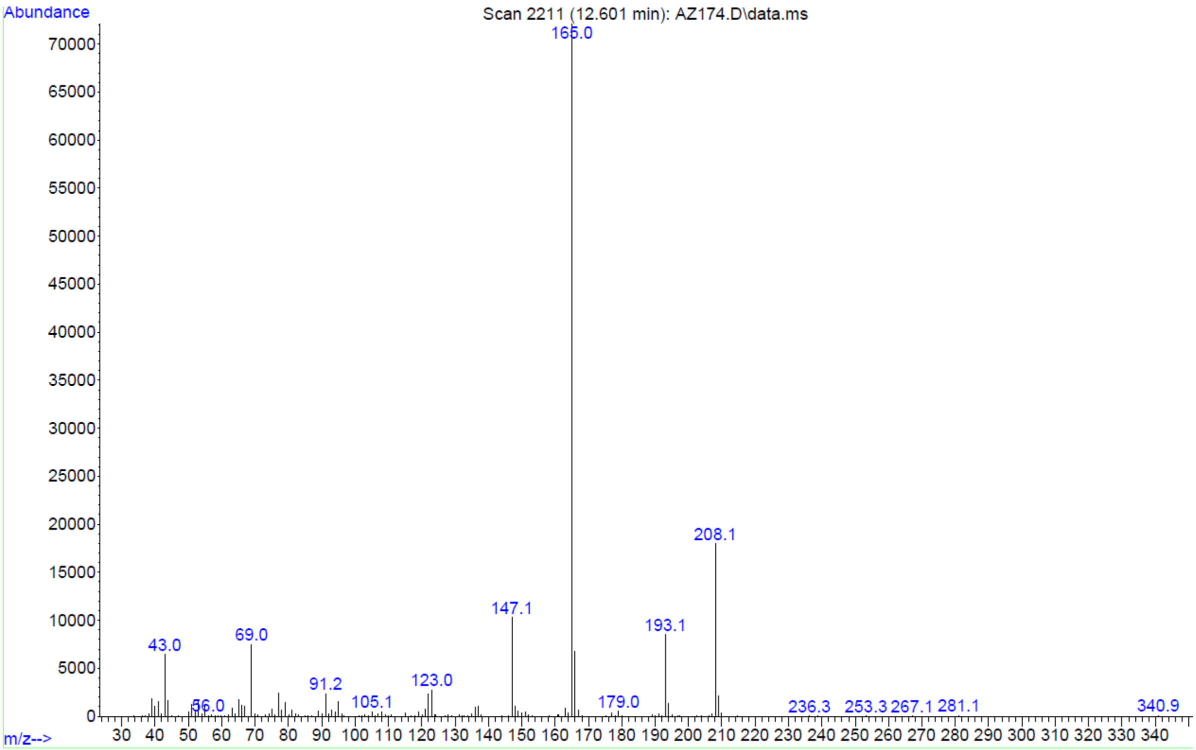

Figure S9. MS of the reference compound **3d** obtained from chemical synthesis.

Reference compound: 1-(5-hexyl-2,4-dihydroxyphenyl)ethan-1-one (Ref-3e)

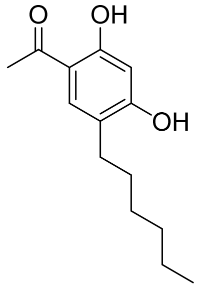

4-Hexylresorcinol **1e** (400 mg, 2.06 mmol) was used to afford acetophenone **3e** as a white solid (246 mg, 1.04 mmol, 51%) after purification *via* flash chromatography (PE/EtOAc, 95:5 to 80:20). R_F = 0.6 (PE/EtOAc, 80:20) m.p. 86-87 °C (lit.: 87 °C)[5]. NMR data is in accordance with literature.[6]

^{1}HNMR (300 MHz, $CDCl_3$): δ [ppm] = 12.58 (s, 1H), 7.46 (s, 1H), 6.35 (s, 1H), 2.58 (s, 1H), 2.55 (m, 2H), 1.72 – 1.52 (m, 2H), 1.45 – 1.28 (m, 2H), 0.91 (t, *J* = 6.9 Hz, 3H). ^{13}CNMR (75 MHz, $CDCl_3$): δ [ppm] = 202.87, 163.10, 161.19, 132.41, 121.22, 113.93, 103.17, 31.70, 29.80, 29.42, 29.09, 26.21, 22.62, 14.08; GC-MS (EI^+, 70 eV): *m/z* (%) = 236.1 [M^+] (17), 221.1 [$C_{13}H_{17}O_3^+$] (7), 165.0 [$C_9H_9O_3^+$] (100).

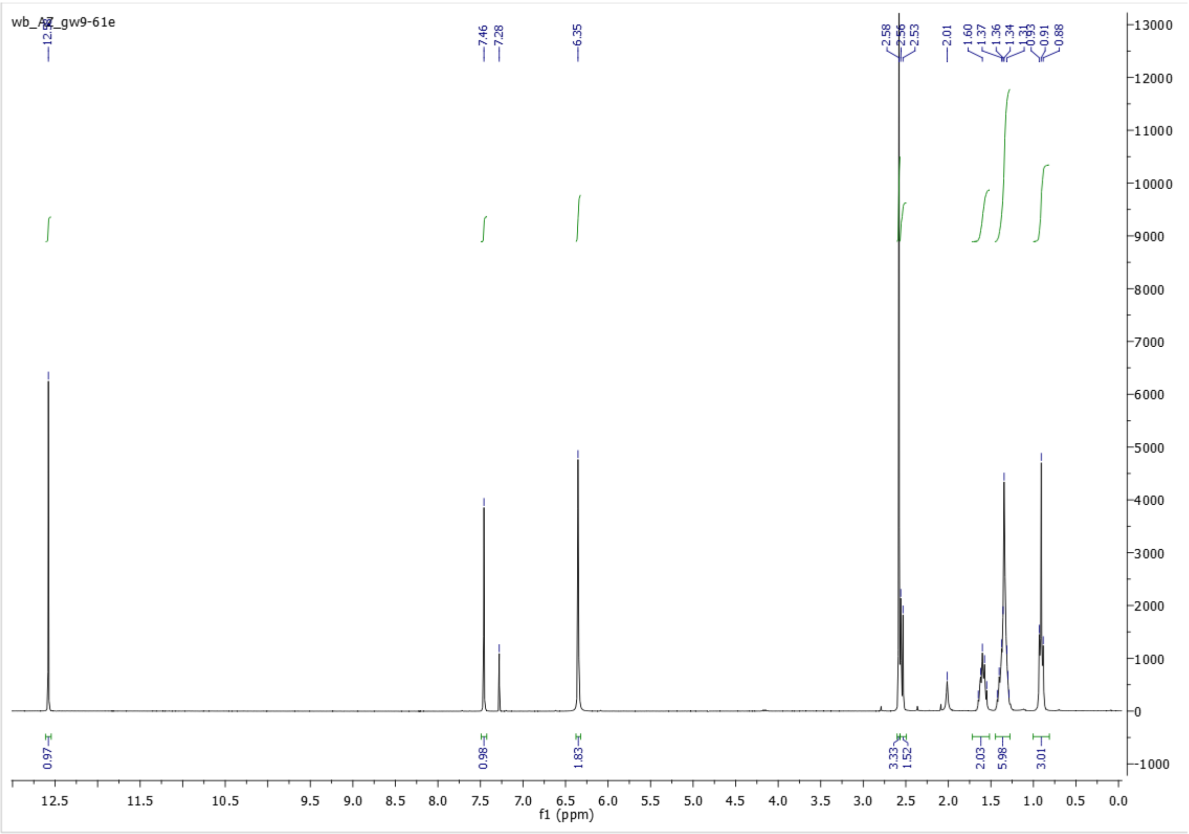

Figure S10. ^{1}HNMR of the reference compound **3e** obtained from chemical synthesis.

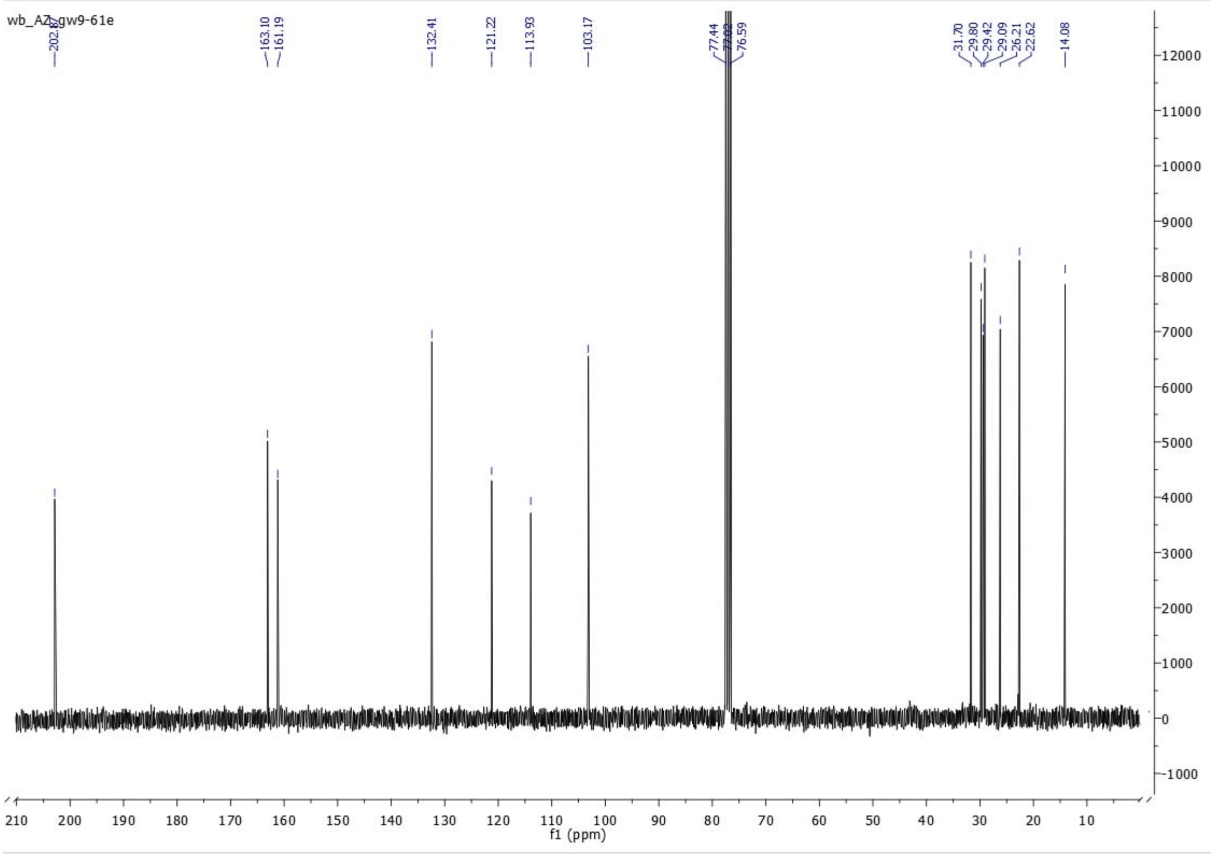

Figure S11. ^{13}CNMR of the reference compound **3e** obtained from chemical synthesis.

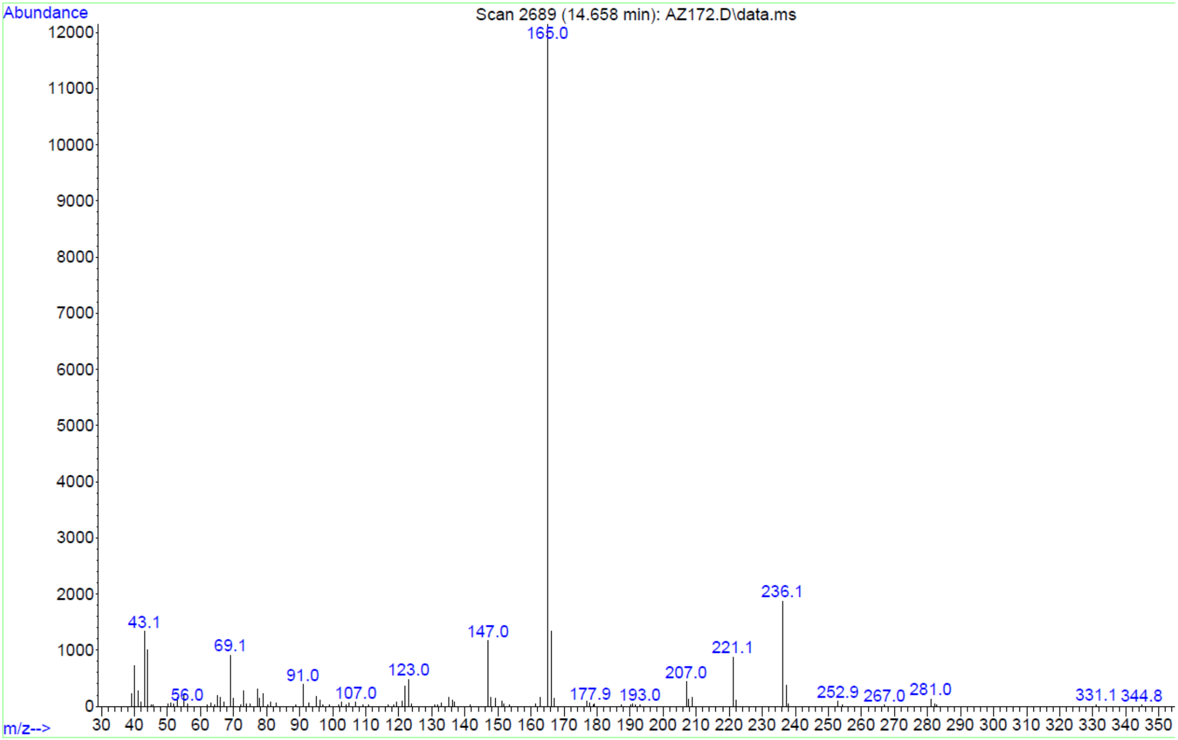

Figure S12. MS of the reference compound **3e** obtained from chemical synthesis.

Reference compound: 1-(5-chloro-2,4-dihydroxyphenyl)ethan-1-one (Ref-3f)

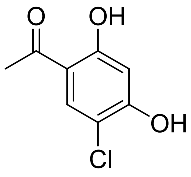

4-Chlororesorcinol **1f** (500 mg, 3.47 mmol) was used to afford acetophenone **3f** as a pink solid (614 mg, 3.30 mmol, 95 %). R_F = 0.9 ($CHCl_3$/MeOH, 90:10), m.p. 173-175 °C (lit.: 165-167 °C)[4]. ^{1}H-NMR and MS-data are in accordance with literature.[4]

^{1}HNMR (300 MHz, Acetone-*d6*) δ 12.59 (s, 1H), 9.94 (s, 1H), 7.91 (s, 1H), 6.51 (s, 1H), 2.62 (s, 3H); ^{13}CNMR (75 MHz, Acetone-*d6*) δ 202.72, 163.37, 159.67, 132.54, 113.96, 111.50, 103.85, 25.56; GC-MS (EI^+, 70 eV): *m/z* (%) = 186.0 [M^+] (38), 171.0 [$C_7H_4ClO_3^+$] (100).

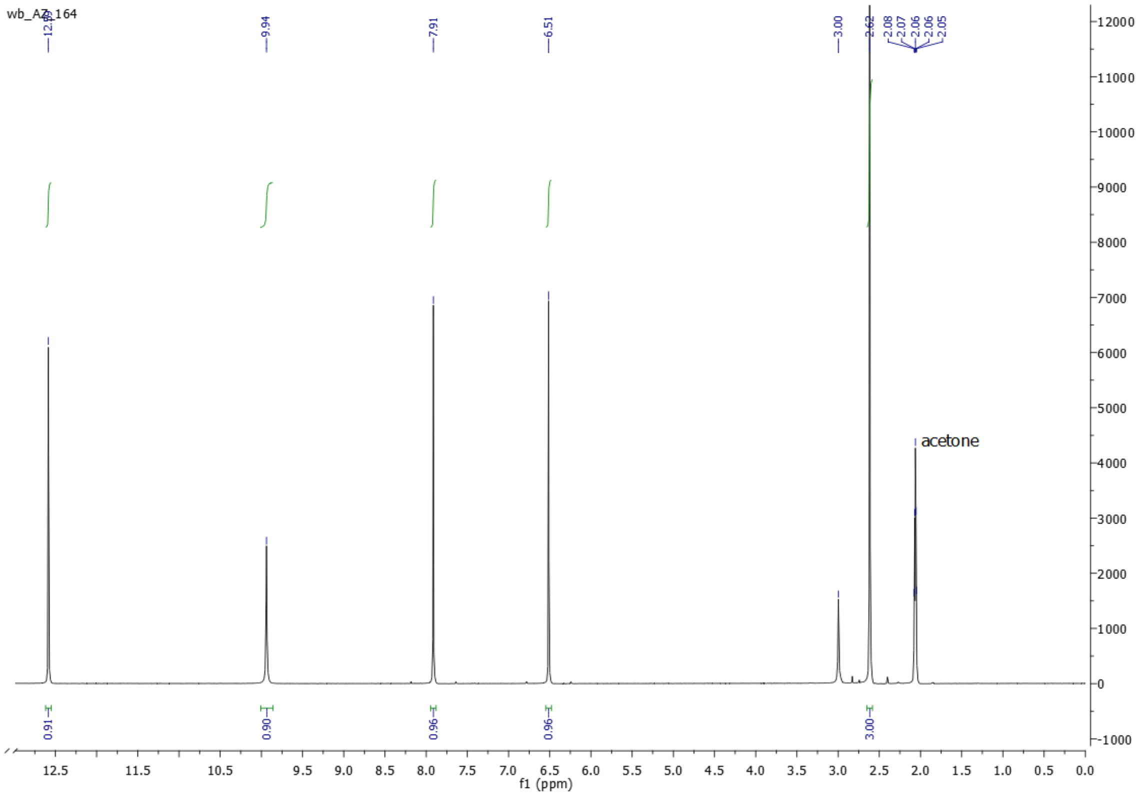

Figure S13. ^{1}HNMR of the reference compound **3f** obtained from chemical synthesis.

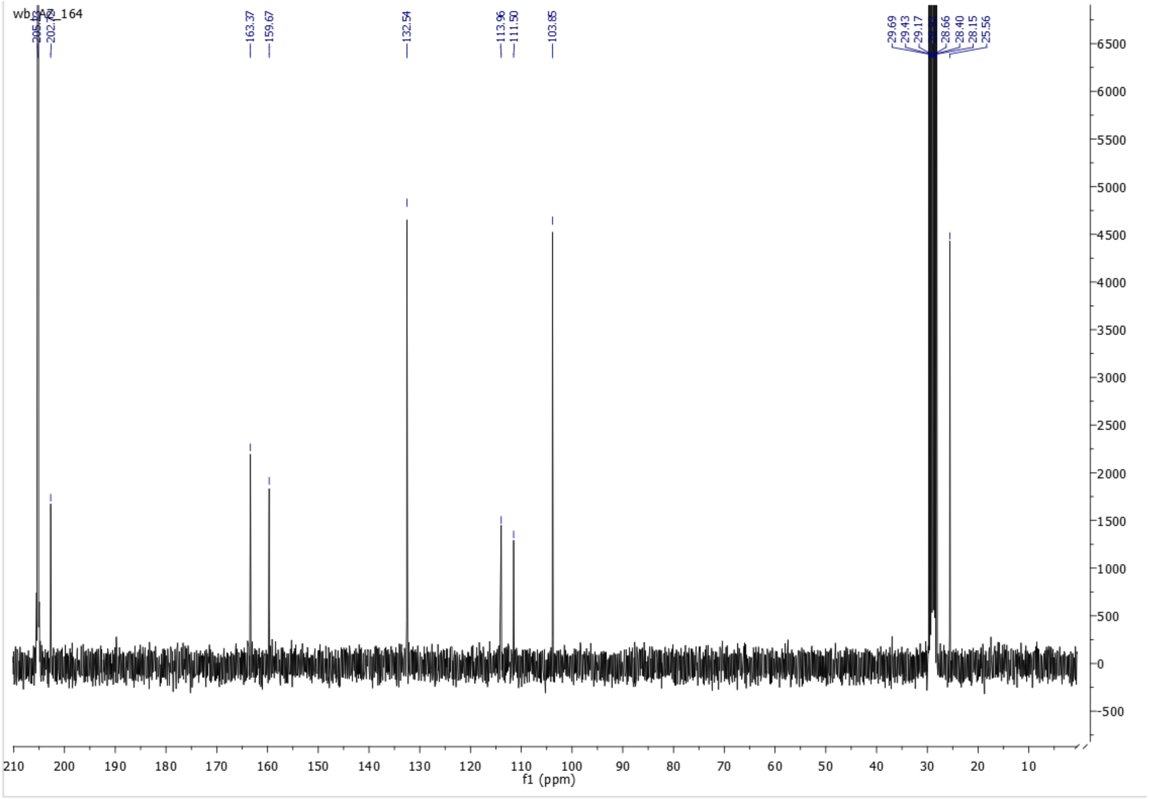

Figure S14. ^{13}CNMR of the reference compound **3f** obtained from chemical synthesis.

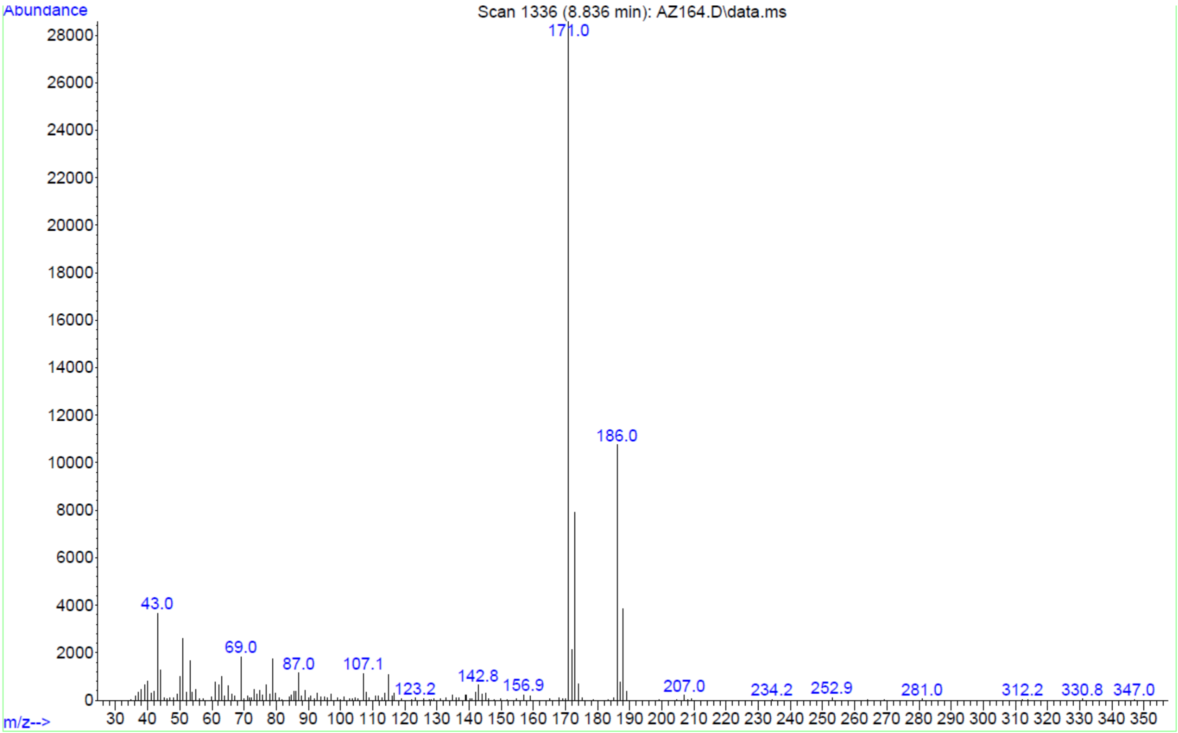

Figure S15. MS of the reference compound **3f** obtained from chemical synthesis.

Activity Assay

ATase-batch activities were measured on a Thermo Scientific Genesys 10 UV Scanning UV/Vis spectrophotometer according to a modified procedure from literature.[3] When following the disproportionation of MAPG into DAPG and PG spectrophotometrically, an increase of absorption is recorded due to the formation of DAPG (ε = 20 mM^{-1} cm^{-1}, λ = 370 nm). One unit of activity was defined as the µmol of product formed by an enzyme in 1 min per 1 milligram of protein under the following conditions: potassium phosphate buffer (960 µL, 100 mM, pH 7.5) and MAPG (1.2 µmol, 30 µL of a 40 mM stock solution prepared in DMSO) were added to a cuvette and preheated to 35 °C. The reaction (1 mL total volume, 3 vol% DMSO) was started by the addition of the enzyme-containing cell-free extract (10 µL ≡ 1.43 mg wet cells). The reaction was followed for 1 minute. All reactions were performed as a duplicate. The protein concentration (Bradford) was measured [(ε = 0.083 mL mg^{-1} cm^{-1}, λ = 595 nm)] and specific activities were determined as units per mg protein.

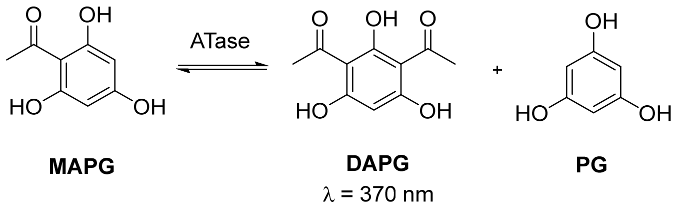

Figure S16. Reaction scheme of assay.

Screening Procedure

Resorcinol derivative **1a-1h** or aniline derivative **1i-1j** (0.01 mmol, 10 mM final concentration) was suspended in potassium phosphate buffer (100 mM, pH 7.5). Then, cell-free extract of recombinant ATase (0.066 U) was added to the reaction mixture. The bioacylation was started by addition of the donor **2a-2d** (0.1 mmol, 100 mM final concentration) and amine additive (imidazole or DABCO, 100 mM final concentration, added from 1M stock solution prepared in the reaction buffer). The reaction mixture was shaken for 18-24 h at 35˚C and 750 rpm in an orbital shaker. Reaction was quenched by addition of acetonitrile (1 mL). The precipitated protein was removed by centrifugation (30 min, 14,000 rpm) and the supernatant was subjected to HPLC for determination of conversions. As a negative control, reactions without enzyme were performed.

Semi-preparative Scale Friedel-Crafts Bioacylation

Resorcinol derivative **1a**-**1f** or aniline derivative **1i**-**1j** (0.25 mmol, 10 mM final concentration) was dissolved in potassium phosphate buffer (100 mM, pH 7.5) in a shaking flask. Cell-free extract containing the *Pp*ATaseCH (2.5 mL, 1.65 U) was added to the reaction mixture followed by ethyl thioacetate (266 μL, 2.5 mmol, 100 mM final concentration) addition. For substrates **1c**-**1f**, imidazole (2.5 mmol, 100 mM final concentration, added from a 1 M stock solution prepared in the reaction buffer) was added. The bioacetylation (25 mL total volume) was run at 35 °C and 120 rpm for 24 h. The resulting suspension was extracted with ethyl acetate (2 × 20 mL), centrifuged (5 min, 4,000 rpm). Then organic layers were separated, combined and dried over anhydrous $MgSO_4$. Solvent was removed under reduced pressure and crude product was purified by column chromatography using silica gel, DCM:MeOH or cyclohexane:EtOAc as an eluent. Compounds were characterized by ^{1}HNMR and ^{13}CNMR and GC-MS and the chemical identity was confirmed by comparison to literature. For compounds **3e** and **3f** a mixture of C-acylated and O-acylated products was observed. To remove O-acylated products, mixture was submitted to hydrolysis with 1 N NaOH for 1h at rt, then acidified with 1 N HCl, extracted with EtOAc and purified by column chromatography.

1-(2,4-dihydroxyphenyl)ethan-1-one (3a)

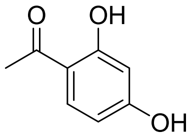

1-(2,4-dihydroxyphenyl)ethan-1-one was obtained from semi-preparative biotransformation as a white solid with 88% product yield (33.6 mg, 0.221 mmol) after purification *via* column chromatography (silica gel, *c*-hex/EtOAc, 100:0 to 80:20). R_F = 0.6 (c-hex/EtOAc, 1:1), m.p. 173-175 °C (lit.: 165-167°C)[4]. ^{1}HNMR (300 MHz, DMSO-*d6*): δ [ppm] = 12.61 (s, 1H), 10.63 (s, 1H), 7.75 (d, *J* = 8.8 Hz, 1H), 6.38 (dd, *J* = 8.8, 2.4 Hz, 1H), 6.24 (d, *J* = 2.3 Hz, 1H), 2.52 (s, 3H); ^{13}CNMR (75 MHz, DMSO-*d6*): δ [ppm] = 203.17, 165.34, 164.66, 134.18, 113.31, 108.57, 102.74, 26.82; GC-MS (EI^+, 70 eV): m/z (%) = 152.1 [M^+] (47), 137.0 [$C_7H_5O_3^+$] (100), 109 [$C_6H_5O_2^+$] (3).

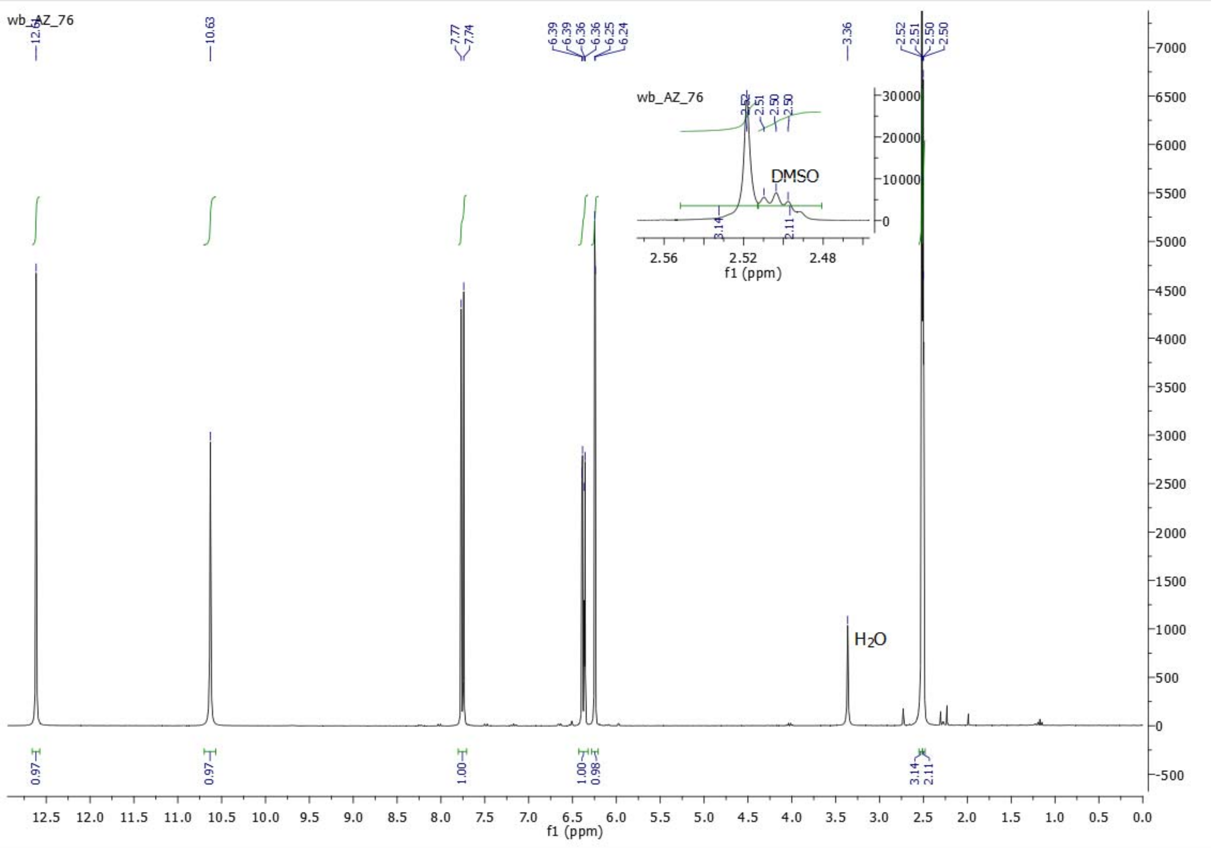

Figure S17. ^{1}HNMR of compound **3a** isolated from the preparative scale bioacylation.

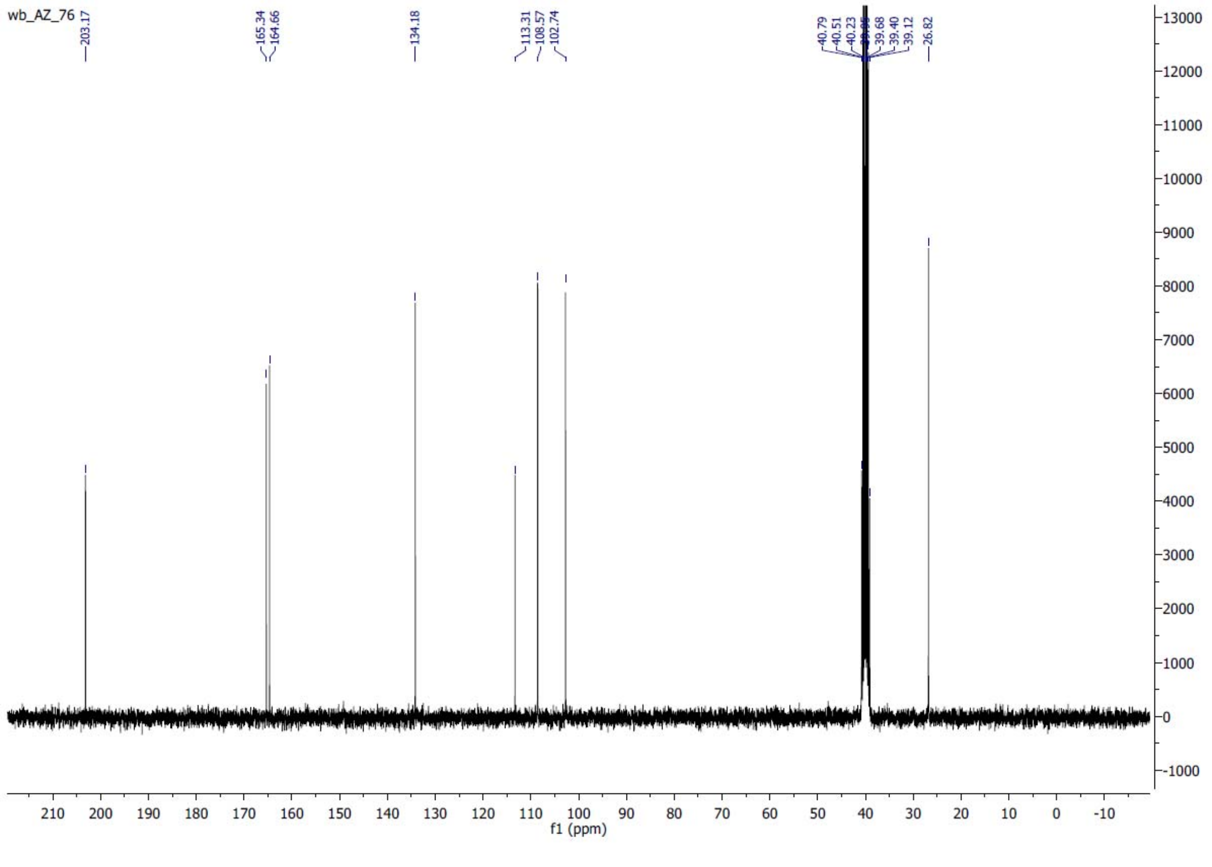

Figure S18. ^{13}CNMR of compound **3a** isolated from the preparative scale bioacylation.

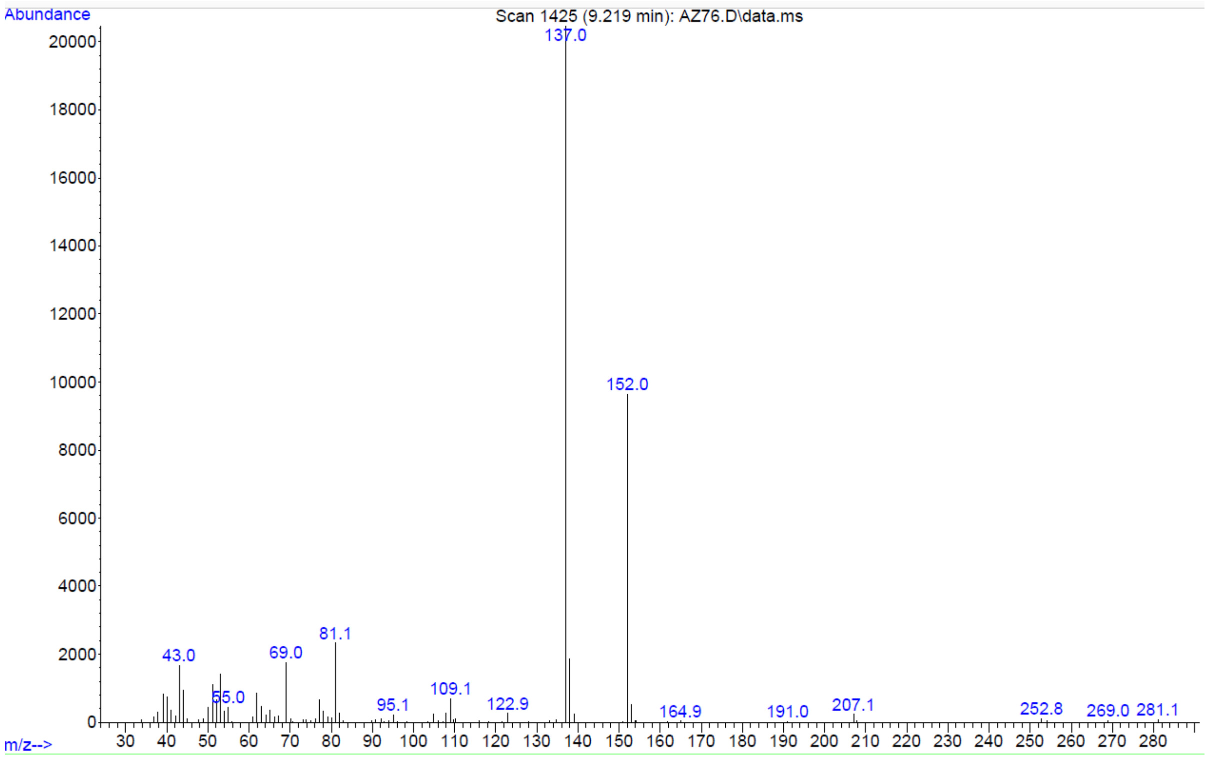

Figure S19. MS of compound **3a** isolated from the preparative scale bioacylation.

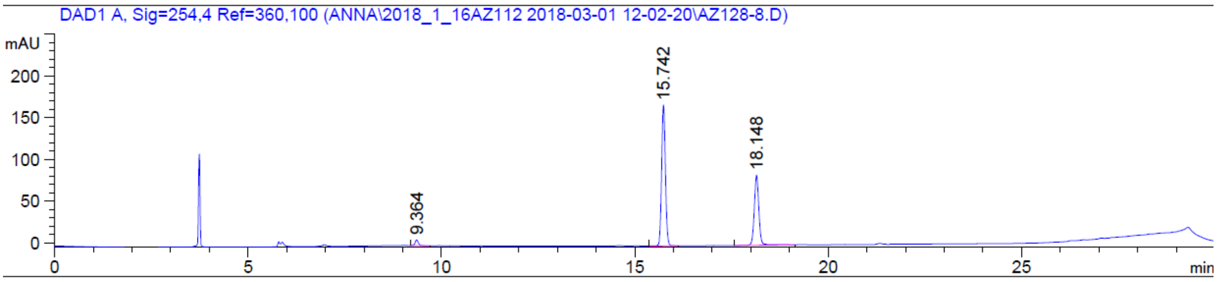

Figure S20. HPLC-chromatogram showing analytical-scale C-acylation of **1a** (t_r = 9.4 min) into **3a** (t_r = 15.7 min) using ethyl thioacetate (**2a**, t_r = 18.1 min) as an acyl donor.

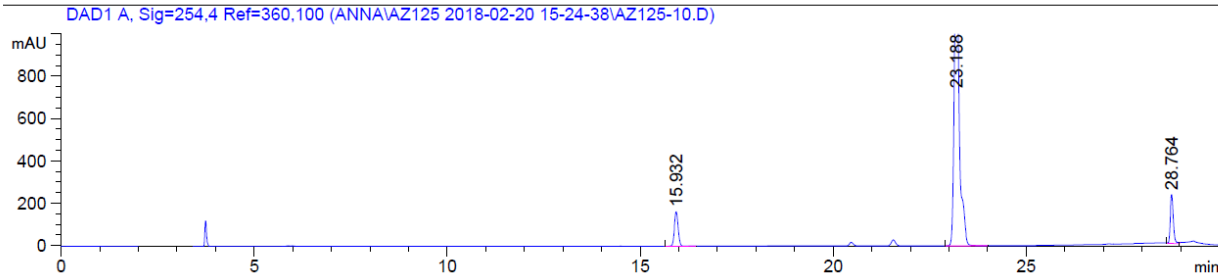

Figure S21. HPLC-chromatogram showing analytical-scale C-acylation of **1a** (t_r = 9.4 min) into **3a** (t_r = 15.9 min) using *S*-Phenyl thioacetate (**2b**, t_r = 23.2 min) as an acyl donor.

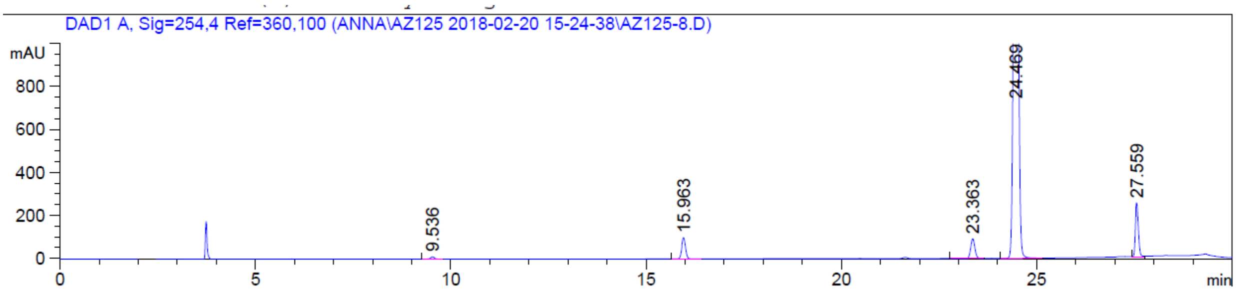

Figure S22. HPLC-chromatogram showing analytical-scale C-acylation of **1a** (t_r = 9.5 min) into **3a** (t_r = 15.9 min) using *S*-4-Nitrobenzyl thioacetate (**2c**, t_r = 24.5 min) as an acyl donor.

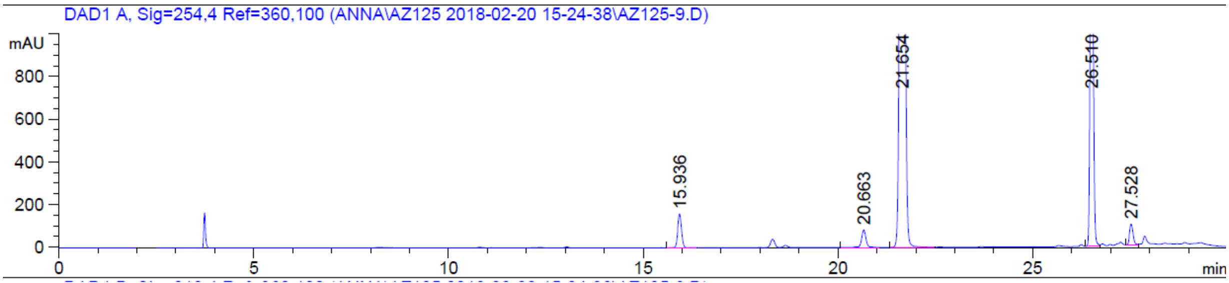

Figure S23. HPLC-chromatogram showing analytical-scale C-acylation of **1a** (t_r = 9.5 min) into **3a** (t_r = 15.9 min) using 2-acetylthioacetophenone (**2d**, t_r = 21.7 min) as an acyl donor.

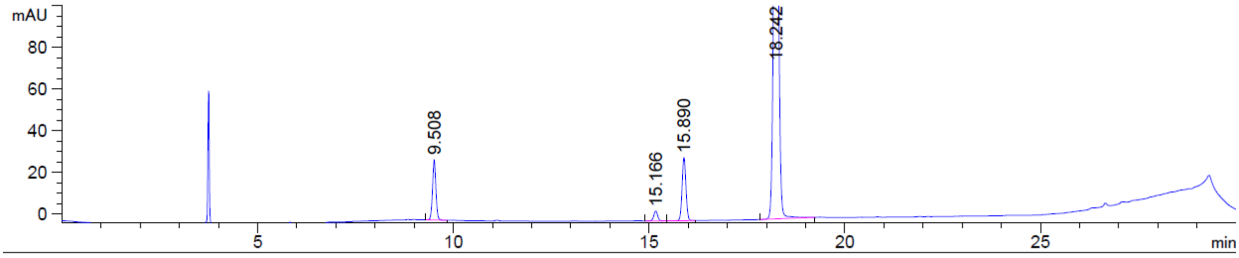

Figure S24. HPLC-chromatogram showing analytical-scale C-acylation of **1a** (t_r = 9.5 min) into C-acylated **3a** (t_r = 15.9 min) and O-acylated **4a** (t_r = 15.2 min) using ethyl thioacetate (**2a**, t_r = 18.2 min) as an acyl donor after 15 min.

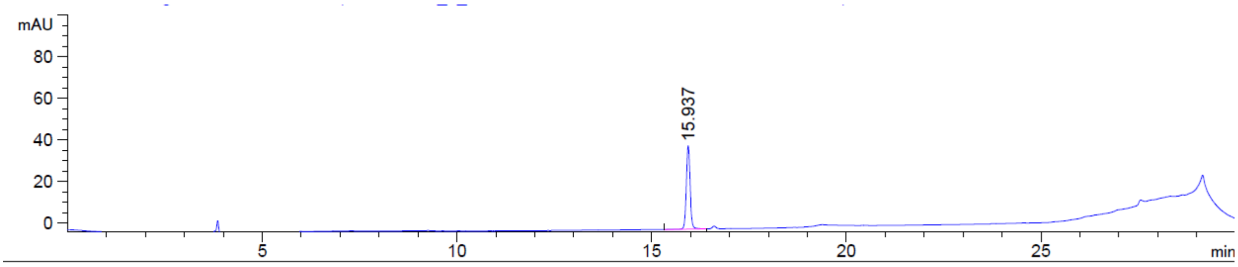

Figure S25. HPLC-chromatogram showing reference compound **3a** (*tr* = 15.9 min).

1-(2,4-dihydroxy-5-methylphenyl)ethan-1-one (3b)

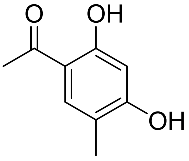

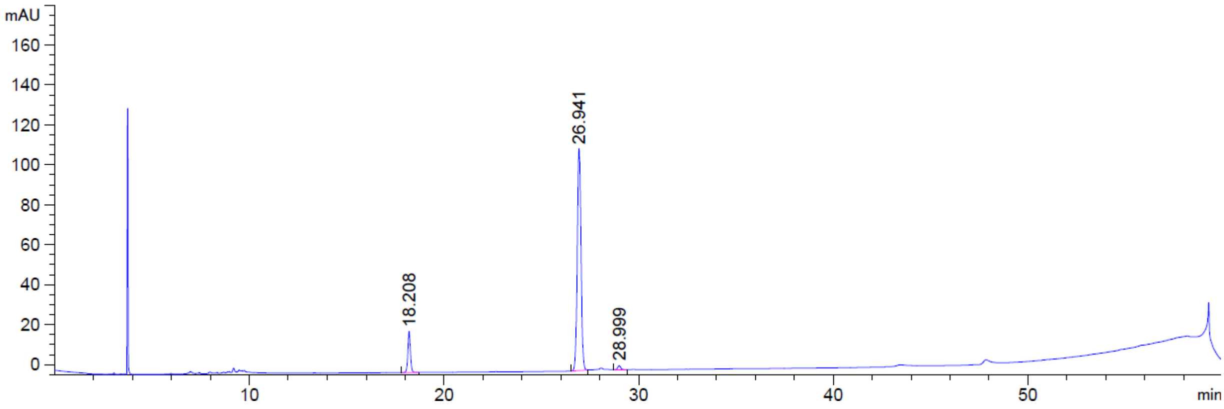

Figure S26. HPLC-chromatogram showing analytical-scale C-acylation of **1b** (t_r = 18.2 min) into **3b** (t_r = 29.0 min) using ethyl thioacetate (**2a**, t_r = 26.9 min) as an acyl donor.

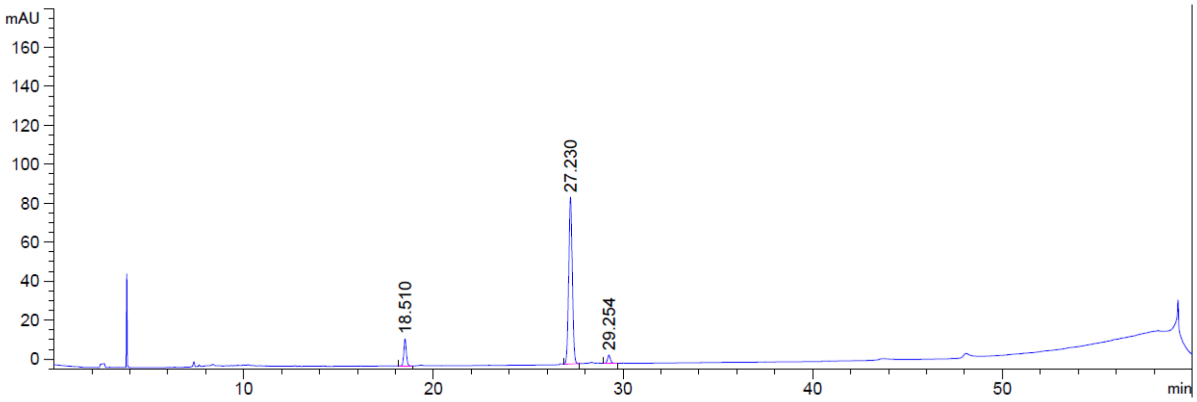

Figure S27. HPLC-chromatogram showing analytical-scale C-acylation of **1b** (*tr* = 18.5 min) into **3b** (t_r = 29.3 min) using ethyl thioacetate (**2a**, t_r = 27.2 min) as an acyl donor with imidazole addition (100 mM).

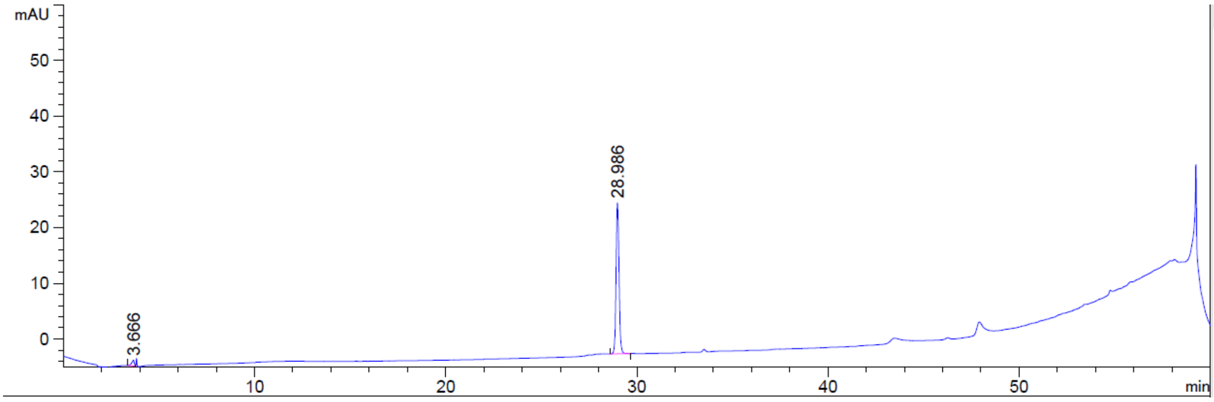

Figure S28. HPLC-chromatogram showing reference compound **3b** (*tr* = 29.0 min).

1-(5-ethyl-2,4-dihydroxyphenyl)ethan-1-one (**3c**)

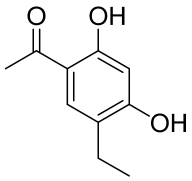

1-(5-ethyl-2,4-dihydroxyphenyl)ethan-1-one was obtained from semi-preparative biotransformation as a pale yellow solid with 49% product yield (22 mg, 0.122 mmol) after purification *via* column chromatography (silica gel, *c*-hex/EtOAc, 100:0 to 80:20), m.p. 101-109 °C.

^{1}HNMR (300 MHz, $CDCl_3$): δ_C [ppm] = 12.62 (s, 1H), 7.47 (s, 1H), 6.35 (s, 1H), 2.65 – 2.54 (m, 5H), 1.24 (t, *J* = 7.5 Hz, 3H). ^{13}CNMR (75 MHz, $CDCl_3$): δ_C [ppm] = 202.91, 163.10, 161.25, 131.58, 122.64, 113.88, 103.10, 77.44, 77.02, 76.59, 26.18, 22.47, 14.07. GC-MS (EI$^+$, 70 eV): *m/z* (%) = 180.1 [M^+] (36), 165.0 [$C_9H_9O_3^+$] (100).

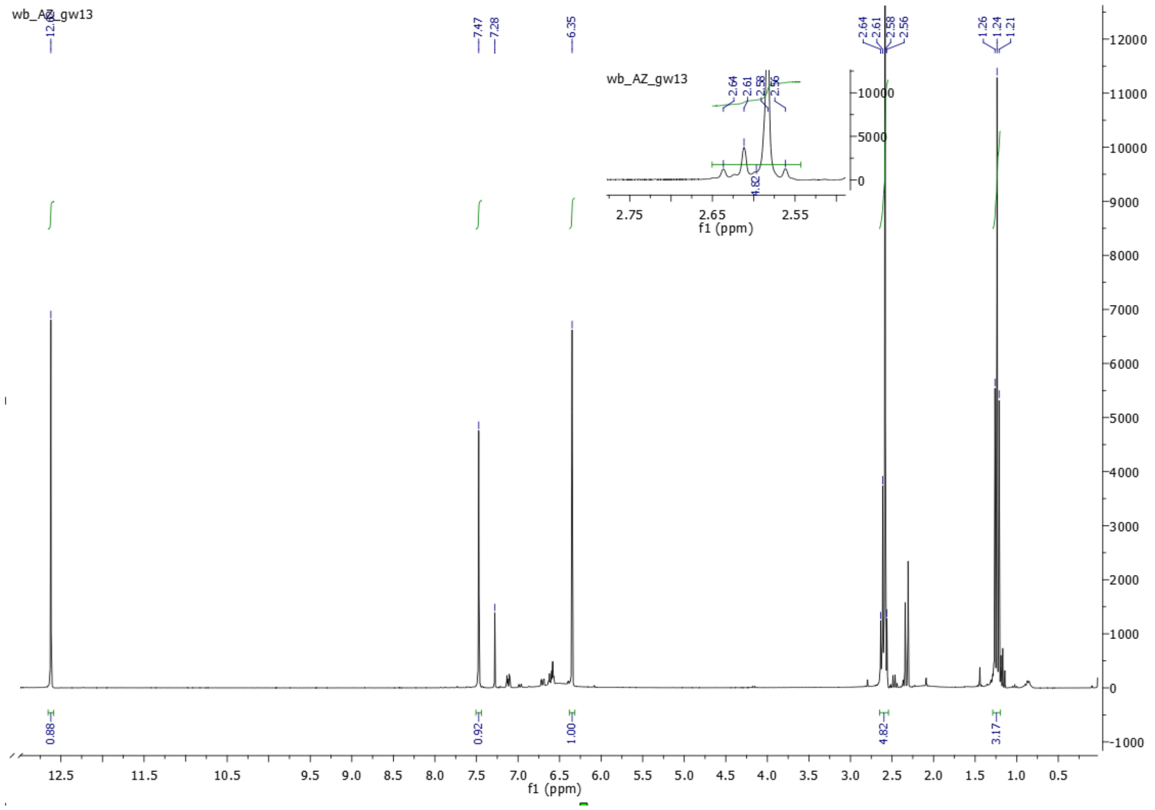

Figure S29. ^{1}HNMR of compound **3c** isolated from the preparative scale bioacylation.

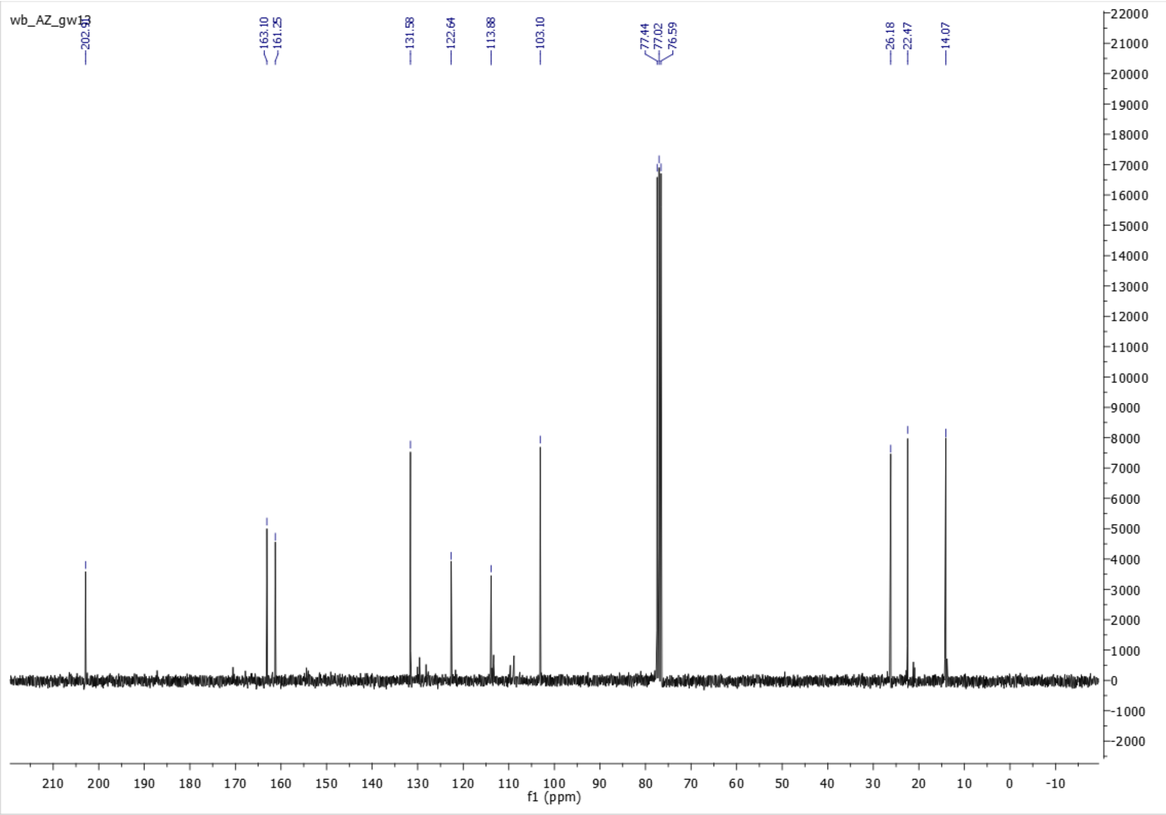

Figure S30. ^{13}CNMR of compound **3c** isolated from the preparative scale bioacylation.

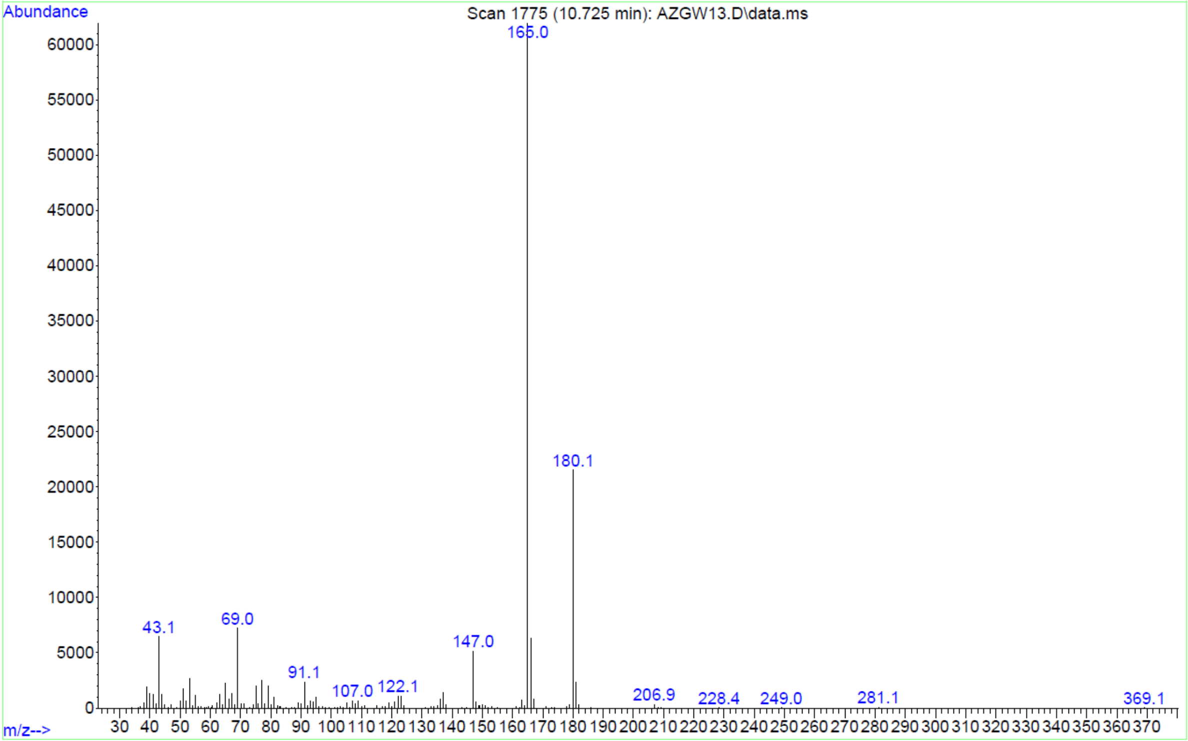

Figure S31. MS of compound **3c** isolated from the preparative scale bioacylation.

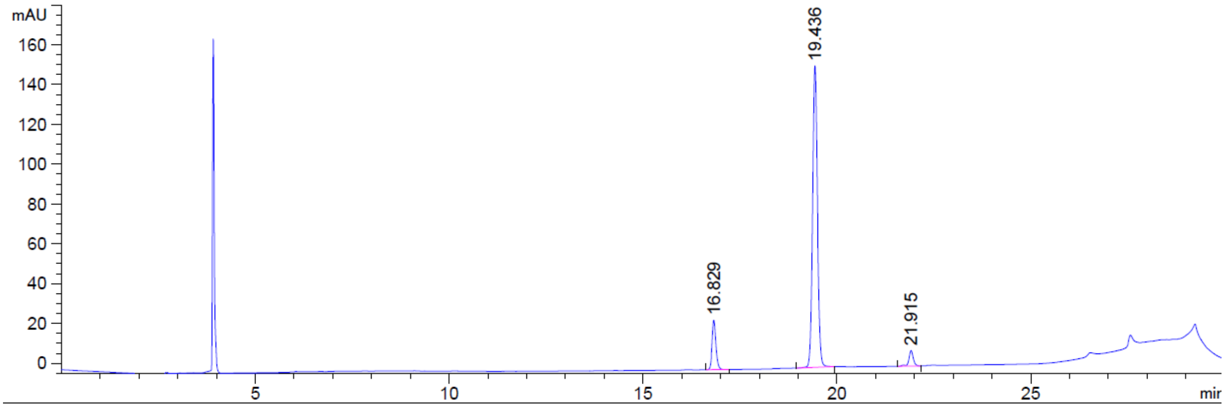

Figure S32. HPLC-chromatogram showing analytical-scale C-acylation of **1c** (*tr* = 16.8 min) into **3c** (t_r = 21.9 min) using ethyl thioacetate (**2a**, t_r = 19.4 min) as an acyl donor without imidazole addition.

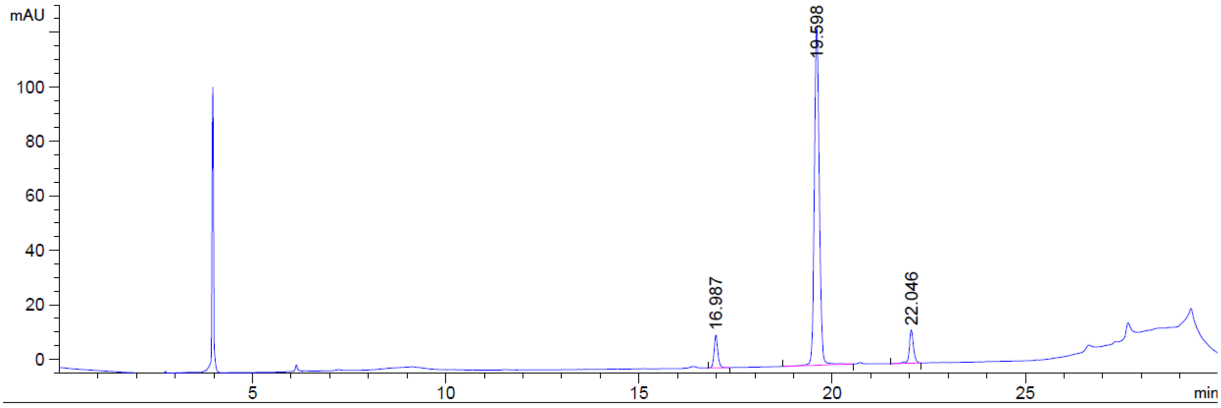

Figure S33. HPLC-chromatogram showing analytical-scale C-acylation of **1c** (*tr* = 17.0 min) into **3c** (t_r = 22.0 min) using ethyl thioacetate (**2a**, t_r = 19.6 min) as an acyl donor with imidazole addition (100 mM).

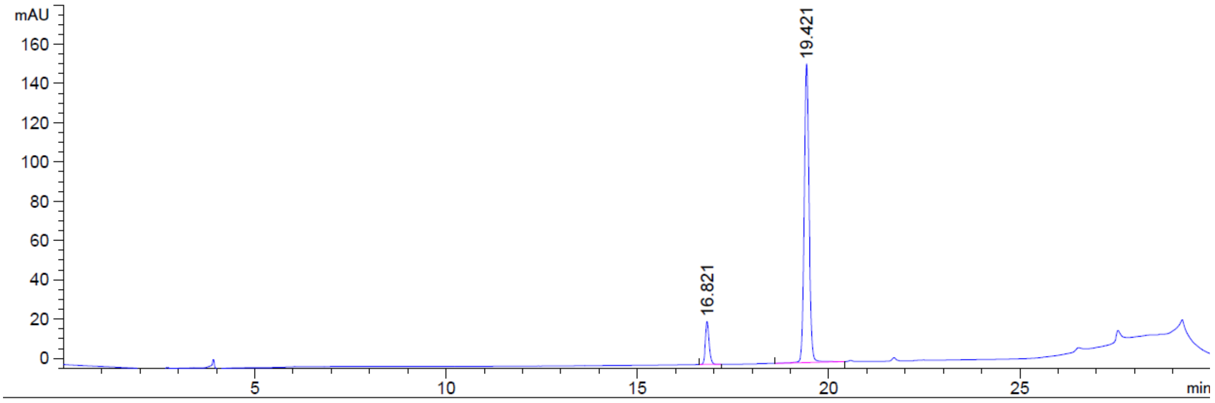

Figure S34. HPLC-chromatogram showing analytical-scale blank reaction of **1c** (*tr* = 16.8 min) using ethyl thioacetate (**2a**, t_r = 19.4 min) as an acyl donor with imidazole addition (100 mM) without enzyme.

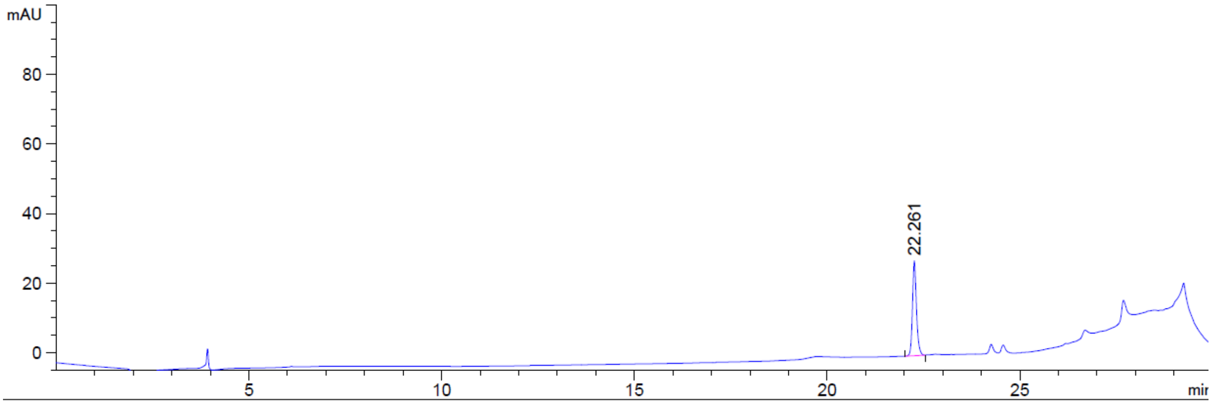

Figure S35. HPLC-chromatogram showing reference compound **3c** (*tr* = 22.3 min).

1-(5-butyl-2,4-dihydroxyphenyl)ethan-1-one (3d)

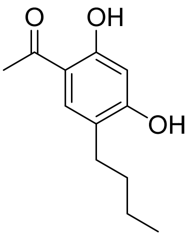

1-(5-butyl-2,4-dihydroxyphenyl)ethan-1-one was obtained from semi-preparative biotransformation as a pale yellow solid with 70% product yield (36.7 mg, 0.176 mmol) after purification *via* column chromatography (silica gel, *c*-hex/EtOAc, 100:0 to 80:20, v/v), m.p. 81-83 °C.

^{1}HNMR (300 MHz, Acetone-*d6*): δ_C [ppm] = 12.61 (s, 1H), 9.37 (s, 1H), 7.65 (s, 1H), 6.35 (s, 1H), 2.64 – 2.52 (m, 5H), 1.67 – 1.48 (m, 2H), 1.38 (dq, *J* = 14.4, 7.2 Hz, 2H), 0.94 (t, *J* = 7.3 Hz, 3H). ^{13}CNMR (75 MHz, Acetone-*d6*): δ_C [ppm] = 202.71, 163.35, 162.56, 132.67, 121.33, 113.13, 102.26, 32.05, 28.96, 25.36, 22.26, 13.34; GC-MS (EI$^+$, 70 eV): *m/z* (%) = 208.1 [M$^+$] (25), 193.1 [$C_{11}H_{13}O_3^+$] (13), 165.0 [$C_9H_9O_3^+$] (100).

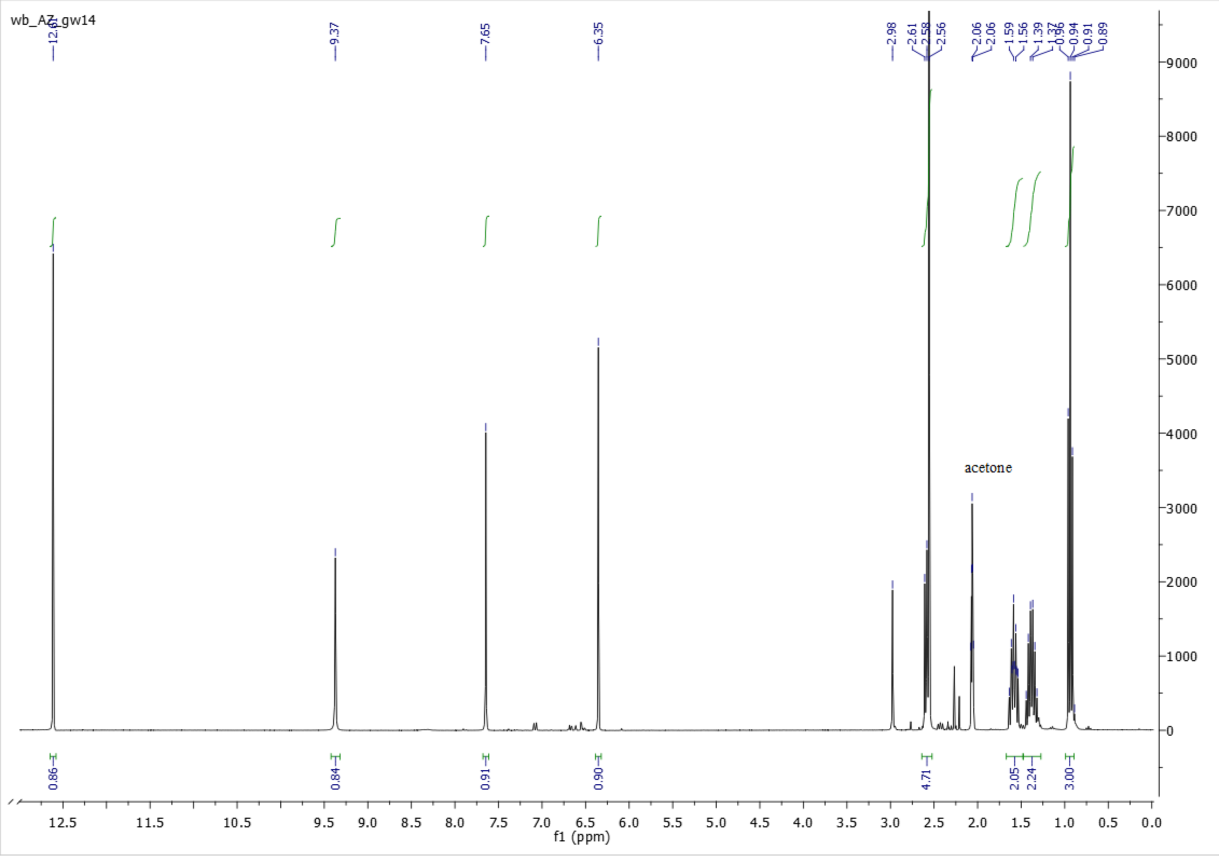

Figure S36. ^{1}HNMR of compound **3d** isolated from the preparative scale bioacylation.

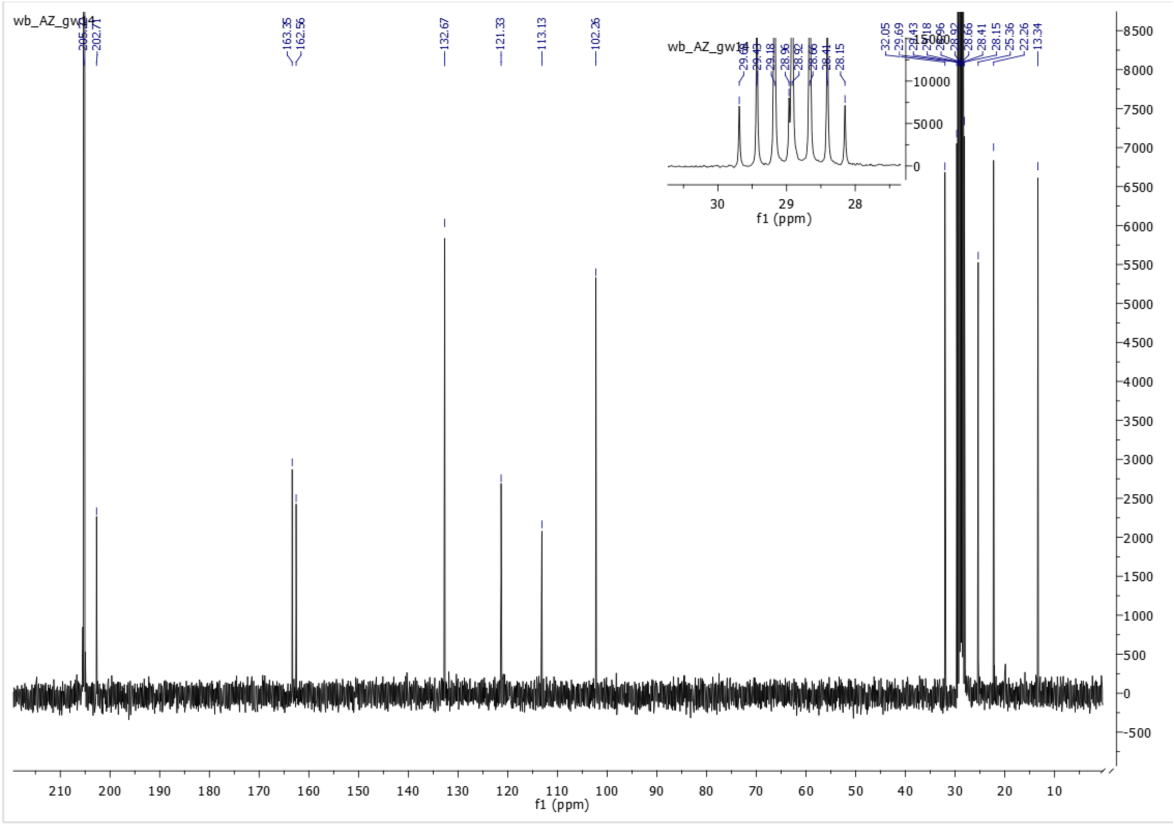

Figure S37. ^{13}CNMR of compound **3d** isolated from the preparative scale bioacylation.

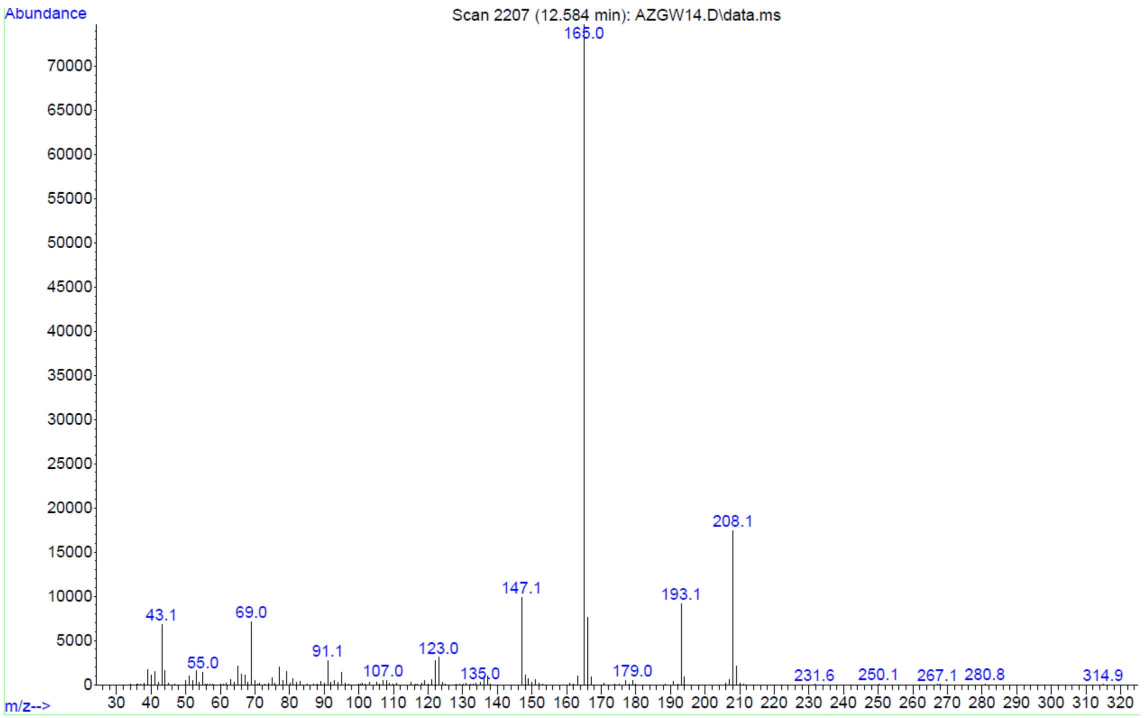

Figure S38. MS of compound **3d** isolated from the preparative scale bioacylation.

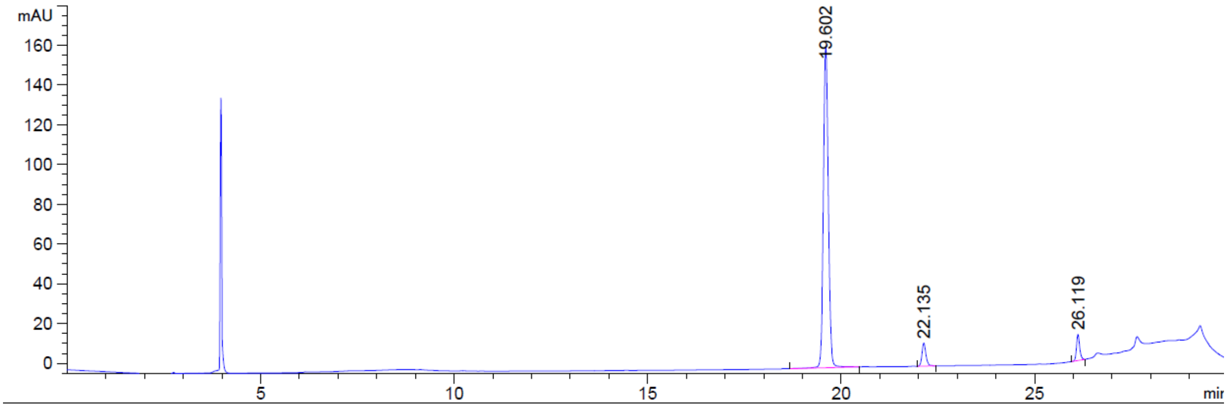

Figure S39. HPLC-chromatogram showing analytical-scale C-acylation of **1d** (*tr* = 22.1 min) into **3d** (t_r = 26.1 min) using ethyl thioacetate (**2a**, t_r = 19.6 min) as an acyl donor without imidazole addition.

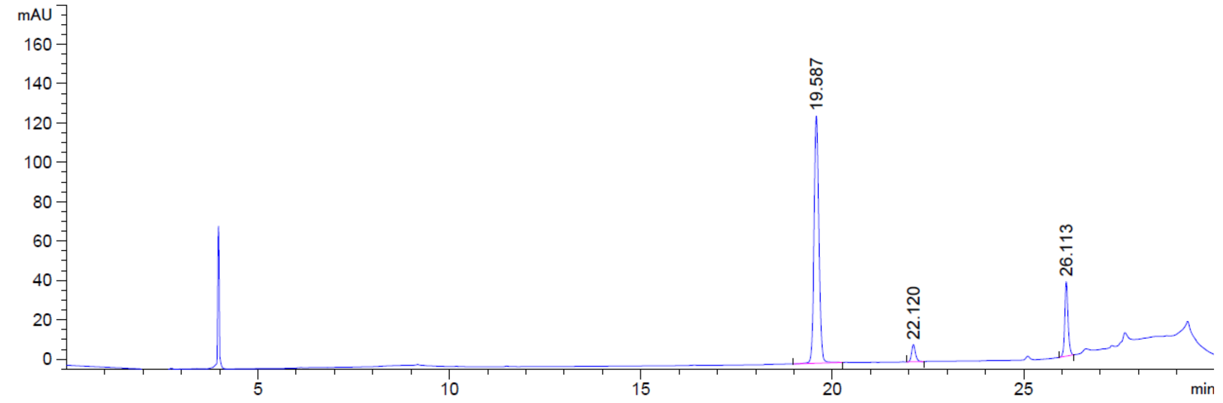

Figure S40. HPLC-chromatogram showing analytical-scale C-acylation of **1d** (*tr* = 22.1 min) into **3d** (t_r = 26.1 min) using ethyl thioacetate (**2a**, t_r = 19.6 min) as an acyl donor with imidazole addition (100 mM).

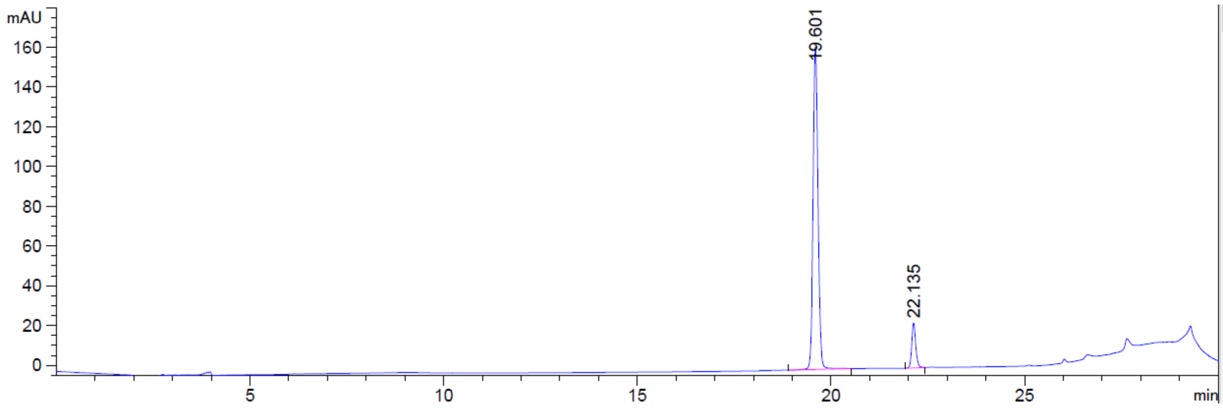

Figure S41. HPLC-chromatogram showing analytical-scale blank reaction of **1d** (*tr* = 22.1 min) using ethyl thioacetate (**2a**, t_r = 19.6 min) as an acyl donor with imidazole addition (100 mM) without enzyme.

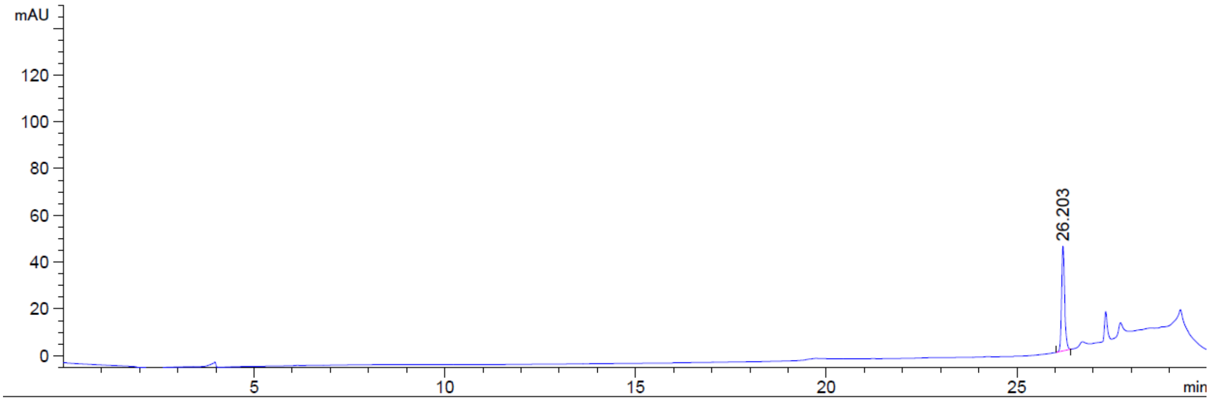

Figure S42. HPLC-chromatogram showing reference compound **3d** (*tr* = 26.2 min).

1-(5-hexyl-2,4-dihydroxyphenyl)ethan-1-one (3e)

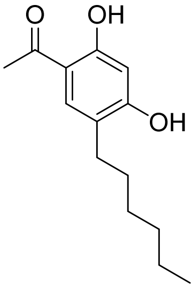

1-(5-hexyl-2,4-dihydroxyphenyl)ethan-1-one was obtained from semi-preparative biotransformation as a white solid with 37% product yield (22.1 mg, 0.094 mmol) after purification *via* column chromatography (silica gel, *c*-hex:EtOAc, 100:0 to 80:20, v/v), m.p. 81-83 °C.

^{1}H NMR (300 MHz, Acetone-*d6*) δ 12.61 (s, 1H), 9.40 (s, 1H), 7.65 (s, 1H), 6.35 (s, 1H), 2.66 – 2.50 (m, 5H), 1.70 – 1.49 (m, 2H), 1.42 – 1.25 (m, 6H), 0.89 (dd, *J* = 8.8, 5.2 Hz, 3H); ^{13}C NMR (75 MHz, Acetone-*d6*): δ [ppm] = 202.69, 163.36, 162.59, 132.66, 121.38, 113.12, 102.26, 31.57, 29.80, 29.69, 29.26, 25.37, 22.40, 13.44; GC-MS (EI$^+$, 70 eV): *m/z* (%) = 236.1 [M$^+$] (15), 221.1 [$C_{13}H_{17}O_3^+$] (7), 165.0 [$C_9H_9O_3^+$] (100).

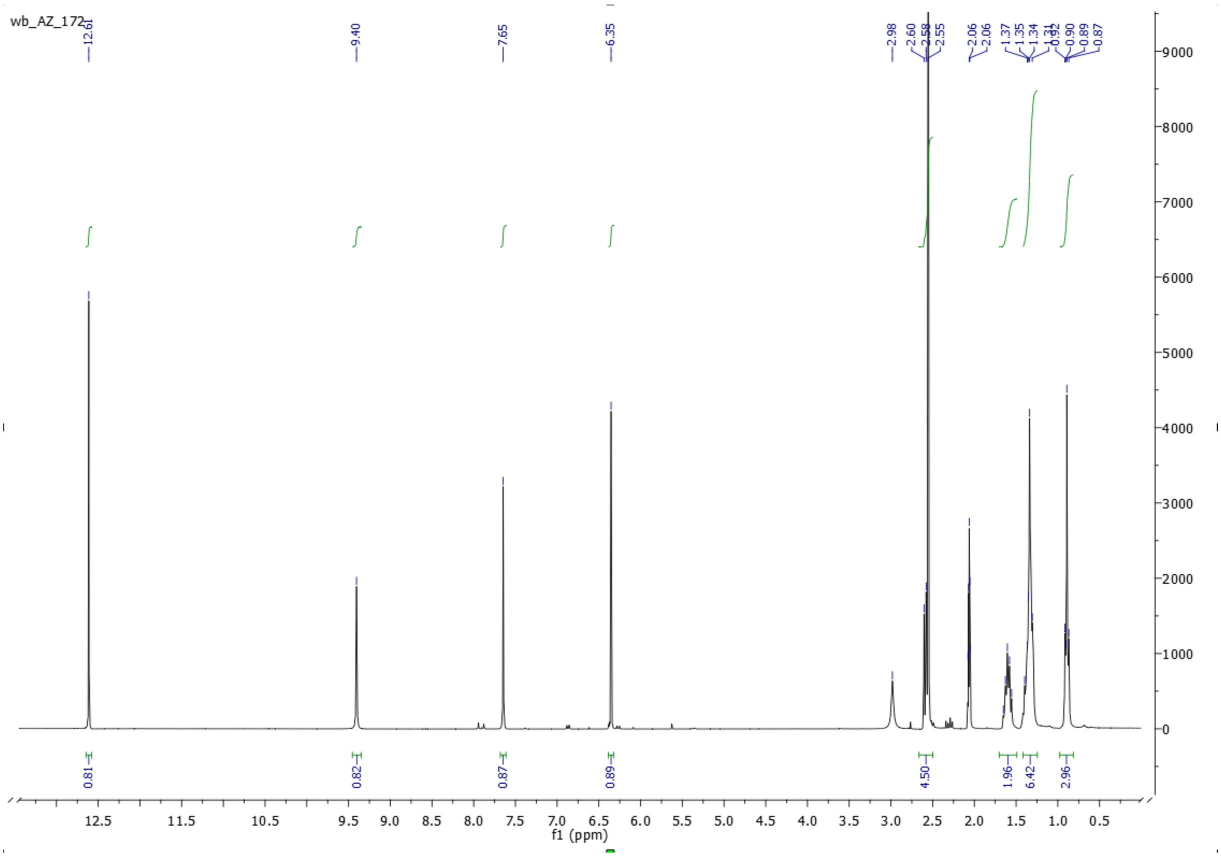

Figure S43. ^{1}HNMR of compound **3e** isolated from the preparative scale bioacylation.

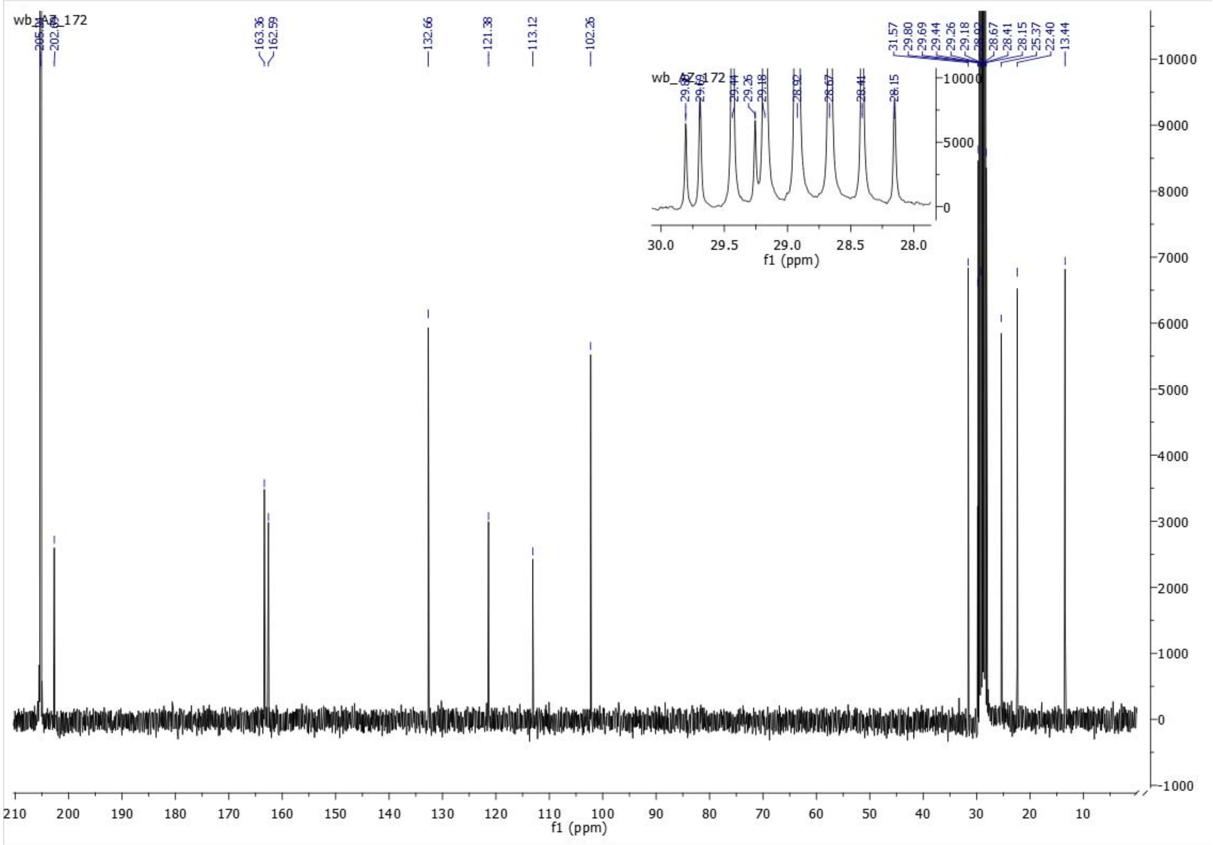

Figure S44. ^{13}CNMR of compound **3e** isolated from the preparative scale bioacylation.

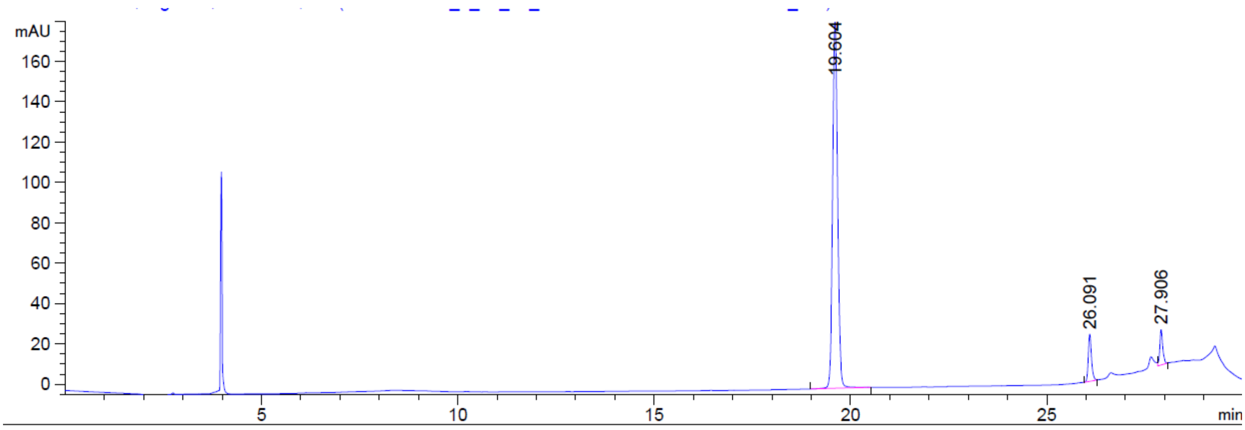

Figure S45. HPLC-chromatogram showing analytical-scale C-acylation of **1e** (*tr* = 26.1 min) into **3e** (t_r = 27.9 min) using ethyl thioacetate (**2a**, t_r = 19.6 min) as an acyl donor without imidazole addition.

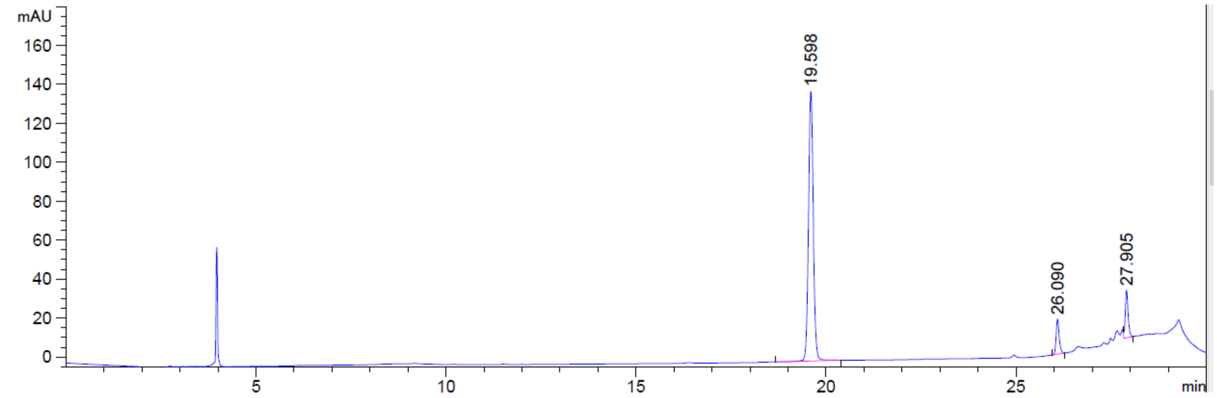

Figure S46. HPLC-chromatogram showing analytical-scale C-acylation of **1e** (*tr* = 26.1 min) into **3e** (t_r = 27.9 min) using ethyl thioacetate (**2a**, t_r = 19.6 min) as an acyl donor with imidazole addition (100 mM).

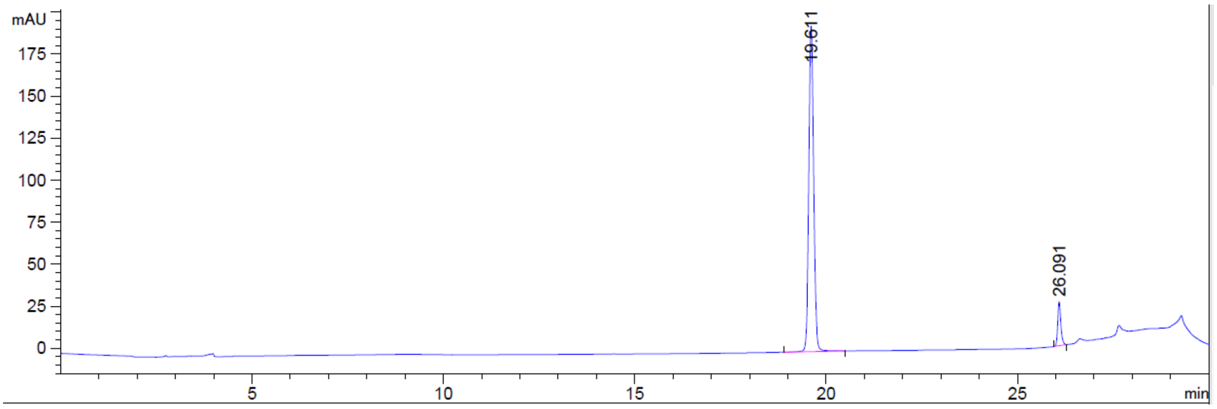

Figure S47. HPLC-chromatogram showing analytical-scale blank reaction of **1e** (*tr* = 26.1 min) using ethyl thioacetate (**2a**, t_r = 19.6 min) as an acyl donor with imidazole addition (100 mM) without enzyme.

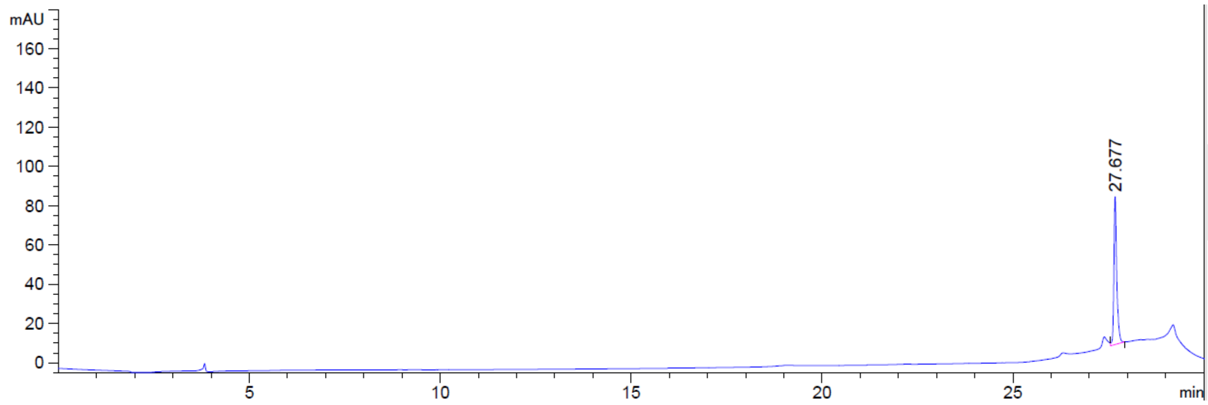

Figure S48. HPLC-chromatogram showing reference compound **3e** (*tr* = 27.7 min).

1-(5-chloro-2,4-dihydroxyphenyl)ethan-1-one (3f)

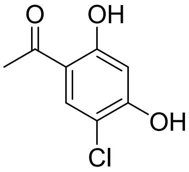

1-(5-chloro-2,4-dihydroxyphenyl)ethan-1-one was obtained from semi-preparative biotransformation as a white solid with 45% product yield (22.1 mg, 0.094 mmol) after purification *via* column chromatography (silica gel, *c*-hex:EtOAc, 100:0 to 80:20, v/v), m.p. 170-171 °C. ^{1}HNMR (300 MHz, Acetone-*d6*): δ [ppm] = 12.58 (s, 1H), 9.99 (s, 1H), 7.91 (s, 1H), 6.51 (s, 1H), 2.62 (s, 3H). ^{13}CNMR (75 MHz, Acetone-*d6*): δ [ppm] = 202.72, 163.37, 159.70, 132.54, 113.94, 111.52, 103.85, 25.55; GC-MS (EI^{+}, 70 eV): *m/z* (%) = 186.0 [M^{+}] (41), 171.0 [$C_7H_4ClO_3^{+}$] (100).

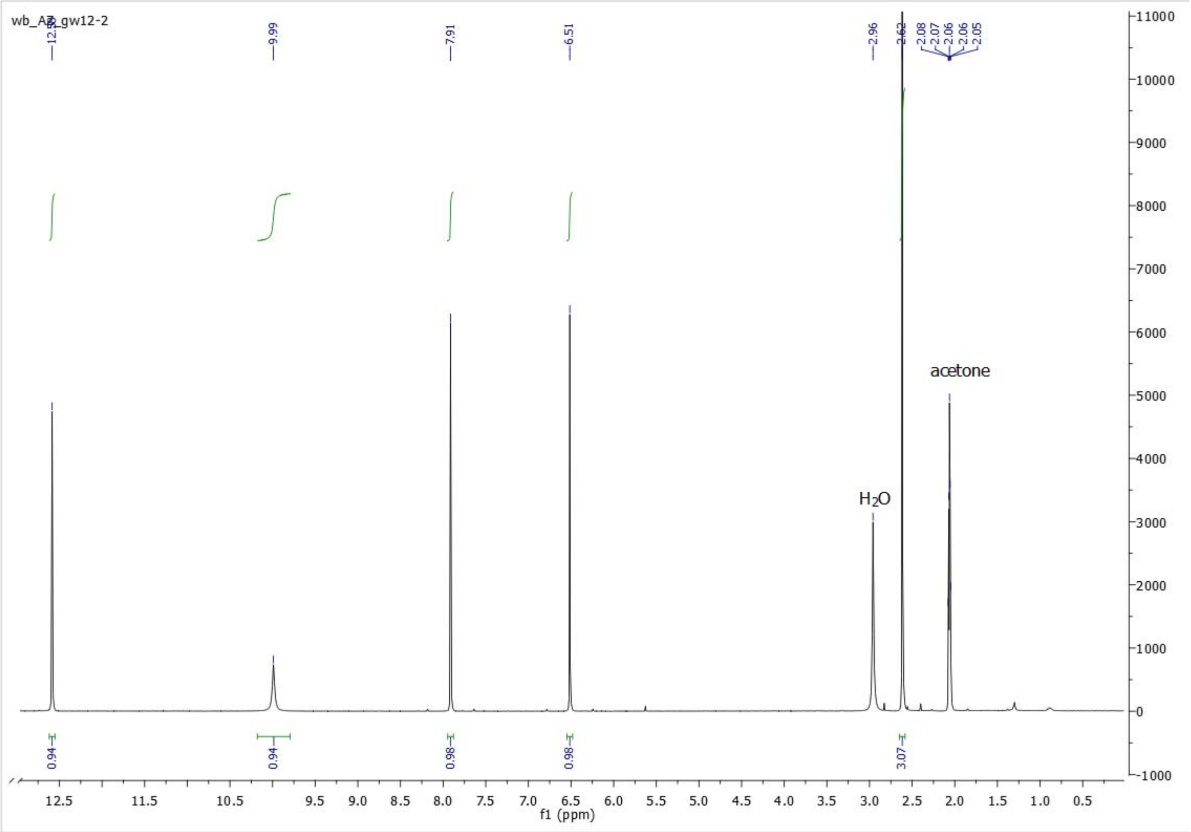

Figure S49. ^{1}HNMR of compound **3f** isolated from the preparative scale bioacylation.

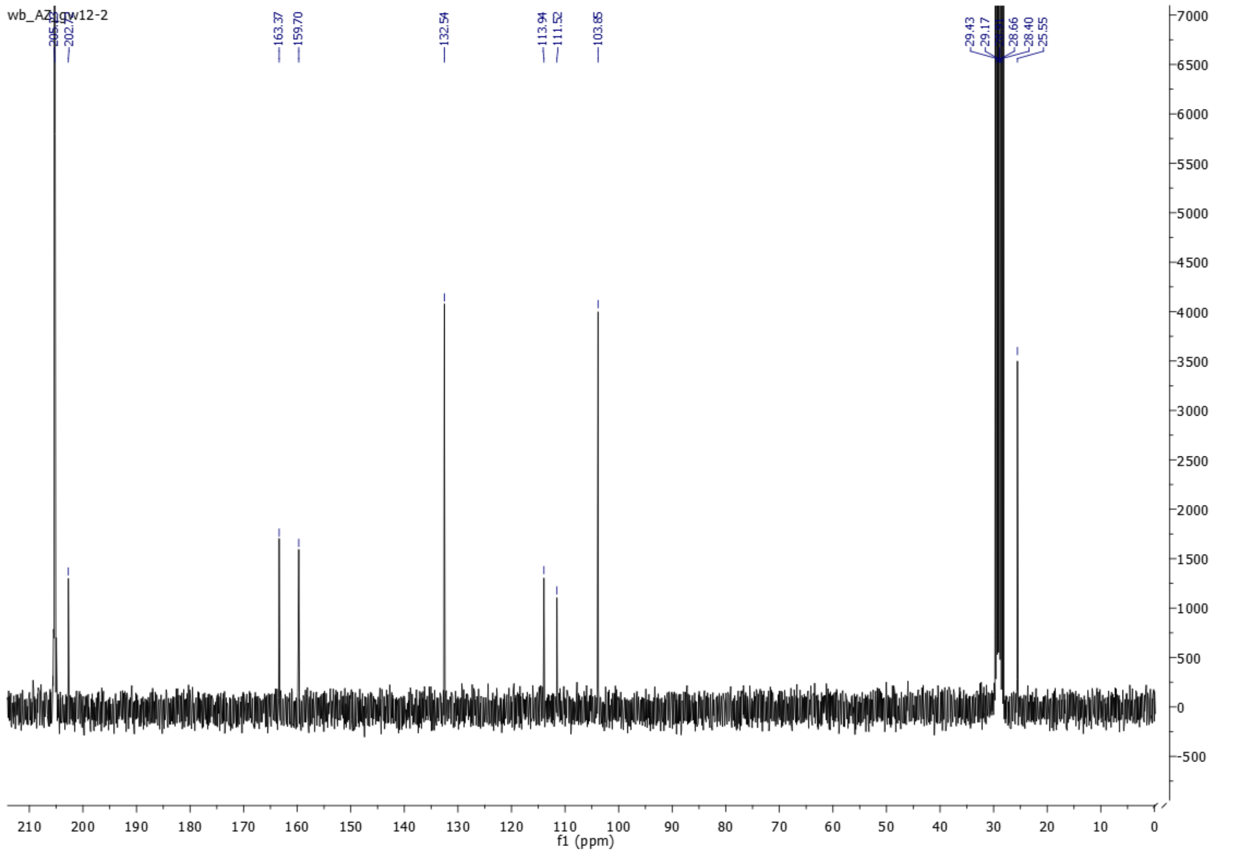

Figure S50. ^{13}CNMR of compound **3f** isolated from the preparative scale bioacylation.

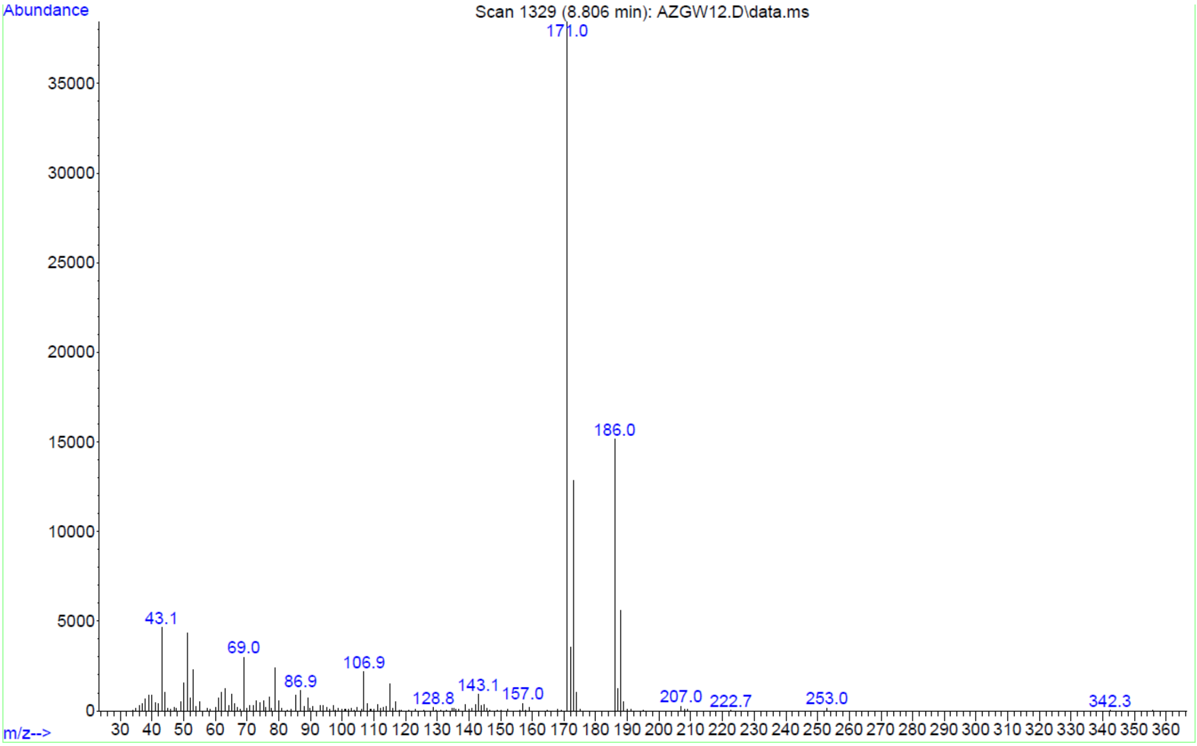

Figure S51. MS of compound **3f** isolated from the preparative scale bioacylation.

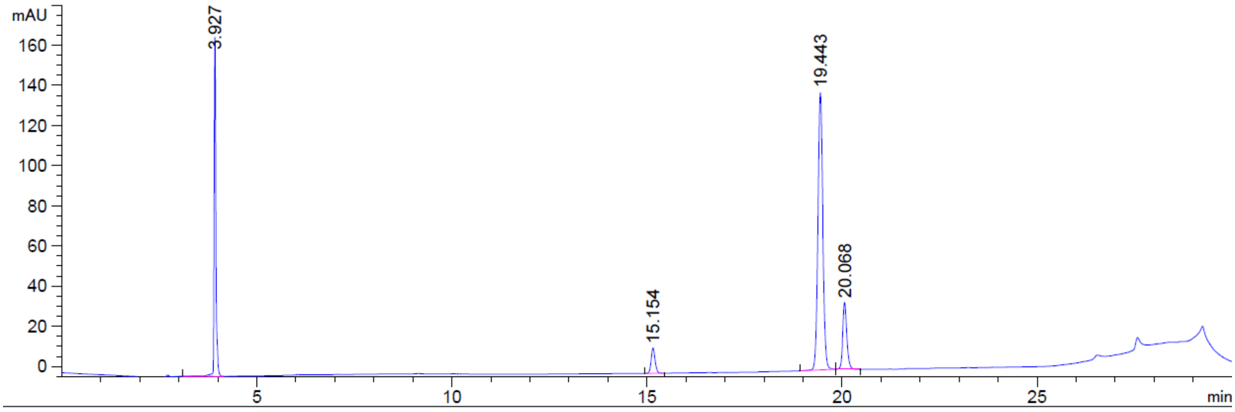

Figure S52. HPLC-chromatogram showing analytical-scale C-acylation of **1f** (*tr* = 15.3 min) into **3f** (t_r = 20.2 min) using ethyl thioacetate (**2a**, t_r = 19.6 min) as an acyl donor without imidazole addition.

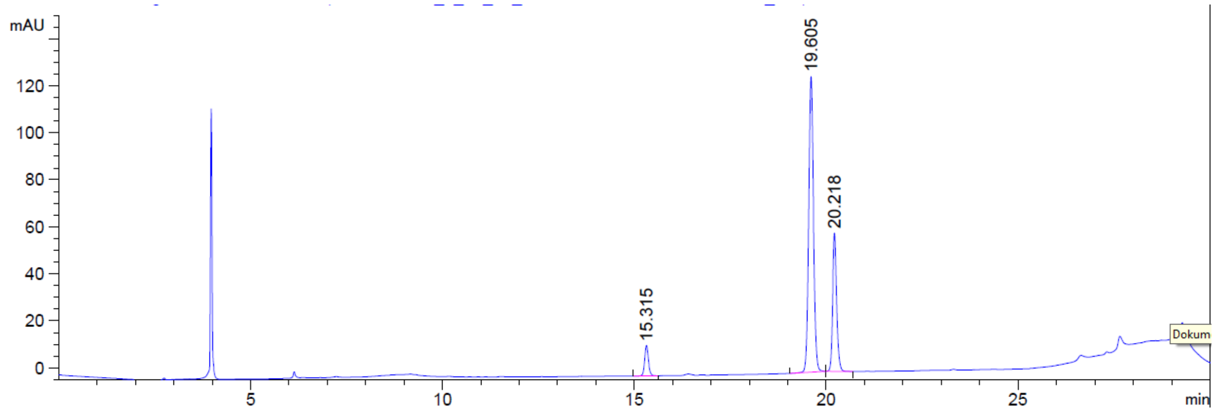

Figure S53. HPLC-chromatogram showing analytical-scale C-acylation of **1f** (*tr* = 15.3 min) into **3f** (t_r = 20.2 min) using ethyl thioacetate (**2a**, t_r = 19.6 min) as an acyl donor with imidazole addition (100 mM).

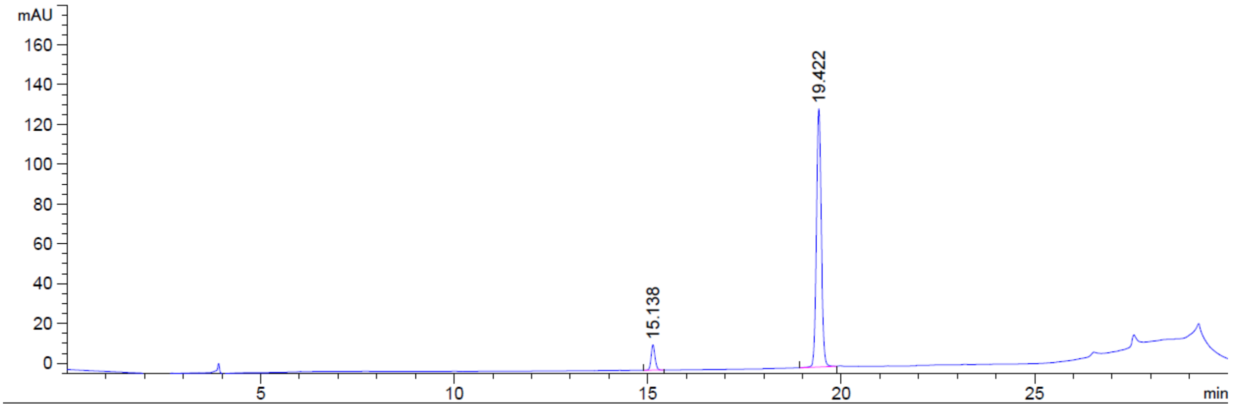

Figure S54. HPLC-chromatogram showing analytical-scale blank reaction of **1f** (*tr* = 15.3 min) using ethyl thioacetate (**2a**, t_r = 19.4 min) as an acyl donor with imidazole addition (100 mM) without enzyme.

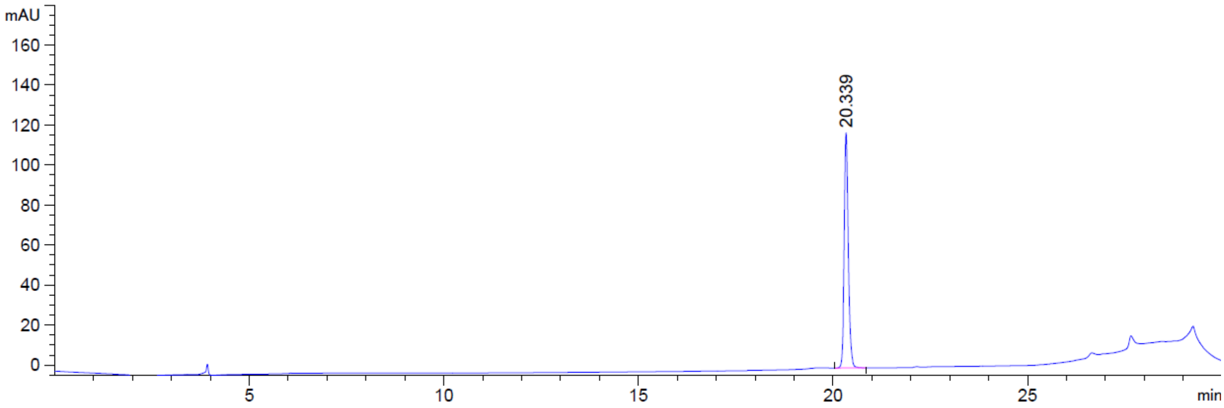

Figure S55. HPLC-chromatogram showing reference compound **3f** (*tr* = 20.3 min).

N-phenylacetamide (3i)

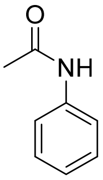

N-phenylacetamide was obtained from semi-preparative biotransformation as a white solid with 37% product yield (12.4 mg, 0.092 mmol) after purification *via* column chromatography (silica gel, *c*-hex:EtOAc, 100:0 to 80:20, v/v), m.p. 113-114 °C (lit. 113-115 °C)[7,8]. ^{1}HNMR (300 MHz, $CDCl_3$): δ [ppm] = 7.52 (d, J = 7.9 Hz, 2H), 7.34 (t, J = 7.9 Hz, 2H), 7.12 (t, J = 7.4 Hz, 1H), 2.19 (s, 3H). ^{13}CNMR (75 MHz, $CDCl_3$): δ [ppm] = 168.24, 137.85, 128.99, 124.30, 119.85, 24.60; GC-MS (EI^+, 70 eV): *m/z* (%) = 135.1 [M^+] (27), 93.1 [$C_6H_7N^+$] (100).

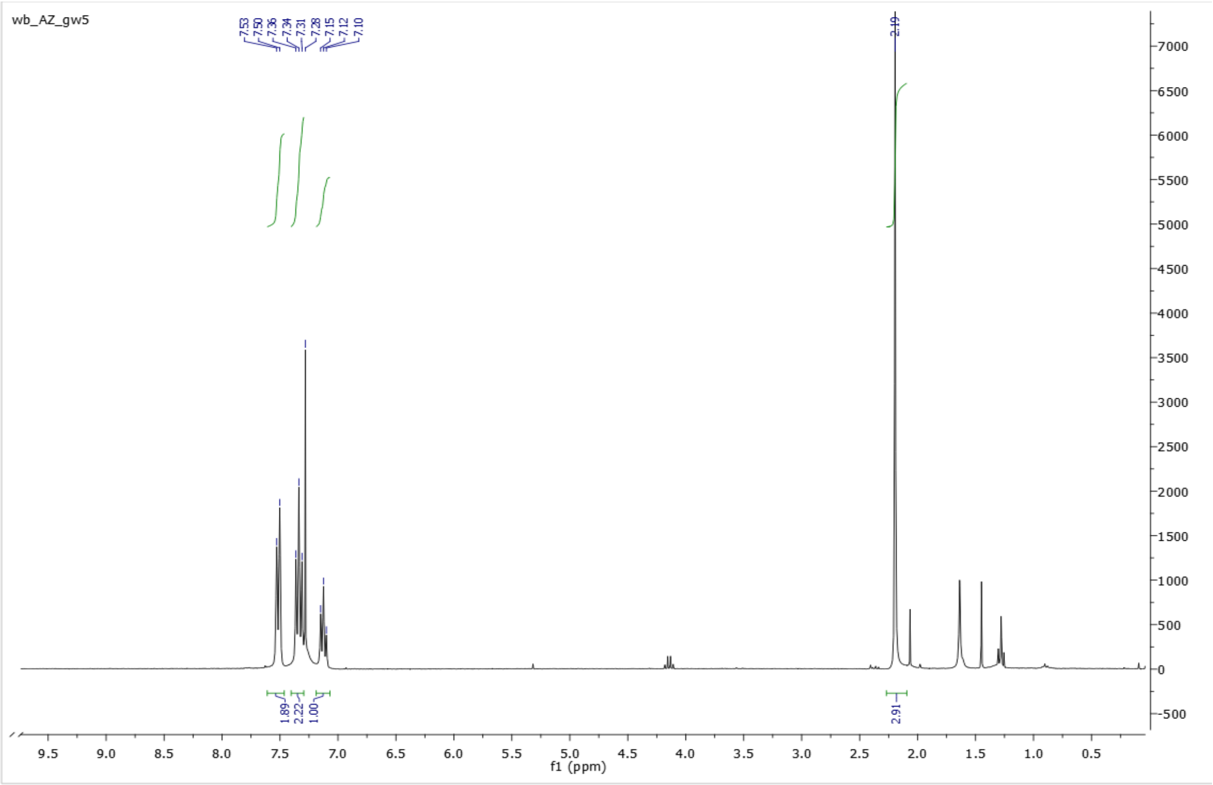

Figure S56. ^{1}HNMR of compound **3i** isolated from the preparative scale bioacylation.

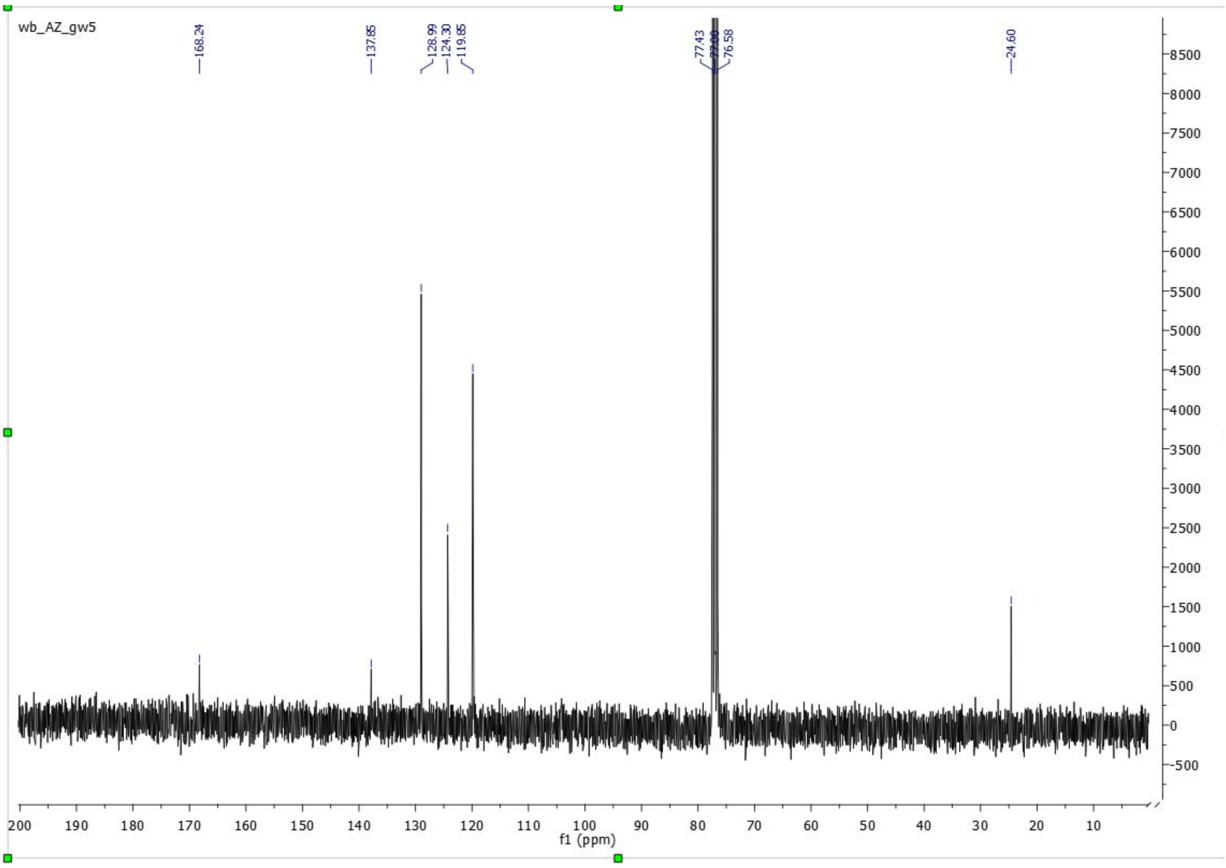

Figure S57. ^{13}CNMR of compound **3i** isolated from the preparative scale bioacylation.

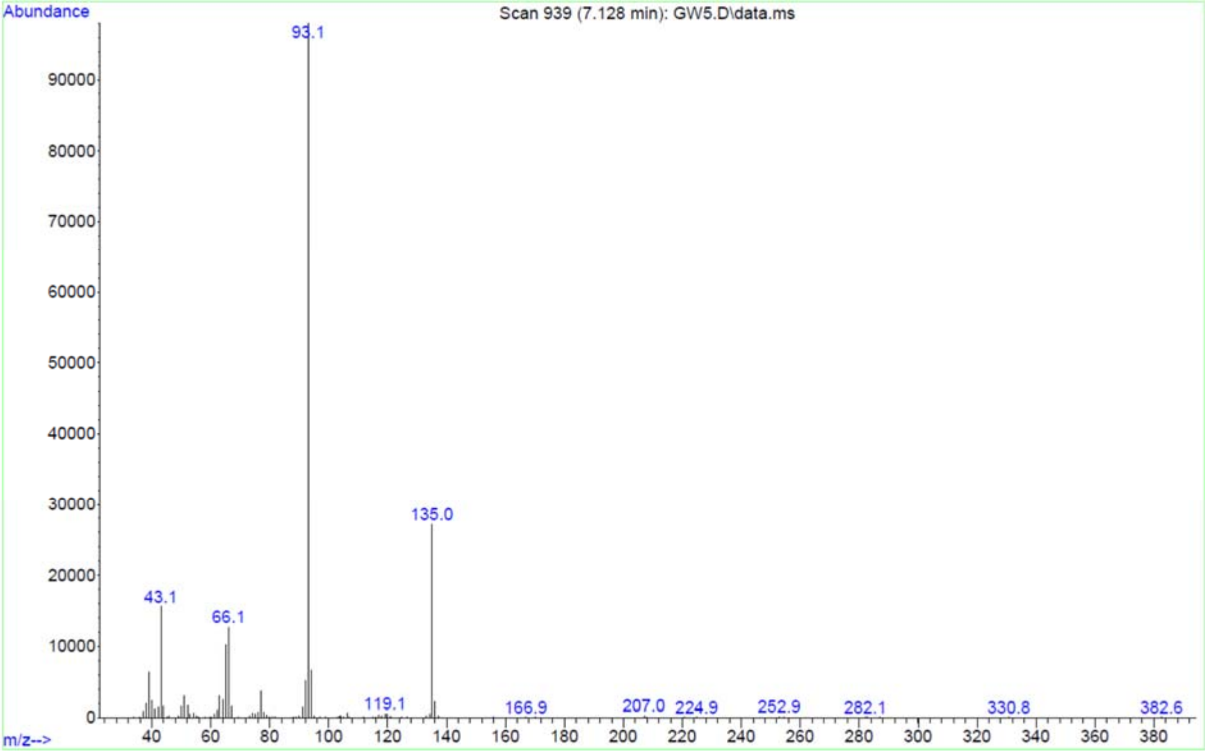

Figure S58. MS of compound **3i** isolated from the preparative scale bioacylation.

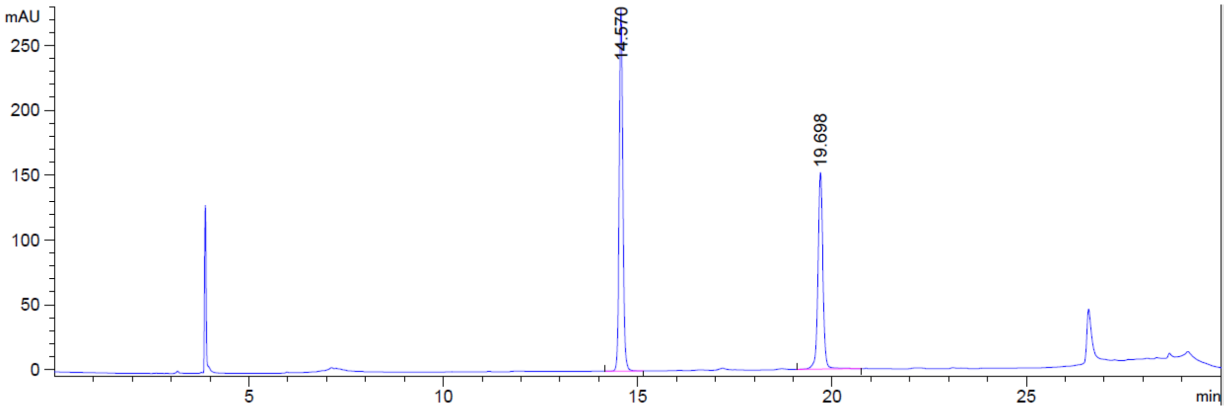

Figure S59. HPLC-chromatogram showing analytical-scale C-acylation of **1i** into **3i** (t_r = 14.6 min) using ethyl thioacetate (**2a**, t_r = 19.7 min) as an acyl donor without imidazole addition.

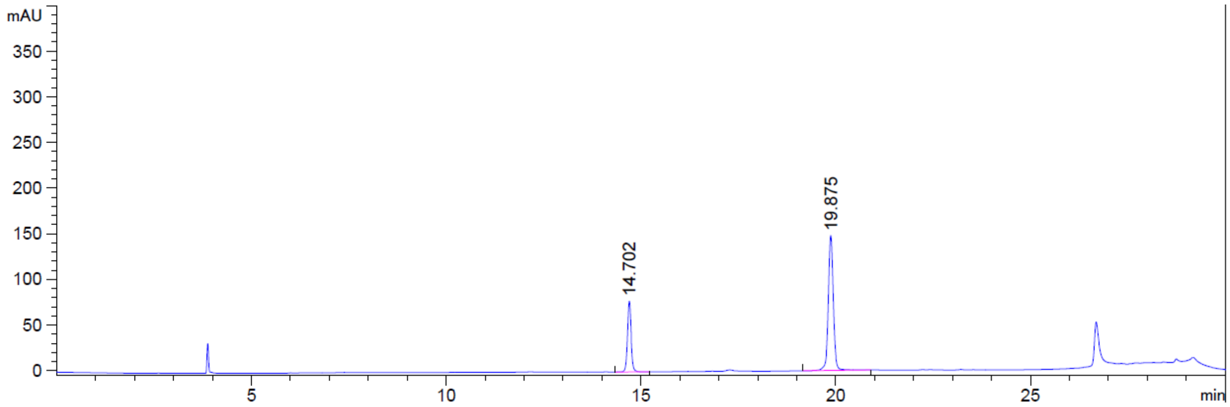

Figure S60. HPLC-chromatogram showing analytical-scale blank reaction of **1i** using ethyl thioacetate (**2a**, t_r = 19.9 min) as an acyl donor without imidazole addition and without enzyme. Spontaneous formation of the trace amount of product **3i** was noticed (t_r = 14.7 min).

N-(3-hydroxyphenyl)acetamide (3j)

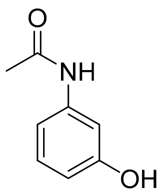

N-(3-hydroxyphenyl)acetamide was obtained from semi-preparative biotransformation as a light brown solid with 58% product yield (22 mg, 0.146 mmol) after purification *via* column chromatography (silica gel, *c*-hex:EtOAc, 100:0 to 80:20, v/v), m.p. 137-139 °C (lit. 146-148 °C)[7,8]. ^{1}H-NMR and MS-data are in accordance with literature.[9]

^{1}HNMR (300 MHz, DMSO-*d6*): δ [ppm] 9.77 (s, 1H), 9.31 (s, 1H), 7.18 (t, J = 1.9 Hz, 1H), 7.04 (t, J = 8.0 Hz, 1H), 6.92 (d, J = 8.1 Hz, 1H), 6.49 – 6.32 (m, 1H), 2.01 (s, 3H); ^{13}CNMR (75 MHz, DMSO-*d6*): δ [ppm] 168.58, 157.98, 140.80, 129.69, 110.54, 110.19, 106.61, 24.50; GC-MS (EI^{+}, 70 eV): *m/z* (%) = 151.1 [M^{+}] (44), 109.1 [$C_6H_7NO^{+}$] (100).

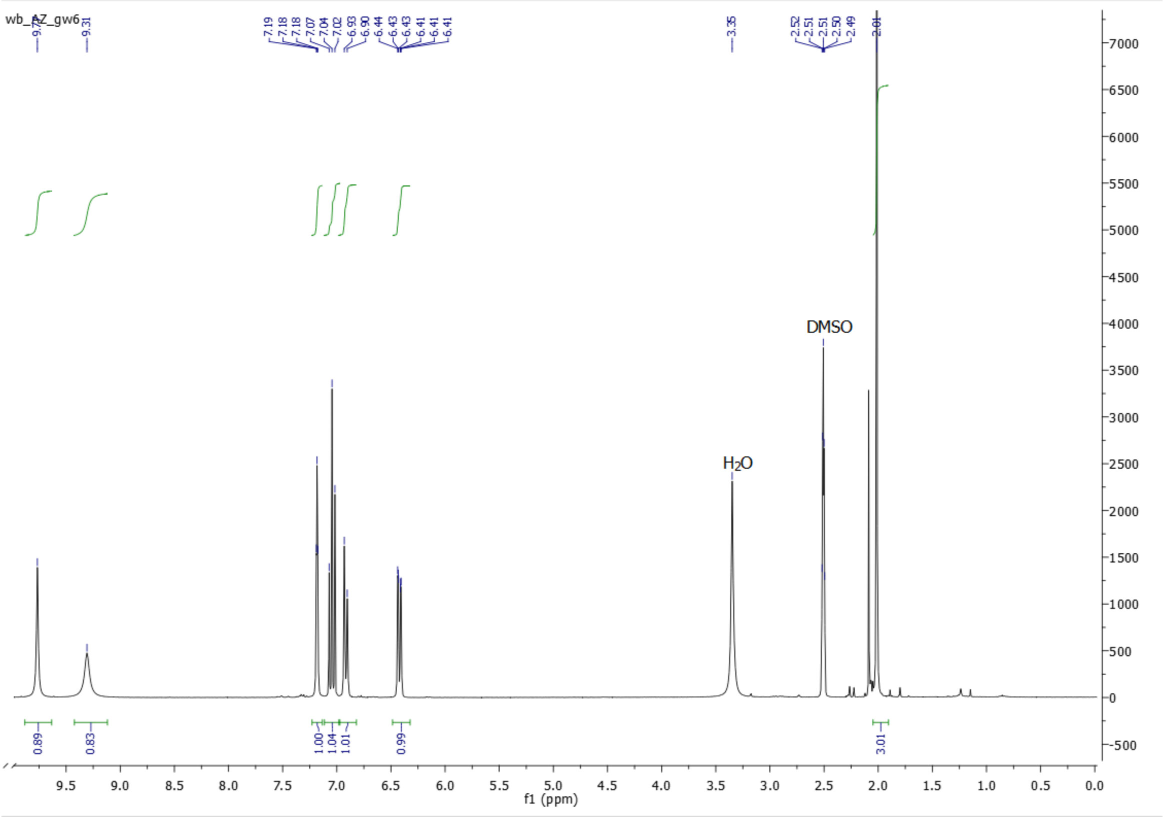

Figure S61. ^{1}HNMR of compound **3j** isolated from the preparative scale bioacylation.

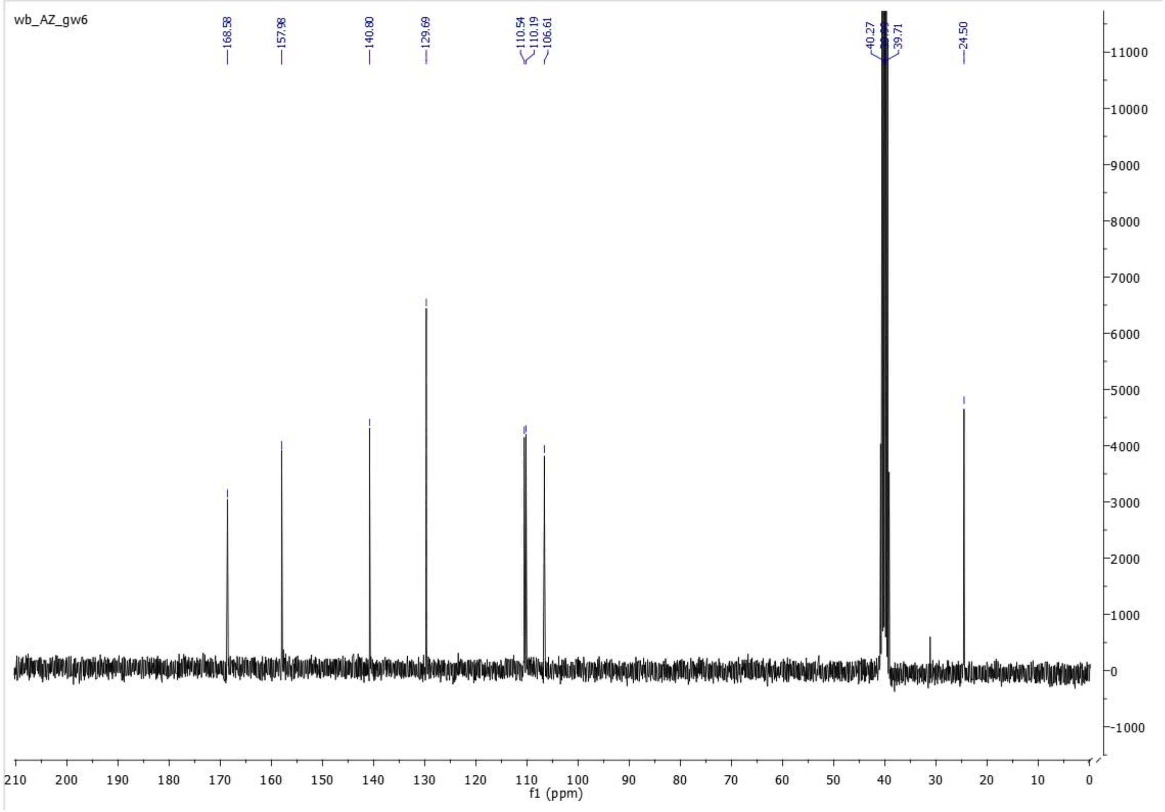

Figure S62. ^{13}CNMR of compound **3j** isolated from the preparative scale bioacylation.

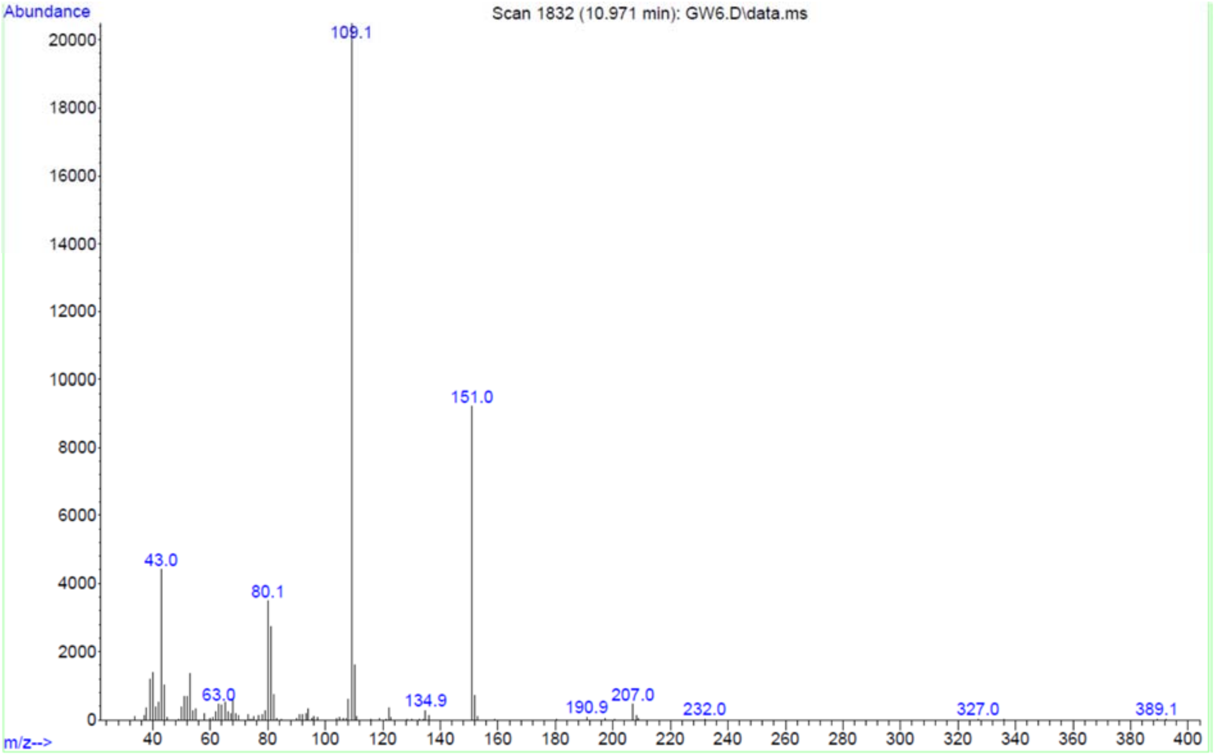

Figure S63. MS of compound **3j** isolated from the preparative scale bioacylation.

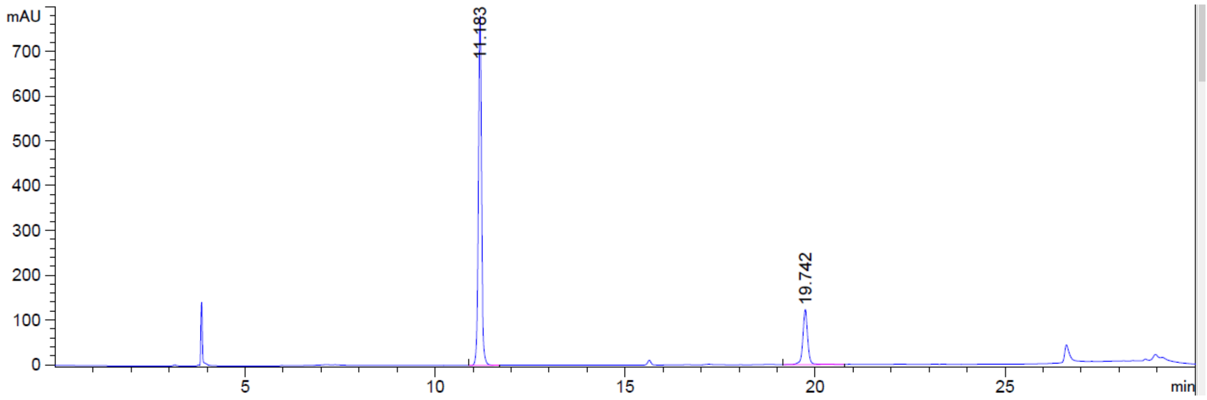

Figure S64. HPLC-chromatogram showing analytical-scale C-acylation of **1j** into **3j** (t_r = 11.2 min) using ethyl thioacetate (**2a**, t_r = 19.7 min) as an acyl donor without imidazole addition.

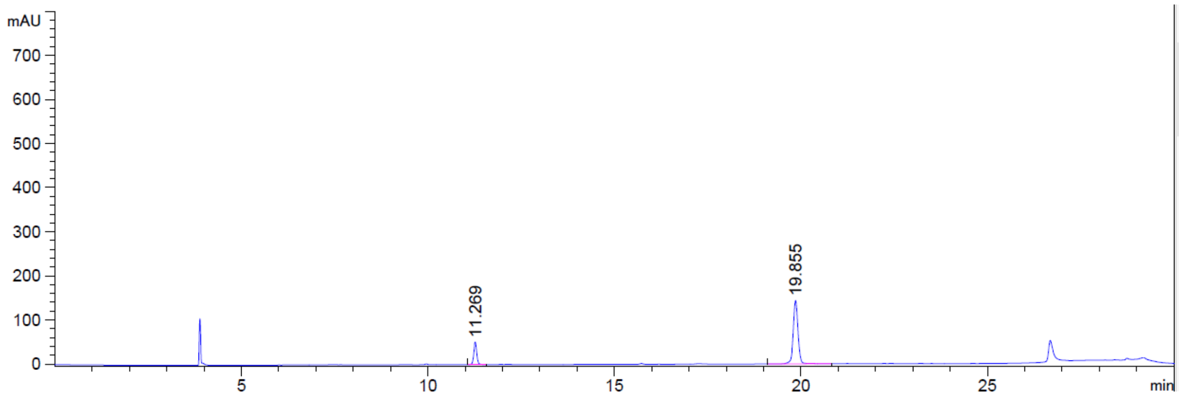

Figure S65. HPLC-chromatogram showing analytical-scale blank reaction of **1j** using ethyl thioacetate (**2a**, t_r = 19.9 min) as an acyl donor without imidazole addition and without enzyme. Spontaneous formation of the trace amount of product **3j** was noticed (t_r = 11.3 min).

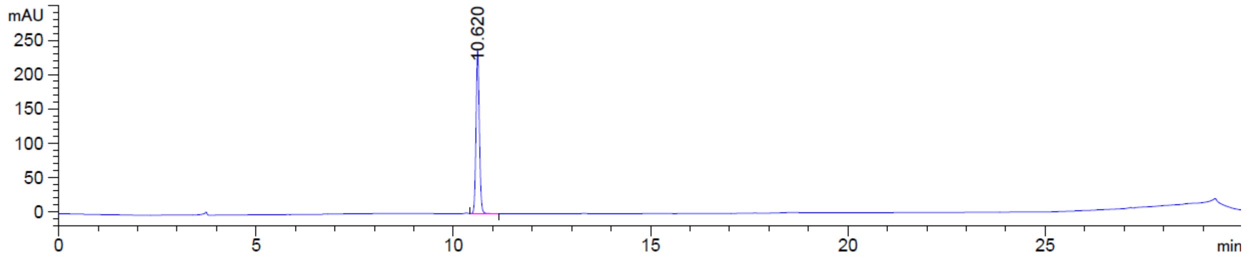

Figure S66. HPLC-chromatogram showing reference compound **3j** (*tr* = 10.6 min).

References

[1] (a) N. G. Schmidt, T. Pavkov-Keller, N. Richter, B. Wiltschi, K. Gruber, W. Kroutil, *Angew. Chem. Int. Ed.* **2017**, *56*, 7615-7619; (b) N. G. Schmidt, W. Kroutil, *Eur. J. Org. Chem.* **2017**, *39*, 5865-5871.

[2] W. Shi, W.-J. Dan, J.-J. Tang, Y. Zhang, T. Nandinsuren, A.-L. Zhang, J.-M. Gao, *Bioorg. Med. Chem. Lett.* **2016**, *26*, 2156-2158.

[3] D. K. Herron, T. Goodson, N. G. Bollinger, D. Swanson-Bean, I. G. Wright, G. S. Staten, A. R. Thompson, L. L. Froelich, W. T. Jackson, *J. Med. Chem.* **1992**, *35*, 1818-1828.

[4] P. A. Brough, W. Aherne, X. Barril, J. Borgognoni, K. Boxall, J. E. Cansfield, K.-M. J. Cheung, I. Collins, N. G. M. Davies, M. J. Drysdale, B. Dymock, S. A. Eccles, H. Finch, A. Fink, A. Hayes, R. Howes, R. E. Hubbard, K. James, A. M. Jordan, A. Lockie, V. Martins, A. Massey, T. P. Matthews, E. McDonald, C. J. Northfield, L. H. Pearl, C. Prodromou, S. Ray, F. I. Raynaud, S. D. Roughley, S. Y. Sharp, A. Surgenor, D. L. Walmsley, P. Webb, M. Wood, P. Workman, L. Wright, *J. Med. Chem.* **2007**, *51*, 196-218.

[5] S. Mizobuchi, Y. Sato, *Agric. and Bio. Chem.* **1985**, *49*, 1327-1333.

[6] P. Liu, X. Xu, L. Chen, L. Ma, X. Shen, L. Hu, *Bioorg. Med. Chem.* **2014**, *22*, 1596-1607.

[7] M. J. Fifolt, R. T. Olczak, R. F. Mundhenke, J. F. Bieron, *J. Org. Chem.* **1985**, *50*, 4576-4582.

[8] G. Brahmachari, S. Laskar, S. Sarkar, *J. Chem. Res.* **2010**, *34*, 288-295.

[9] S. S. van Berkel, B. van der Lee, F. L. van Delft, F. P. Rutjes, *Chem. Comm.* **2009**, *28,* 4272-4274.
